# First-in-human phase 1 clinical trial of anti-core 1 O-glycans targeting monoclonal antibody NEO-201 in treatment-refractory solid tumors

**DOI:** 10.1186/s13046-023-02649-6

**Published:** 2023-03-29

**Authors:** Christopher B. Cole, Maria Pia Morelli, Massimo Fantini, Markku Miettinen, Patricia Fetsch, Cody Peer, William D. Figg, Tyler Yin, Nicole Houston, Ann McCoy, Stanley Lipkowitz, Alexandra Zimmer, Jung-min Lee, Miroslava Pavelova, Erin N. Villanueva, Kathryn Trewhitt, B. Brooke Solarz, Maria Fergusson, Sharon A. Mavroukakis, Anjum Zaki, Kwong Y. Tsang, Philip M. Arlen, Christina M. Annunziata

**Affiliations:** 1 Women’s Malignancies Branch, Center for Cancer Research, National Cancer Institute, National Institutes of Health, Bethesda, MD USA; 2 Precision Biologics, Inc, Bethesda, MD USA; 3 Laboratory of Pathology, Center for Cancer Research, National Cancer Institute, National Institutes of Health, Bethesda, MD USA; 4 Clinical Pharmacology Program, National Cancer Institute, National Institutes of Health, Bethesda, MD USA

**Keywords:** Cancer immunotherapy, Monoclonal antibody, O-glycan, Antibody-dependent cellular cytotoxicity, NEO-201, Regulatory T cells, Clinical trial

## Abstract

**Background:**

NEO201 is a humanized IgG1 monoclonal antibody (mAb) generated against tumor-associated antigens from patients with colorectal cancer. NEO-201 binds to core 1 or extended core 1 O-glycans expressed by its target cells. Here, we present outcomes from a phase I trial of NEO-201 in patients with advanced solid tumors that have not responded to standard treatments.

**Methods:**

This was a single site, open label 3 + 3 dose escalation clinical trial. NEO-201 was administered intravenously every two weeks in a 28-day cycle at dose level (DL) 1 (1 mg/kg), DL 1.5 (1.5 mg/kg) and DL 2 (2 mg/kg) until dose limiting toxicity (DLT), disease progression, or patient withdrawal. Disease evaluations were conducted after every 2 cycles. The primary objective was to assess the maximum tolerated dose (MTD) and recommended phase 2 dose (RP2D) of NEO-201. The secondary objective was to assess the antitumor activity by RECIST v1.1. The exploratory objectives assessed pharmacokinetics and the effect of NEO-201 administration on immunologic parameters and their impact on clinical response.

**Results:**

Seventeen patients (11 colorectal, 4 pancreatic and 2 breast cancers) were enrolled; 2 patients withdrew after the first dose and were not evaluable for DLT. Twelve of the 15 patients evaluable for safety discontinued due to disease progression and 3 patients discontinued due to DLT (grade 4 febrile neutropenia [1 patient] and prolonged neutropenia [1 patient] at DL 2, and grade 3 prolonged (> 72 h) febrile neutropenia [1 patient] at DL 1.5). A total of 69 doses of NEO-201 were administered (range 1–15, median 4). Common (> 10%) grade 3/4 toxicities occurred as follows: neutropenia (26/69 doses, 17/17 patients), white blood cell decrease (16/69 doses, 12/17 patients), lymphocyte decrease (8/69 doses, 6/17 patients). Thirteen patients were evaluable for disease response; the best response was stable disease (SD) in 4 patients with colorectal cancer. Analysis of soluble factors in serum revealed that a high level of soluble MICA at baseline was correlated with a downregulation of NK cell activation markers and progressive disease. Unexpectedly, flow cytometry showed that NEO-201 also binds to circulating regulatory T cells and reduction of the quantities of these cells was observed especially in patients with SD.

**Conclusions:**

NEO-201 was safe and well tolerated at the MTD of 1.5 mg/kg, with neutropenia being the most common adverse event. Furthermore, a reduction in the percentage of regulatory T cells following NEO-201 treatment supports our ongoing phase II clinical trial evaluating the efficiency of the combination of NEO-201 with the immune checkpoint inhibitor pembrolizumab in adults with treatment-resistant solid tumors.

**Trial registration:**

NCT03476681. Registered 03/26/2018.

**Graphical Abstract:**

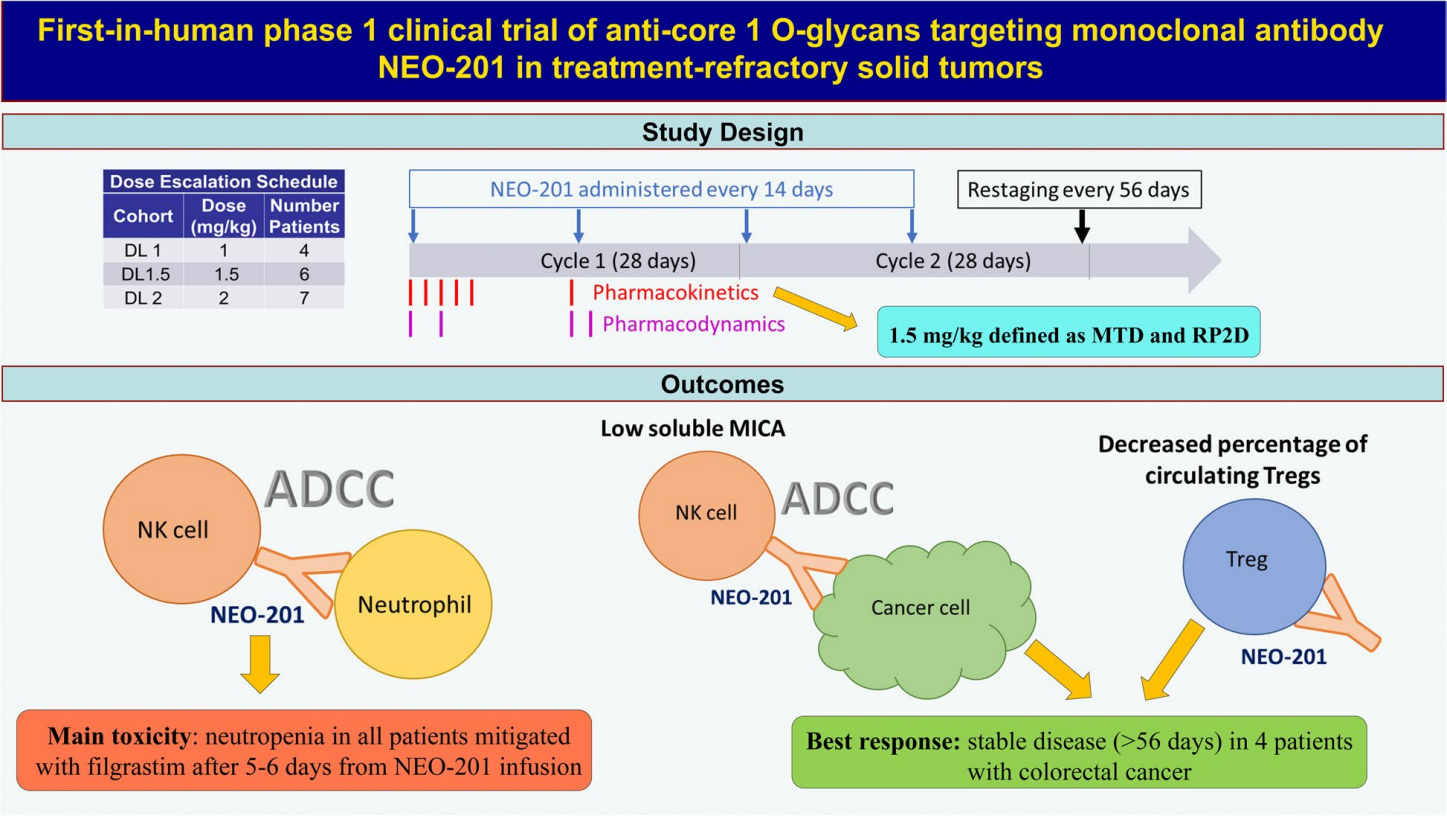

**Supplementary Information:**

The online version contains supplementary material available at 10.1186/s13046-023-02649-6.

## Background

Recent efforts in cancer therapeutics have focused on the development of drugs that activate the immune system against cancer cells to achieve durable disease control. In recent decades, cancer immunotherapies have emerged as promising treatment options for many cancer patients and have increasingly been employed in specific disease settings as either an alternative to traditional chemotherapy and radiotherapy or as adjunctive therapy to these traditional modalities providing additive or even synergistic activity. Most cancer immunotherapies, including immune checkpoint inhibitors, vaccines, and engineered chimeric T-cell receptors, have focused on boosting the adaptive immune system in its activities of immunosurveillance, allowing it to recognize and mount immune responses against tumors [1]. Immune checkpoint inhibitors (ICIs) are monoclonal antibodies (mAbs) that have been developed to enhance antitumor activity by blocking immunosuppressive immune checkpoints and have received FDA approval for the treatment of certain tumor types [2]. Although immunotherapy with mAbs has increased survival of cancer patients, response rates for these agents vary widely and there is a significant portion of patients that fails to respond [3, 4].

Concurrently with the development of immune checkpoint inhibitors, other mAbs, capable of recognizing tumor-associated antigens and directly eliciting tumor cell killing via antibody-dependent cellular cytotoxicity (ADCC) and/or complement-dependent cytotoxicity (CDC), have been developed [5, 6]. Examples of FDA approved mAbs that can mediate ADCC include trastuzumab (HER2^+^ breast cancer) [7], rituximab (multiple lymphomas) [8], cetuximab (advanced and metastatic colorectal cancer and head and neck cancer) [9], and avelumab (metastatic Merkel cell carcinoma in adults and pediatric patients aged ≥ 12 years and as maintenance treatment of patients with locally advanced or metastatic urothelial carcinoma) [10, 11]. Many approved mAbs have clinical activity as single agents, with efforts underway to combine them with other therapies such as immune checkpoint inhibitors [12].

NEO-201 is a humanized IgG1 mAb generated against tumor-associated antigens from patients with colorectal cancer [13]. In previous studies, we observed that NEO-201 targets tumors expressing tumor-associated carcinoembryonic antigen-related cell adhesion molecules (CEACAM)-5 and CEACAM-6 variants but does not bind to those expressed in healthy tissues [13–15]. In line with this, we observed that the NEO-201 target antigen is present, as tested by immunohistochemistry (IHC), in the majority of epithelial-derived cancers, including colon (85%), pancreas (86%), adenocarcinoma of the lung (79%), squamous cell lung, breast cancer (53%), and mucinous and signet cell ovarian cancer (> 50%), but is not present in surrounding stromal tissue or in healthy control samples from the affected organs [13–15].

NEO-201 binding specificity for tumor-associated proteins could be due to the presence of glycan patterns (i.e. O-glycans) attached to the protein carriers during post-translational modifications made during the process of carcinogenesis [16]. In this regard, we recently showed that NEO-201 binds specifically to core 1 and/or extended core 1 O-glycans expressed by NEO-201-target cells, such as human pancreatic cancer cell line CFPAC-1, acute myeloid leukemia (AML) cell lines HL-60 and U937, and human neutrophils. Since HL-60 and U937 do not express CEACAM-5 or CEACAM-6, it is very likely that NEO-201 binds to different types of cancers expressing core 1 and/or extended core 1 O-glycans attached not only to the tumor-associated variant of CEACAM5 and CEACAM6 but also to other protein carriers [17]. The target O-glycosylated protein on neutrophils and other cells in the hematopoietic system is currently under investigation.

The specificity of NEO-201 for a tumor associated antigen suggests a minimal risk of off-target, off-tumor toxicities on surrounding healthy tissue, which has been an important source of toxicity for many mAbs. NEO-201 demonstrated exceptional safety/tolerability in non-human primates, with transient neutropenia, due to high levels of expression of the antigen on mature neutrophils, being the only adverse effect observed [13, 17]. Preclinical in vitro data showed that NEO-201 exerts direct anti-tumor activity through natural killer (NK) cell—mediated ADCC and CDC [13, 14, 18]. In a recent study it has also been demonstrated that NEO-201 mediates ADCC against tumor cells and human neutrophils expressing core 1 or extended core 1 O-glycan profiles [17]. Furthermore, it has also been shown that NEO-201 can block the interaction between CEACAM-5 expressed on tumor cells and CEACAM-1 expressed on NK cells to reverse CEACAM-1-dependent inhibition of NK cytotoxicity [19]. In vivo*,* NEO-201 attenuates growth of human pancreatic tumor xenografts [13] and prolongs survival of ovarian tumor-bearing mice [14].

This study reports results from the first-in-human phase I trial designed to determine the MTD and RP2D of NEO-201 in patients with advanced solid tumors which have progressed on or not responded to standard treatments. Additional correlative data are presented related to NEO-201 pharmacokinetics and pharmacodynamics, specifically, for the effects on immune profile and correlation with treatment toxicity and response.

## Methods

### Study design and patient selection

This is a Phase I first-in-human, single center, open label, dose escalation clinical trial (NCT03476681) using a standard 3 + 3 design. The primary objective was to determine the RP2D of NEO-201. The secondary objective was to assess the preliminary antitumor activity of NEO-201 and exploratory aims characterized the tumor expression of NEO-201 target antigen in archival tumor tissue, pharmacokinetics (PK), immunogenicity and effects of NEO-201 on immunologic correlates, including functional and phenotypic immune response and serum cytokines, chemokines, and soluble factors. All patients in this open-label study were treated at the National Cancer Institute (NCI), National Institutes of Health (NIH), USA. At each dose level, groups of 3–6 patients received NEO-201 intravenously at doses ranging from 1.0 to 2.0 mg/kg every 2 weeks until unacceptable toxicity, patient withdrawal or disease progression. Cycles were 28 days in length.

Dose-limiting toxicities (DLTs) were defined as neutropenic fever, grade 4 neutropenia or thrombocytopenia lasting > 7 days, grade 3 thrombocytopenia with bleeding, or any grade 3 or higher NEO-201–related event occurring during the first cycle. As discussed below, the study was subsequently amended to recognize neutropenia as an expected toxicity, and grade 3 neutropenia improving to grade ≤ 2 with supportive growth factor therapy by the next scheduled dose or grade 3 febrile neutropenia improving within 72 h with or without interventions were not considered DLTs. Other exceptions that were not considered DLTs included transient infusion reactions resolving to grade 1 or better in less than 8 h, and grade 3 anemia less than 2 mg/dL below baseline or resolving to grade 1 or baseline by next scheduled dose. Full DLT definition is provided in the protocol in the supplementary materials. The MTD was defined as the highest dose at which fewer than two of six patients experienced a DLT. At the conclusion of the 2^nd^ cycle, patients that did not experience a DLT and had restaging scans showing SD or clinical response per RECIST v1.1 criteria, were allowed to continue receiving additional cycles of NEO 201 at patient preference and investigator discretion.

Based on previously conducted tissue reactivity studies described above, the dose escalation phase of this trial was opened to tumor types in which reactivity occurred in the majority of samples, including colon cancer, pancreatic cancer, adenocarcinoma of the lung, squamous cell lung cancer, breast cancer, and mucinous and signet cell ovarian cancer. Additional key eligibility criteria included locally advanced or metastatic cancer not eligible for standard therapy known to confer clinical benefit, disease that was measurable by RECIST v1.1 criteria or otherwise evaluable (e.g. bone scan, peritoneal or pleural effusions, carcinomatosis), and adequate performance status (e.g. ECOG ≤ 2 or Karnofsky ≥ 50%). There was no limit on number of prior therapies, including immunotherapies.

### Safety assessments

All patients who received at least two doses of NEO-201 were evaluable for safety and toxicity unless they were removed from study therapy for DLT. Safety evaluations were conducted at every treatment cycle, including determination of adverse events, DLTs during the dose-escalation stage, clinical laboratory measurements, vital signs, and physical examinations. Adverse events were assessed according to the NCI-Common Terminology Criteria for Adverse Events Version 5.0 and were monitored until 30 days after the last dose of study drug.

### Efficacy assessments

Patients were considered evaluable for clinical response if they had measurable disease present at baseline, received at least one cycle of therapy, developed objective disease progression prior to the end of cycle 1, or had their disease re-evaluated by imaging at the conclusion of cycle 1. Radiologic assessment, including CT, MRI, or PET-CT as appropriate, was performed within 28 days prior to initial infusion and was repeated thereafter every 2 cycles. CEA levels and other tumor marker evaluations if appropriate were performed at these time points as well. Response was determined by a blinded central reviewer. Preliminary evidence of efficacy was determined by RECIST v1.1 guideline and reported as Objective Response Rate (ORR) (ORR = complete response (CR), partial response (PR), SD) and Progression Free Survival (PFS).

### Pharmacokinetic (PK) analyses

Blood samples for PK analysis were obtained at pre-dose prior to the first infusion dose on cycle 1 day 1 (C1D1), end-of-infusion (EOI), then at 1 h (hr) post EOI, 4 h post EOI, 24 h post EOI, 72 h post EOI, 7 days post EOI and 14 days post EOI. Spare sampling (pre/trough and end of infusion) was performed on cycle 1 day 15 (C1D15), cycle 2 day 1 (C2D1), cycle 2 day 15 (C2D15), cycle 3 day 1 (C3D1) and samples were used to assess accumulation with biweekly dosing. Assessment into saturable elimination was also made. Blood samples were collected into Serum Separator Tubes (SST®), left to sit at room temperature for 30 min to allow clotting, then centrifuged to obtain serum. The serum was aliquoted into cryovials and frozen at -80 °C until bioanalysis. The first dose (starting from cycle 1, day 1 [C1D1]) exposure metrics of the maximum serum concentration (Cmax), minimum serum concentration (Cmin), area under the serum concentration vs time curve (AUC), half-life, clearance and volume of distribution through dense PK sampling were assessed.

NEO-201 concentration in serum of cancer patients was determined by a fully-validated enzyme-linked immunosorbent assay (ELISA) method with a linear range of 0.25 – 2.0 mg/L (250 – 2000 ng/mL) performed by the NCI Clinical Pharmacology Program. NEO-201 was stable through 2 freeze/thaw cycles in serum at -80 °C, allowing for sample re-analysis. All data on serum NEO-201 concentrations met FDA guidelines for bioanalytical testing.

Specifics of the ELISA and PK analysis are described in Supplementary materials and methods.

### Immunohistochemistry (IHC)

Archival tumor tissue samples were collected from all patients enrolling in the study, but expression of NEO-201 target antigen was not an eligibility criterion. A qualitative IHC staining system was used to identify the expression of the antigen recognized by NEO-201 in formalin-fixed, paraffin-embedded (FFPE) neoplastic tissues from cancer patients. For accuracy and reproducibility in conducting IHC, automated immunohistochemical staining was run and analyzed using the Leica Bond Max Automated Immunohistochemical Staining Procedure (Leica Biosystem, Buffalo Grove, IL, USA). A minimum of 10% of tumor cells in cancer tissues stained at a minimum of 2 + or 3 + intensity of staining with m16C3 reagent antibody (murine version of NEO-201) was considered positive for the expression of the antigen recognized by the clinical NEO-201 mAb. Cancer tissue with 0 or 1 + staining with m16C3 and underlying stromal tissue with 0 intensity of staining with m16C3 were considered negative for the expression of the antigen recognized by NEO-201. In this study, colon carcinoma with 3 + intensity of staining and adjacent colonic mucosa stained at background level of 1 + intensity were used as positive controls for m16C3 antibody in each run.

Additional details about IHC staining procedure are reported in Supplementary materials and methods.

### Correlative assays

#### Cytokines

Blood samples (10 mL) were drawn for cytokine analysis in 10 mL red-top tubes to evaluate the toxicity risk of cytokine release syndrome (CRS). Blood samples were drawn prior to the first infusion dose on C1D1, 24 h post EOI, 72 h post EOI, 14 days post EOI and prior to C3D1. After drawing, blood was allowed to clot at room temperature for a minimum of 2 h and then red-top tubes were centrifuged for 10 min at the 2000 rpm with full brake.

After centrifugation, the serum layer was aliquoted into cryovials designated for immune-monitoring research samples. Cryovials were frozen and stored at -80˚C until assays were performed. Cytokines (IL-1β, IL-2, IL-4, IL-6, IL-8, IL-10, IL-12p70, IL-13, TNF-α, IFN-γ) were evaluated using the V-PLEX Proinflammatory Panel 1 Human Kit (Meso Scale Discovery, Rockville, MD, USA), according to manufacturer’s instructions.

#### Soluble factors

Blood samples (10 mL) were drawn in 10 mL red-top tubes to evaluate the serum levels of soluble CEACAM-5, CEACAM-6 and MICA. Blood samples were drawn prior to the first infusion dose on C1D1, 72 h post EOI, 14 days post EOI and prior to C3D1. After drawing, blood was processed with the same procedure described for cytokines analysis. The day of the assay, an aliquot of serum for each time point was defrosted and used at 1:3 dilution. Serum levels of soluble factors were detected through ELISA using the following kits and following manufacturer’s instructions: soluble CEACAM-5: Human Carcinoembryonic Antigen ELISA Kit (Abcam, Cambridge, MA, USA); soluble CEACAM-6: Human CD66c / CEACAM6 (Sandwich ELISA) ELISA Kit (LSBio, Seattle, WA, USA); soluble MICA: MICA Human ELISA Kit (Thermo Fisher Scientific, Waltham, MA, USA).

#### NK cells and regulatory T cells (Tregs) phenotype analysis

Analysis of the expression of cell-surface and intracellular proteins in peripheral blood mononuclear cells (PBMCs) from cancer patients was performed by flow cytometry to evaluate NK cells and Tregs phenotype. PBMCs from cancer patients were utilized under the appropriate NCI Institutional Review Board approval (protocol code NCT03476681, first approved 03/26/2018; latest update 01/08/2020). To isolate PBMCs, blood from cancer patients was collected in Heparin Green Top tubes.

For NK phenotype analysis, blood samples were drawn prior to the first infusion dose on C1D1, 72 h post EOI, 14 days post EOI and prior to C3D1. For Tregs phenotype analysis blood samples were drawn prior to the first infusion dose on C1D1, 14 days post EOI and prior to C3D1. Isolated PBMCs were cryopreserved in cryovials containing 95% human AB serum + 10% DMSO in liquid nitrogen. The day of the flow cytometry PBMCs were thawed and stained with primary anti-human mAbs in 1X PBS + 1% BSA (Teknova, Hollister, CA, USA) for 30 min at 4 °C.

To detect the NK surface markers PBMCs were labeled with following antibodies: CD56 PE (clone 5.1H11), CD16 PerCP-Cy5.5 (clone 3G8), NKG2D BV421 (clone 1D11), NKp46 FITC (clone 9E2) (BioLegend, San Diego, CA, USA), CD107a APC-H7 (clone H4A3) (BD Biosciences, San Jose, CA, USA), CEACAM-1 APC (clone 283340) (VWR, Radnor, PA, USA). To detect surface Tregs markers and to evaluate the reactivity of NEO-201 to human Tregs, PBMCs were labeled with the following anti-human mAbs: CD4 FITC (clone OKT4), CD127 APC (clone A019D5), CD15s PE (clone FH6), NEO-201 Pacific Blue (BioLegend, San Diego, CA, USA), CD25 APC-H7 (clone M-A251) (BD Biosciences, San Jose, CA, USA).

For Tregs analysis, after staining surface markers, cells were permeabilized with Fix/Perm Solution (eBioscience™ Foxp3 / Transcription Factor Staining Buffer Set, Thermo Fisher Scientific) for 1 h at 4 °C to allow detection of intracellular transcription factors, and then stained in 100 uL of 1X Permeabilization Buffer for 1 h at room temperature in the dark with 2-4µL/sample of the anti-human Foxp3 PerCP-Cy5.5 mAb (clone 236A/E7, BD Biosciences, San Jose, CA, USA).

After staining, cells were washed twice with cold 1X PBS and examined using a FACSVerse flow cytometer (BD Biosciences, San Jose, CA, USA). Analysis of cellular fluorescence was performed using BD FACSuite software (BD Biosciences, San Jose, CA, USA) and FlowJo 10.8.1. Positivity was determined by using fluorescence-minus-one controls.

Complete staining procedure is reported in Supplementary materials and methods.

## Statistical analysis

Student’s t-test, 1-way ANOVA, and 2-way ANOVA with Bonferroni post-test analysis were performed where indicated. The number of samples chosen for each comparison was determined based on past similar experiments or by performing pilot experiments to assess the expected magnitude of differences. The number of experiments performed is indicated in the figure legends. Biological assays were performed in triplicate, with separate donors before statistical analysis was performed.

## Results

### Study population and disposition

Between January 8, 2019 and December 8, 2020, a total of 17 patients received one or more doses of NEO-201. The demographics of patients enrolled are listed in Table 1. Among the 17 patients enrolled, 11 had colorectal cancer, 4 pancreatic cancer and 2 breast cancer (both ER^+^/PR^−^/HER2^−^). The median age of the patient population was 60 (range 37–85). All patients had metastatic disease and had received and progressed on frontline therapy (median number of prior therapies 4.5, range 1–10).

**Table 1:** Patient demographics and baseline characteristics

(NEO-201)		
Variable	**Trait**	**Number**
Total	Patients	17
Gender	Female	11
	Male	6
Race	White	15
	African American	2
	Native Hawaiian or other	0
	Pacific Islander	0
	Other	0
Ethnicity	Hispanic	0
	Non-Hispanic	17
Age	18 – 30	0
	31 – 40	1
	41 – 50	4
	51 – 60	4
	61 – 70	5
	71 – 80	2
	> 80	1
Disease Histology	Adenocarcinoma of pancreas	4
	Colorectal cancer	11
	Breast cancer	2

Prior to treatment with NEO-201, ten and six patients with colorectal cancer had received anti-VEGF and anti-EGFR directed therapy respectively, and one patient had previously received an immune checkpoint inhibitor (pembrolizumab). Almost all (10/11) patients with colorectal cancer were microsatellite stable. Of the patients with pancreatic cancer, two had received immune checkpoint inhibitors (one pembrolizumab and one ipilimumab + nivolumab) prior to receiving NEO-201. Both patients with breast cancer were treated with anti-estrogen therapy (fulvestrant) and one with ipilimumab + nivolumab prior to receiving NEO-201. One patient with pancreatic cancer and four patients with colorectal cancer showed mutations in the KRAS gene. TMB status was known in only 4 patients: one patient with pancreatic cancer, one patient with breast cancer and one patient with colorectal cancer had intermediate TMB status, while one patient with colorectal cancer showed low TMB status. All patients tested (one with colorectal, one with breast, one with pancreatic cancer) were negative for PDL-1. A panel of all gene alterations recorded for each patient is provided in Supplementary Table 1.

### Toxicity

Among the 17 patients enrolled, 4 patients received NEO-201 at DL 1 (1 mg/kg), 7 patients received NEO-201 at DL 2 (2 mg/kg) and 6 patients received NEO-201 at DL 1.5 (1.5 mg/kg) (Table 2). Two patients withdrew consent before the DLT evaluation period had been completed and were evaluated for adverse events but not for DLT. Twelve patients discontinued NEO-201 due to disease progression, and 3 patients discontinued due to DLTs as further described below.

**Table 2:** Most common grade 3 (Gr3) and grade 4 (Gr4) adverse events (AEs) in all patients

	**Dose Level 1** **1 mg/kg** *n* = number of events in 4 patients/ 12 doses		**Dose Level 2** **2 mg/kg** *n* = number of events in 7 patients/ 34 doses		**Dose Level 1.5** **1.5 mg/kg** *n* = number of events in 6 patients/ 23 doses		**Cumulative Incidence** *n* = number of events in 17 patients/ 69 doses (%)
**Adverse Event**	**Gr 3**	**Gr 4**	**Gr 3**	**Gr 4**	**Gr 3**	**Gr 4**	
Anemia			2				2 (3%)
Febrile Neutropenia			2	1	1		4 (6%)
Sepsis				1			1 (0%)
Lymphocyte count decreased	1		4	2		1	8 (12%)
Neutrophil count decreased	2	4	2	10	1	7	26 (38%)
White blood cell decreased	2		7	2	1	4	16 (23%)
Hypertension	1						1 (0%)

See Supplementary Table 2 for report of all AEs

Due to the ability of NEO-201 to kill neutrophils expressing core 1 O-glycans through ADCC [17], we noted grade 3/4 transient neutropenia in all patients (26/69 doses, 17/17 patients) as a common on-target, off-tumor effect of NEO-201. Common (> 10%) grade 3/4 toxicities, as reported in Table 2, included white blood cell count decreased (16/69 doses, 12/17 patients) and lymphocyte count decreased (8/69 doses, 6/17 patients). Less common (< 10%) grade 3/4 toxicities, as reported in Table 2, were febrile neutropenia (4/69 doses, 4/17 patients), anemia (2/69 doses, 2/17 patients), sepsis (1/69 doses, 1/17 patients) and hypertension (1/69 doses, 1/17 patients) (Table 2 and Supplementary Table 2).

Three patients received NEO-201 at DL 1 for two doses without experiencing a DLT. After completion of 2 doses in three patients at DL 2, one patient experienced a DLT of prolonged neutropenia. DL 2 was subsequently expanded to 6 patients (1 patient withdrew consent after the first dose and was therefore not evaluable for DLT and was replaced in the enrollment). A second patient at DL 2 developed grade 4 febrile neutropenia (DLT). On further review of baseline imaging, this patient was noted to have necrotic tumor in close proximity to bowel, that was considered high risk for infection.

After completion of the first 3 patients in DL 2, the protocol was amended to recognize severe neutropenia as an expected toxicity and to allow for administration of filgrastim to shorten the duration of the neutropenia and to reduce the risk of infection. Patients who developed neutrophil decrease after NEO-201 infusion were treated with filgrastim until the absolute neutrophil count (ANC) exceeded 1000/mm^3^. At the completion of 6 patients at DL 2, additional changes were made to the protocol to include increased screening for infection risk factors and exclusion of patients with unacceptable risk of infection. In addition, an interim DL 1.5 was introduced to mitigate the risk observed at DL 2. DL 1.5 was determined to be the MTD after six patients were treated with only one DLT (grade 3 febrile neutropenia). Based on these safety data and pharmacokinetics, DL 1.5 was also determined to be the RP2D. Six patients received 23 doses of NEO-201 at 1.5 mg/kg/dose and the grade 3 and 4 toxicities included neutropenia (8/23 doses), decreased WBC (5/23 doses), and lymphopenia (1/23 doses) (Table 2).

### Activity

**Fig. 1:**
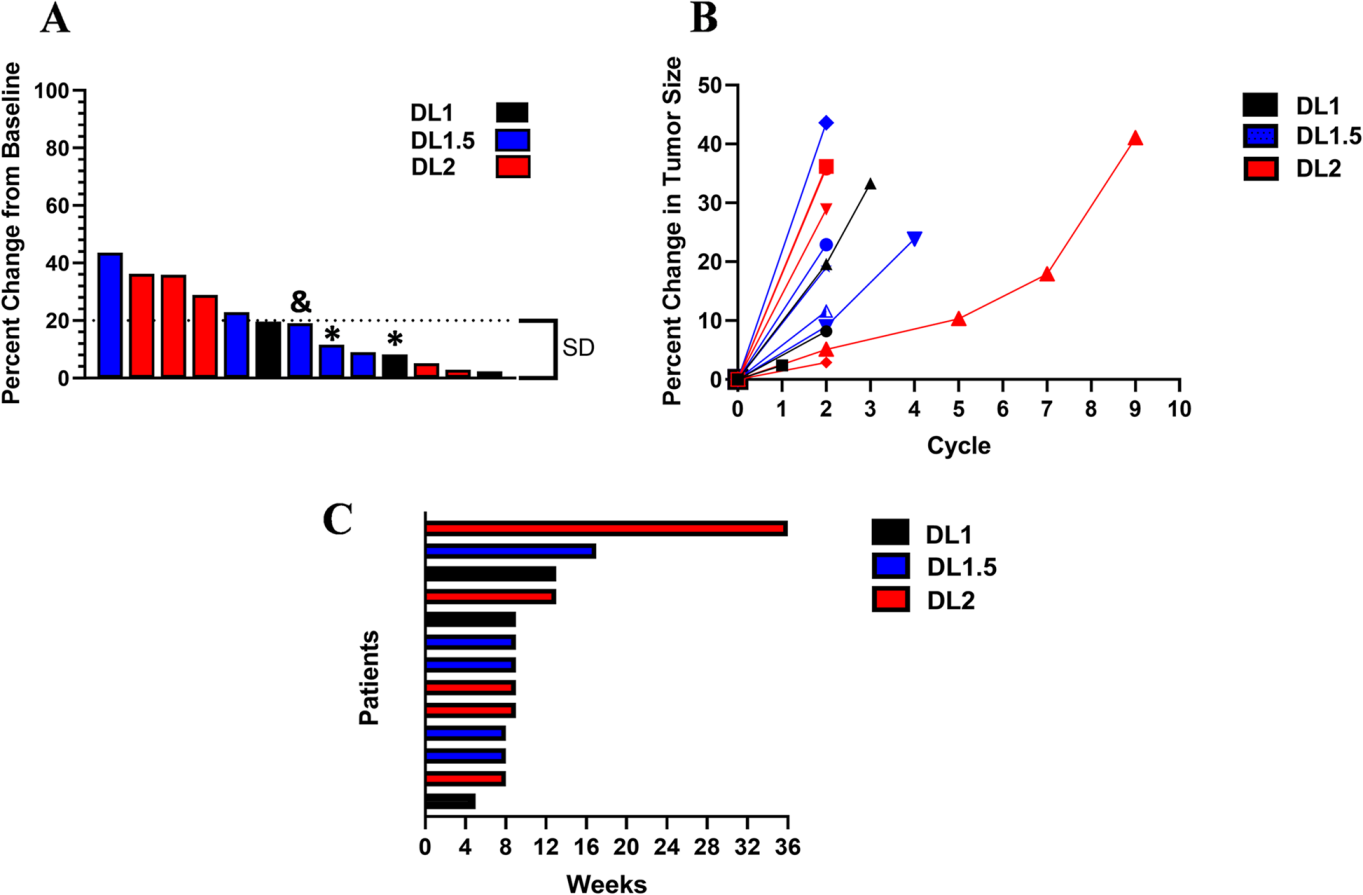
RECIST response and time on study **A**. Waterfall plot indicating best response and best percent change in tumor size for all patients eligible for response evaluation ( *n* = 13). Dose level (DL) is indicated as in the legend and the dotted line at 20% indicates the threshold for stable disease (SD) according to RECIST v1.1 criteria. Two patients (represented by *) showed new liver lesions at the restaging performed at the end of cycle 2 and were considered as progressive disease according to RECIST v1.1 criteria. One patient (represented by &), although did not show more than 20% increase in the target lesions, was deemed to have clinical progressive disease due to worsening pain, bloating, and ascites recorded at the clinical visit after restaging performed at the end of cycle 2. **B**. Spider plot of percent change in tumor size across all cycles. Each cycle is 28 days in length (4 weeks). Among patients with SD, three patients were restaged after cycle 2. One patient was restaged multiple times after cycle 2 until the end of cycle 9 (36 weeks of treatment)** C.** Swimmer plot of time on study by dose level for all evaluable patients. All 4 patients with SD after at least 4 doses of NEO-201 (8 weeks of treatment) elected to continue receiving therapy

Thirteen patients were able to undergo assessment for disease response. The best response observed was SD (> 56 days) in 4 patients, all of whom had colorectal cancer (Fig. 1A). No PR or CR was observed. The range of best tumor size change in the patients with SD was + 2.4 to + 19.6% (Fig. 1B). CEA, CA-19–9 and/or additional applicable tumor markers were measured as an exploratory objective. No CEA responses were observed. Minor CA-19–9 reductions were observed in two patients with pancreatic cancer at DL 1.5, from 882.8 to 802.9 and from 113 to 84.9 U/mL, respectively.

All 4 patients with SD after at least 4 doses of NEO-201 elected to continue receiving therapy, range of 1–11 additional doses (Fig. 1C). The longest subject on study was a patient with colorectal cancer at DL 2 who received 15 doses and was able to remain on treatment for 9 cycles (36 weeks) before discontinuing treatment due to clinical progression. All patients with SD had mutations in RAS genes. Three patients harbored KRAS gene mutations, one patient had mutation in NRAS gene, and one patient showed both KRAS and NRAS mutated (Supplementary Table 1).

### Pharmacokinetic analyses

**Fig. 2:**
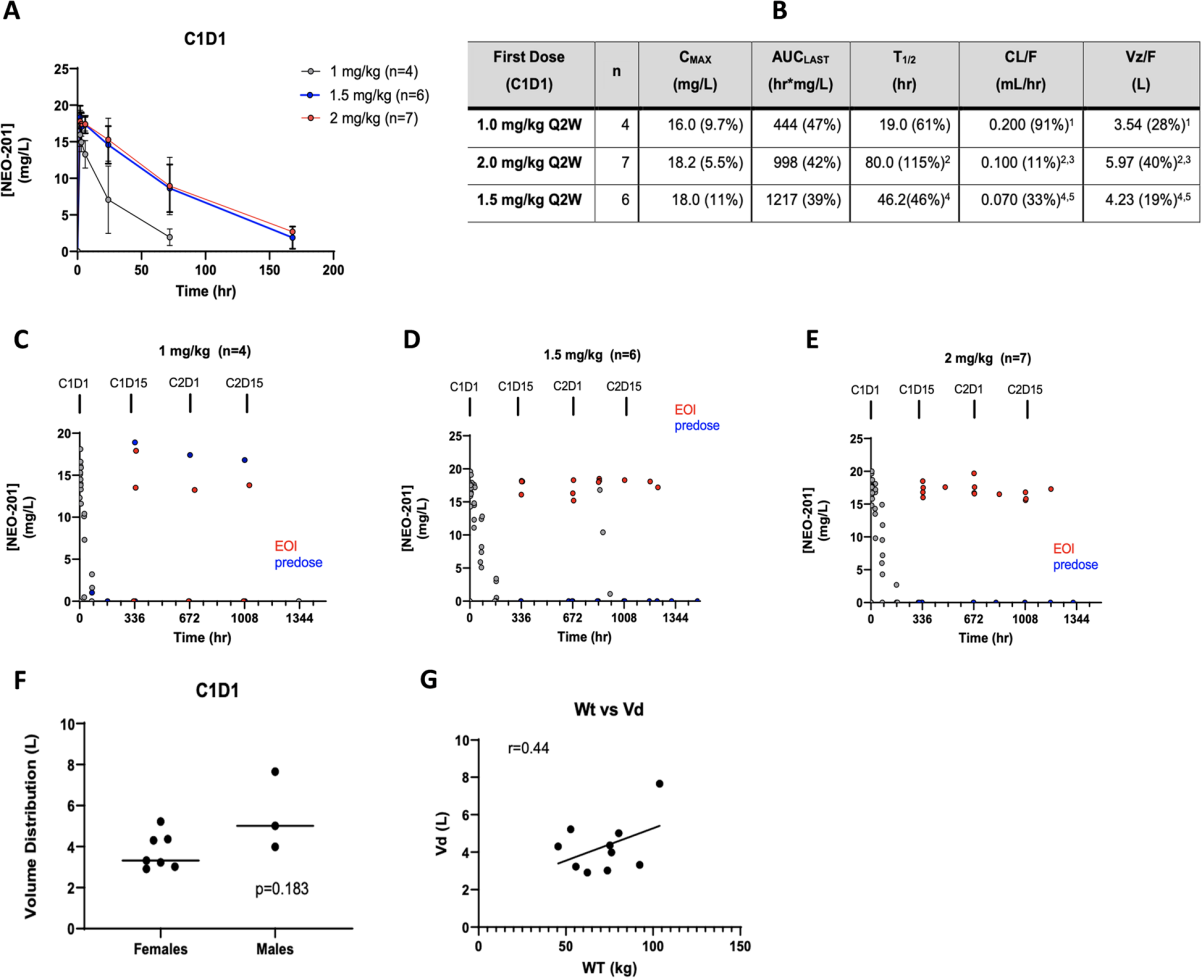
Clinical Pharmacokinetics of NEO-201 **A**. NEO-201 serum concentrations averaged for each dose level and plotted over time. **B.** Noncompartmental analysis of first dose of NEO-201 including the mean estimates for relevant PK parameters during dense PK sampling following the first dose given on C1D1. *Data presented as arithmetic means (%CV) due to low numbers in each group ^1^ Two patients had inaccurate estimates of C1D1 clearance and volume due to an overestimation of AUCinf. ^2^ One patient had insufficient terminal data to accurately estimate a half-life, thus clearance and volume too. ^3^ Four patients had inaccurate estimates of C1D1 clearance and volume due to an overestimation of AUCinf. ^4^ One patient had insufficient terminal data to accurately estimate a half-life, thus clearance and volume too. ^5^ Two patients had inaccurate estimates of C1D1 clearance and volume due to an overestimation of AUCinf. **C.** Individual NEO-201 serum concentration–time profiles of 1.0 mg/kg cohort. **D-E.** Individual NEO-201 serum concentration–time profiles of patients in 1.5 mg/kg cohort (**D**) and 2.0 mg/kg cohort (**E**). **F-G.** Differences in NEO-201 distribution volume by sex (**F**) and body size (**G**)

All 17 patients were evaluable for first dose noncompartmental pharmacokinetic analysis. First dose NEO-201 serum concentration–time profiles were measured for all 17 patients across the three different dose levels (Fig. 2A). Intravenous infusions of NEO-201 demonstrated a mono-exponential disposition following cessation of drug delivery, and serum concentrations were below quantifiable assay limits by the time of the next dose on C1D15. There were dose-proportional increases in both C_MAX_ and AUC_LAST_. First dose serum concentrations were averaged for each dose level and plotted over time, where a dose-dependent change in half-life becomes evident (Fig. 2B). This is a common feature of humanized monoclonal antibodies, as exposure increases with higher doses, saturation of target binding occurs, which is the predominant route of clearance for human mAbs.

Relevant PK parameters during dense PK sampling were calculated following the first dose given on C1D1 (Fig. 2B). In the 1.5 mg/kg cohort we observed that in 4 of 7 patients, NEO-201 was still detectable in the serum 7 days post first infusion and was likely cleared completely between 6- and 8-days post-infusion. Regardless of the longer half-life and apparent slower clearance rates at higher doses (Fig. 2B), there was no evidence of drug accumulation at any dose level on the investigated twice-weekly dosing schedule (Fig. 2C, D, E). Given the negligible change in EOI peak concentrations in cycles 2 or later compared to first dose, it is unlikely that anti-drug antibodies are significantly impacting NEO-201 drug exposure. Based on the dense sampling of first dose kinetics in these 17 patients, the distribution volume is consistent over the observed dose levels and is comparable with that of typical monoclonal antibodies, which generally have a total distribution volume of 8–20 L in a typical 70-kg adult [20]. While males had a larger mean drug distribution volume (5.55 L vs 3.77 L); this was not statistically significant (* p* = 0.183; Fig. 2F). This apparent sex effect is most likely due to body size, as evident from a significant correlation (*r* = 0.44) with body weight (Fig. 2G).

### Time of administration of filgrastim affects NEO-201 PK

Ten of 17 patients (4 in DL 2 and 6 in DL 1.5) received filgrastim to shorten the duration of the neutropenia.

In the DL 2 cohort, time of administration of filgrastim to mitigate neutropenia had an impact on NEO-201 concentration in the serum of some patients (Supplementary Fig. 1A). As an example, patient 7 received filgrastim on C1D8 (168 h after infusion), when ANC value was 0.02 and when NEO-201 had declined to low levels (2.7 ug/mL) in the serum. The patient recovered completely from neutropenia by day 14 after infusion. Similar pattern has been observed in patient 11. This patient received filgrastim 72 h after infusion, when ANC value was 0.23 and when NEO-201 had declined to less than 0.25 ug/mL in the serum. The patient recovered completely from neutropenia by day 7 after infusion. Recovery from neutropenia in these two patients was likely aided by the declining levels of NEO-201 at that time, allowing less killing of neutrophils released from the stimulated bone marrow by declining levels of NEO-201 and thus faster recovery from neutropenia. Conversely, patient 9 and 10 received filgrastim 24 h after NEO-201 infusion. In these patients, NEO-201 levels declined more rapidly than patient 7, and NEO-201 was undetectable in serum by day 7 after infusion. Binding of NEO-201 to neutrophils released from the stimulated marrow likely resulted in a decrease in NEO-201 serum concentration compared to patient 7. These data suggest that the levels of neutrophils and NEO-201 are reciprocal, and that administration of filgrastim too early in the cycle could decrease NEO-201 serum levels. Based on this observation, almost all patients (5/6) in the DL 1.5 cohort received filgrastim starting on day 5 or day 6 of the NEO-201 treatment. Hence, NEO-201 was still detectable by day 7 after infusion in the majority of patients in the DL 1.5 cohort (Supplementary Fig. 1B), and all patients that received filgrastim on day 5 or day 6 recovered from neutropenia within 7–10 days from initiation of filgrastim. These data suggest that the best time to begin administering filgrastim to mitigate neutropenia without affecting NEO-201 PK should be 5–6 days after NEO-201 infusion.

### Pharmacodynamic analyses

#### Immunohistochemistry (IHC)

Baseline tissue samples from all patients enrolled in the study were tested for NEO-201 expression by IHC. Fifteen of 17 patients had tissue evaluable for IHC, and of these 14/15 had more than 90% tissue stain positive for the NEO-201 antigen with 3 + intensity. One breast cancer patient had 3 + staining in about 20% of the tumor section analyzed (Supplementary Table 3). Representative IHC staining from 6 colon, 2 breast and 2 pancreas tumor tissues is shown in Supplementary Fig. 2. These data are consistent with previously published IHC testing during preclinical characterization of NEO-201 [13–15].

#### Cytokine analysis

**Fig. 3:**
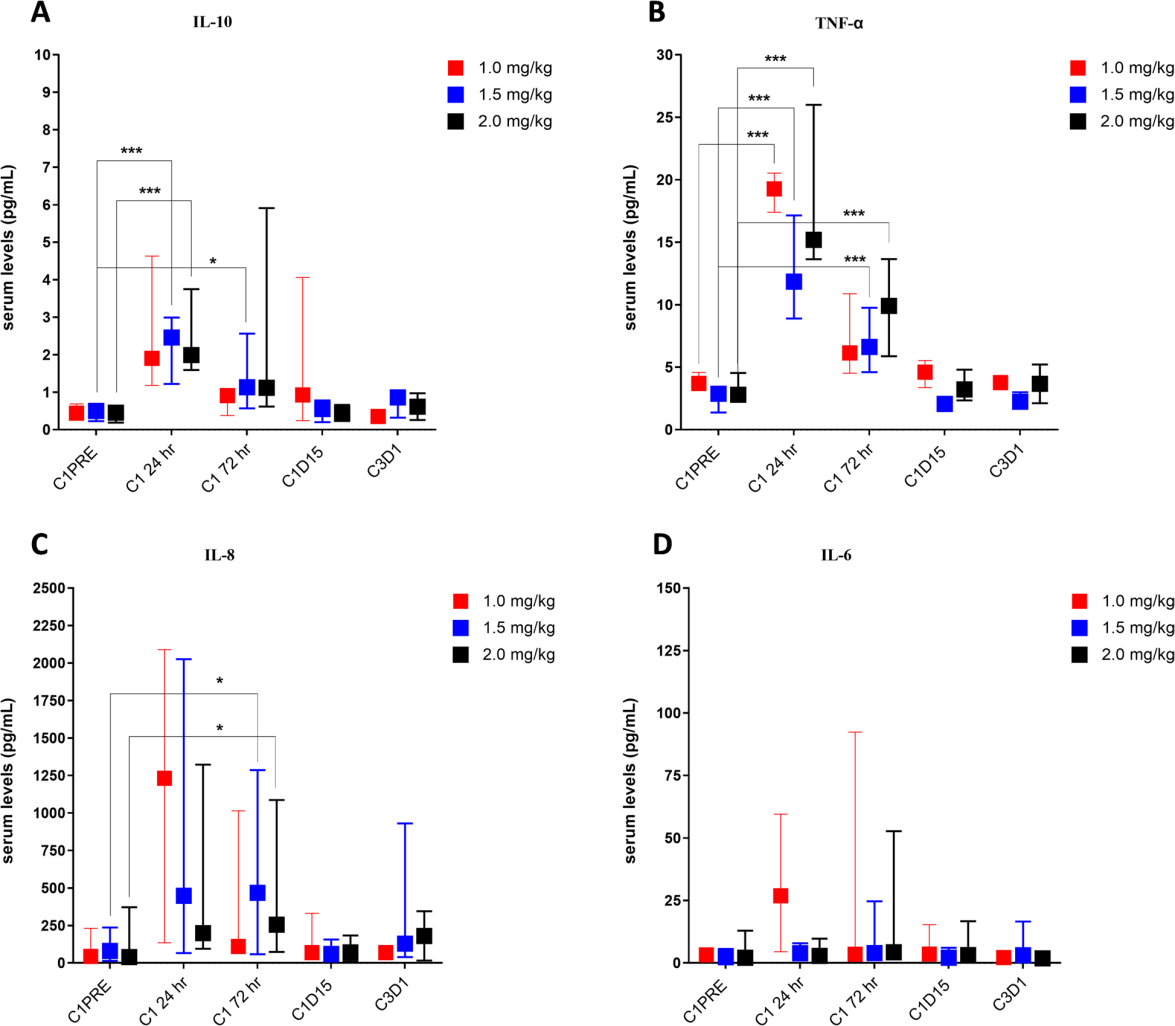
Serum cytokines modulation after NEO-201 infusion. Serum cytokines were evaluated using the V-PLEX Proinflammatory Panel 1 Human Kit. The figure depicts modulation of serum levels of cytokines statistically significant after NEO-201 treatments at different time points compared to baseline levels before treatment (C1PRE). **A.** Comparison of IL-10 median serum levels prior and post treatment. **B.** Comparison of TNF-α median serum levels prior and post treatment. **C.** comparison of IL-8 median serum levels prior and post treatment. **D.** comparison of IL-6 median serum levels prior and post treatment. * statistically significant (*p* < 0.05) by 2way ANOVA; ** statistically significant (*p* < 0.01) by 2way ANOVA; *** statistically significant (*p* < 0.001) by 2way ANOVA

To evaluate modulation of cytokines by NEO-201, serum samples were collected at timepoints before and after treatment and analyzed by ELISA-based cytokine array. At 24 h from infusion, serum IL-10 and TNF-α levels were increased in all patients at all dose levels. IL-10 increased to median fourfold higher in all patients compared to pre-infusion, and TNF-α to median 4- to sixfold higher in all patients (Fig. 3A, 3B). IL-10 and TNF-α levels had begun to decrease toward baseline by 72 h from infusion in all patients. Median serum IL-8 levels were fivefold higher after 24 and 72 h from infusion in patients from 1.5 mg/kg cohort compared to the baseline levels before infusion (Fig. 3C). In 6 patients (2 from 1.0 mg/kg, 1 from 1.5 mg/kg and 3 from 2.0 mg/kg cohort) serum IL-8 levels remained elevated until C1D15.

No change in IL-12p70, IL-13, IL-1β, IL-2, IL-4 levels were observed in these patients after NEO-201infusion at all time points in all cohorts (data not shown). Effects on IL-6 were variable, with elevated median serum IL-6 levels detected in 3 patients (2 from 1.0 mg/kg, 1 from 2.0 mg/kg) at 24 h post-infusion. IL-6 levels returned to baseline levels at 72 h post-infusion, except for in one patient with pancreatic cancer in the 2.0 mg/kg cohort in which IL-6 levels remained elevated until C1D15 (Fig. 3D). A slight increase of serum IFNγ levels was observed 24 h after infusion only in two patients from the 2.0 mg/kg cohort (data not shown).

### Soluble MICA affects NK cell phenotype

**Fig. 4:**
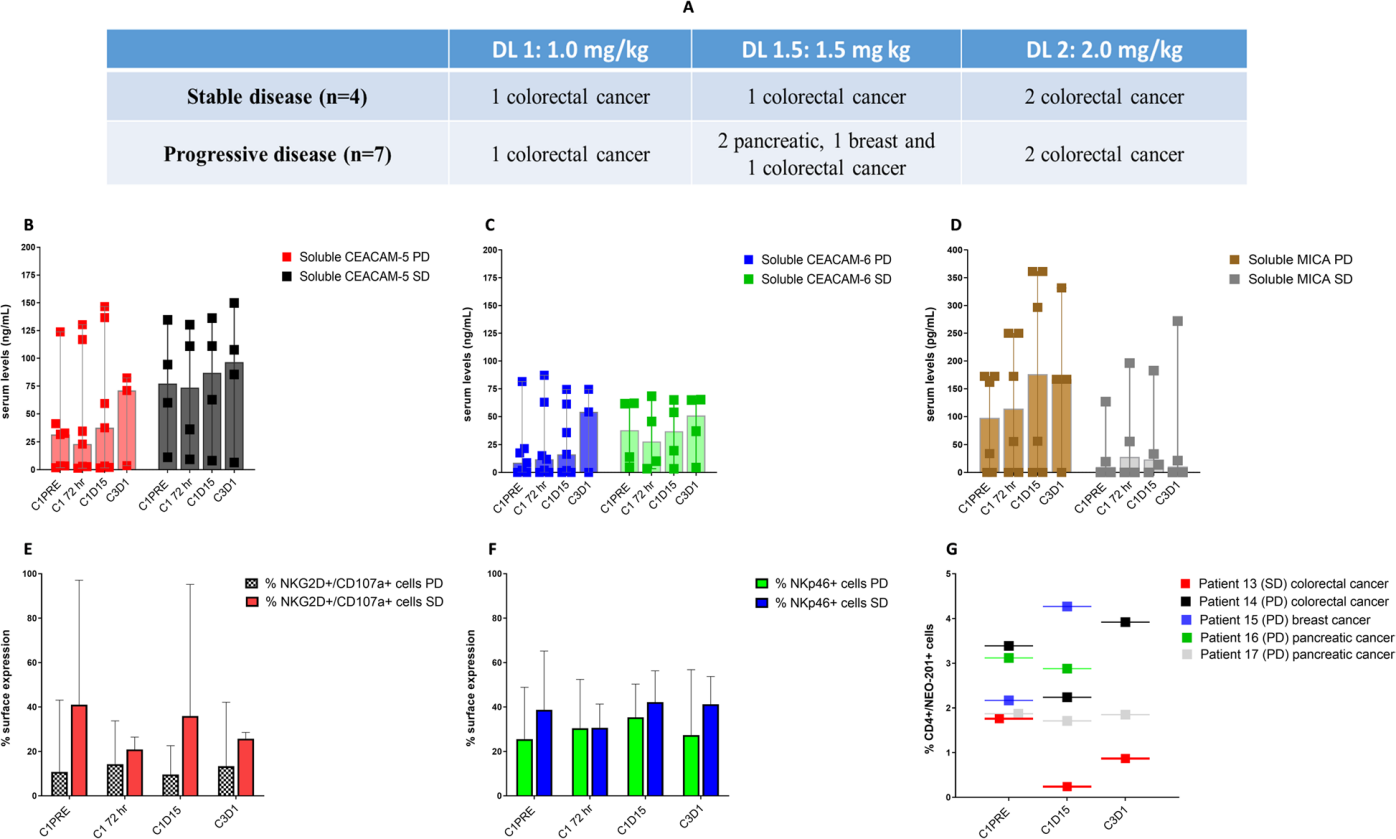
Correlation between soluble factors and immune cells anti-cancer activity **A**. Clinical characteristics and treatment response of the 11 patients evaluated divided by dose level. Comparison of median serum levels of soluble CEACAM-5 (**B**), CEACAM-6 (**C**) and MICA (**D**) in patients with stable (SD) and progressive disease (PD) at different time points. Serum levels of soluble factors were detected using commercially available ELISA kits. Statistically significant difference between groups was determined by 2way ANOVA. Differences in median serum levels of soluble CEACAM-5, CEACAM-6, and MICA in patients with SD compared to patients with PD were not statistically significant. **E–F**. Comparison of the percentage of NKG2D^+^/CD107a^+^ and NKp46^+^ NK cells in patients with stable (SD) and progressive disease (SD) at different time points by flow cytometry analysis. NKG2D^+^/CD107a^+^ and NKp46^+^ NK cells were gated from CD56^+^/CD16^+^ population from PBMCs. Data are presented as median of percentage of viable cells expressing NK markers. Positivity was determined by using fluorescence-minus-one controls. **G.** Comparison of the percentage of circulating CD4^+^/NEO-201^+^ Tregs at different time points in patients treated with NEO-201 DL 1.5 by flow cytometry analysis. CD4^+^/NEO-201^+^ population was gated from PBMCs. Data are presented as percentage of viable cells. Positivity was determined by using fluorescence-minus-one controls

The release of soluble factors from cancer cells constitutes an immune escape mechanism that systemically impairs efficacy of immunotherapy [21]. Elevated serum levels of soluble MICA and soluble CEACAM-5 and CEACAM-6 have been correlated with impairment of NK cell activity, cancer progression and metastasis [22–24]. Since NEO-201 uses NK cells as one of the main effectors to kill its target cells through ADCC, we evaluated the relationship of soluble CEACAM-5, CEACAM-6 and MICA with the activation markers of NK cells from cancer patients enrolled in this study, and we measured the correlation between serum levels of soluble factors and response to treatment (PD or SD). Serum levels of soluble factors were evaluated by ELISA, and NK cell activation status was evaluated in 11 patients through flow cytometry (Fig. 4). Six patients were not evaluable because serum samples or PBMCs were not collected at the designated time points. Before the first NEO-201 infusion and at 72 h, C1D15 and C3D1 time points, median serum levels of soluble CEACAM-5 and CEACAM-6 tended to be higher in patients with SD compared to patients with PD at all time points, but this difference was not statistically significant (Fig. 4B and 4C). Conversely, median serum levels of soluble MICA prior to treatment with NEO-201 were tenfold higher in patients with PD compared to patients with SD (C1 PRE: 97.76 vs 9.71 pg/mL) (Fig. 4D). Serum levels of soluble MICA increased at C1D15 and C3D1 in patients with PD, although the trend was not statistically significant due to small numbers. In contrast, the median serum levels of soluble MICA remained stable at all time points in patients with SD (Fig. 4D).

Since it has been demonstrated that tumor-derived soluble MICA can negatively impact NK cell cytotoxicity by modulating the NKG2D pathway [25, 26], we investigated if high serum levels of soluble MICA in patients with PD was correlated with a downregulation of NK activation and cytotoxic markers. Expression of NKG2D^+^/CD107a^+^, NKp46^+^ and CEACAM1^+^ cells was measured by flow cytometry on circulating CD56^+^/CD16^+^ NK cells before and after treatment. The median percentage of NKG2D^+^/CD107a^+^ and NKp46^+^ NK cells trended toward a lower baseline value in patients with PD than in patients with SD (NKG2D^+^/CD107a^+^: 10.8% vs 41.1%; NKp46^+^: 25.6% vs 38.8%) and remained lower at all time points (Fig. 4E and 4F). No difference in expression of CEACAM1 was observed between patients with SD and PD (data not shown). Altogether, these data suggest that high serum MICA levels in patients with PD could be correlated with downregulation of NK activation and cytotoxic markers, impairing the NK-mediated ADCC triggered by NEO-201 against tumor cells.

### NEO-201 binds to circulating Tregs

In a previous study, flow cytometry analysis of hematopoietic cells for NEO-201 binding revealed that approximately 4.6% of CD4^+^ T cells were positive for NEO-201 staining [19]. To investigate if this small fraction of CD4^+^ cells constitutes the Treg population, PBMCs from 5 patients in the 1.5 mg/kg cohort were profiled by flow cytometry for expression of specific Treg markers, including CD4, CD25, CD127, Foxp3, CD15s. We queried whether cells expressing those Tregs markers were also NEO-201^+^. Flow cytometry analysis of PBMCs obtained prior to initiation of treatment with NEO-201 revealed that the majority of CD4^+^/NEO-201^+^ cells are also CD25^+^/CD127^−^/Foxp3^+^, an immunophenotype consistent with that of Tregs (Supplementary Fig. 3).

One marker used to characterize a subset of highly immunosuppressive Tregs is CD15s [27]. Accordingly, we measured the percentage of Foxp3^+^/CD15s^+^ cells within the CD4^+^/NEO-201^+^/CD25^+^/CD127^−^ Treg population to assess if NEO-201 can identify this subset of highly differentiated and suppressive Tregs in human PBMCs. Our data show that Tregs recognized by NEO-201 express a high percentage of Foxp3^+^/CD15s^+^ cells (average 66.14%), suggesting that NEO-201 can recognize highly suppressive Tregs in the peripheral blood of cancer patients (Supplementary Fig. 3). To evaluate if treatment with NEO-201 affected the percentage of NEO-201 positive circulating Tregs, we analyzed the same Treg markers at the C1D15 and C3D1 time points. One patient with SD showed a reduction of 86.4% and then 50.6% of circulating CD4^+^/NEO-201^+^ Tregs at C1D15 and C3D1 respectively, compared to baseline levels (C1D15: 0.24% vs 1.76%; C3D1: 0.87% vs 1.76%) (Fig. 4G). Conversely, no reduction of circulating CD4^+^/NEO-201^+^ Tregs was observed at these timepoints in the other 4 patients, all of whom had PD, except for one subject with colorectal cancer that showed a transient reduction of 33.93% at C1D15 vs baseline (2.24% vs 3.39%) (Fig. 4G).

## Discussion

One of the mechanisms involved in cancer development, progression and metastasis is the disruption of post-translational modifications of proteins and lipids, such as glycosylation. One glycosylation pattern altered in cancer cells is the O-glycosylation. During oncogenesis, truncated O-glycans can be expressed. This occurs when N-acetyl galactosamine (O-GalNAc) is added to the amino acids serine and threonine on cancer cells’ carrier proteins [28]. This modification is important in regulating many biological processes. The expression of incomplete/truncated O-glycans has been correlated with poor prognosis and tumor progression in solid tumors of epithelial origin such as breast, ovarian, gastric, pancreatic, colon cancers, and in hematologic neoplasms such as AML and multiple myeloma (MM) [29–33]. One promising strategy to improve cancer immunotherapy efficacy could be the employment of monoclonal antibodies that specifically target truncated O-glycans expressed in cancer cells. In this regard, NEO-201 can represent a novel promising mAb candidate for the treatment of cancers expressing O-glycans.

In contrast to many antitumor antibodies, which were developed against a normal human protein, NEO-201 was raised against immunogenic tumor extracts as part of an allogenic colorectal cancer vaccine program [34]. In previous studies, we reported that NEO-201 binds specifically to different cancers, such as colon, ovarian, pancreatic, non-small cell lung, head and neck, cervical, uterine and breast but does not react to normal epithelial tissues [13–15]. NEO-201 can also bind to human neutrophils and AML and MM cell lines in vitro [19, 35].

A recent study showed that NEO-201 recognizes specifically core 1 and/or extended core 1 O-glycans, and that NEO-201 binds and kills target cells expressing core 1 and/or extended core 1 O-glycans, through ADCC [17]. In addition to ADCC, NEO-201 has different mechanisms of action to kill cancer cells expressing its target antigen, including CDC and the blockade of the CEACAM5/CEACAM1 immune checkpoint inhibitory pathway [15].

This manuscript reports the results of the first-in-human, phase I dose escalation study of anti-core 1 O-glycans mAb NEO-201 in patients with solid tumors. Pre-clinical toxicity studies performed in cynomolgus monkeys showed exceptional safety/tolerability of NEO-201, with a transient decrease in neutrophils being the only adverse effect observed [13]. Predominant toxicities in humans resulted from the known on-target, off-tumor effect of NEO-201 of binding to and depleting neutrophils through ADCC [13, 17]. Binding of NEO-201 to the neutrophils, and their subsequent killing by ADCC, resulted in their rapid depletion in peripheral blood and some degree of neutropenia for the duration that NEO-201 remained detectable in serum. Neutropenia ranged from 8 to 14 days after infusion, depending on dose level and timing of administration of filgrastim. NEO-201 levels and neutrophil levels resulting from filgrastim administration displayed a reciprocal relation. It is likely that the high levels of neutrophils released into the bloodstream by filgrastim administration soon after NEO-201 administration act as a “sink”, binding NEO-201 and removing it from the bloodstream. In balancing the infection risks of prolonged neutropenia vs. potentially reducing the amount of free NEO-201 able to bind to tumor, we determined that administration of filgrastim beginning 5–6 days after NEO-201 administration was optimal for improvement in neutropenia without prematurely diminishing NEO-201 levels in serum.

Aside from the expected complications of neutropenia, NEO-201 was otherwise well tolerated. As expected from the binding profile of NEO-201 in animal studies and in vitro [15], we did not observe toxicities attributable to off-target binding of stromal tissues. After the amendment of the protocol and allowance of filgrastim administration, a single patient (out of 6) at DL 1.5 experienced grade 3 febrile neutropenia, and this dose was determined to be the MTD and the RP2D. Mild (grade 1–2) infusion reactions were relatively frequent but did not result in treatment discontinuation in any patient. As a measure of tolerability, all 4 patients with SD after at least 4 doses of NEO-201 elected to continue receiving therapy, and one patient with colorectal cancer at DL 2 was able to remain on treatment for 9 cycles (36 weeks) before clinical progression.

Correlative studies revealed that NEO-201 infusion resulted in an upregulation of serum IL-10 and TNF-α levels, with more variable effects on other proinflammatory cytokines such as IL-8 and IFNγ. These cytokines are all classically considered proinflammatory and are elevated during acute inflammation. If inflammation becomes chronic, IL-10 and TNF-α could also have effects that block antitumor immunity by promoting the expansion of immunosuppressive cells such as Tregs and myeloid-derived suppressor cells (MDSCs) [36, 37]. MDSCs, especially in the tumor microenvironment (TME), can result in NK cells exhaustion, by suppressing IL-2 mediated NK cell cytotoxicity, INF-γ production, and inhibiting NKG2D expression on NK cells [38].

In this study, we observed only a transient elevation of TNF-α and IL-10 levels following NEO-201 administration. Levels of these cytokines decreased toward baseline by 72 h from infusion in all patients, suggesting that this elevation is an acute inflammatory event and that NEO-201 does not determine a prolonged elevation of these cytokines. In our ongoing Phase II study, we are performing further mechanistic studies to evaluate the modulation of these cytokines not only in the peripheral blood but also in the TME*.*

We did not observe a significant difference in serum levels of soluble CEACAM-5 and CEACAM-6 between patients with SD and PD. On the contrary, patients with SD showed much lower baseline and post treatment serum levels of MICA compared to patients with PD, although this difference was not statistically significant. Patients with PD, who had high soluble MICA levels, also showed an impairment of NK activation markers. Conversely, patients with SD, who had low soluble MICA levels, did not show an impairment of NK activation markers, suggesting that soluble MICA could be a factor involved in the impairment of NK antitumor activity. Our observations correlate with several studies that proved that high soluble MICA levels correlate with poor prognosis in cancer patients [23–26].

Additional correlative immunophenotyping assays unexpectedly revealed that NEO-201 binds to a specific subset of highly immunosuppressive Tregs expressing the cell surface marker CD15s (sialyl-Lewis-X). CD15s is specifically expressed by activated, terminally differentiated and most suppressive FOXP3^high^ Tregs within the functionally heterogeneous array of human Tregs, and in fact has been posited to differentiate the immunosuppressive Treg population implicated in tumor immune tolerance from all other cytokine secreting Tregs, at least in serum [27, 39]. Treatment with NEO-201 was associated with a reduction of the percentage of circulating Tregs in one patient with SD in the 1.5 mg/kg cohort, while no reduction in circulating Tregs was observed in patients with PD in the same cohort. Although it remains to be determined the mechanism by which NEO-201 specifically targets and depletes Tregs in vivo, for example by ADCC vs CDC, the observed decrease in circulating Tregs in a subset of patients is intriguing. Tregs accumulation in the TME is associated with poor clinical prognosis in cancer patients [40–42] and is one of the factors that impairs the efficacy of the treatment with checkpoint inhibitors targeting the PD-1/PD-L1 pathway [43–45]. Reduction of immunosuppressive Tregs from circulation and in the TME could be a valid strategy to enhance anti-cancer immune responses and to improve efficacy of checkpoint inhibitors. One strategy to remove Tregs is to use mAbs to target and eliminate them, like NEO-201. The reduction in circulating Tregs after NEO-201 infusion in patients with SD was one of the rationales to combine NEO-201 with pembrolizumab in the ongoing phase II clinical trial in adults with checkpoint and chemo-resistant solid tumors [46].

This first in human study confirms what was observed in pre-clinical studies. NEO-201 has a potent ADCC-mediated killing activity against its target cells (i.e. neutrophils), and its potency is correlated to the activation status of NK cells, as shown in patients with SD. However, this study presents some weaknesses that are being addressed in both ongoing and future planned clinical studies with NEO-201. One of the weaknesses of this study is that we enrolled mostly patients with colorectal cancer who, although had high expression of NEO-201 target antigen, may have some intrinsic resistance to the anti-tumor activity of NEO-201. These were heavily pretreated patients in whom standard therapies had failed. These patients had microsatellite stable status, for whom immunotherapy is not indicated. Several factors involved in the TME of patients with colorectal cancer, such as accumulation of Tregs, MDSCs, and immunosuppressive cytokines, have been reported to determine resistance to immunotherapy [47]. In this regard, results from the phase 3 SUNLIGHT study (NCT0437187), conducted in patients with refractory metastatic colorectal cancer, showed a modest increase in median overall survival (OS) with third-line bevacizumab plus trifluridine/tipiracil compared to trifluridine/tipiracil alone (10.8 months vs 7.5 months), and the overall response rate was only 6.3% [48].

In our study, we reported SD in 4 patients with colorectal cancer, and we observed that SD could be linked to the low baseline level of soluble MICA and reduction of circulating Tregs after treatment with NEO-201. It is possible that NEO-201 may have an effect in depleting Tregs in the TME. Unfortunately, in this study, we did not have the possibility to evaluate the antitumor effect of NEO-201 in the TME. In addition, due to small sample size and heterogeneous tumor types in this study, the interpretation of our correlative studies is limited.

Moreover, we noticed that all patients with SD harbored mutations in KRAS and/or NRAS genes. Based on this observation we will continue to explore the impact of specific genetic alterations in several types of tumors on the efficacy of NEO-201 and to determine if there is a role in combining NEO-201 and precision cancer drugs based on specific genetic alterations.

To overcome these issues, in our ongoing phase II clinical trial, we are a) enlarging sample size; b) including different types of tumors that could be more sensitive to the immunotherapy than colorectal cancer (metastatic Non-Small Cell Lung Cancer (NSCLC), cervical cancer, Head and Neck Squamous Cell Carcinoma (HNSCC), uterine carcinoma who have progressed during or after front-line standard of care treatment including chemotherapy, checkpoint therapy and/or targeted therapy; c) evaluating the antitumoral activity of NEO-201 in the TME, including its ability to deplete Tregs, and if Tregs depletion in the TME is correlated to the clinical response to NEO-201 in combination with pembrolizumab; d) using NEO-201 at the RP2D with better timing of administration of filgrastim; e) evaluating the correlation with low baseline levels of soluble MICA with the clinical response to treatment with NEO-201 with a bigger sample size. If this correlation will be linked to a statistically significant difference between responders and non-responders, low soluble MICA level will be included as one of the eligibility criteria to select a more specific population that may benefit from treatment with NEO-201.

Another shortcoming is that neutrophils killed by NEO-201 through ADCC can act as a “sink”, removing NEO-201 from the bloodstream and reducing the amount of antibody available to kill tumor cells. To overcome this issue and maximize the level of NEO-201 available to the tumor we are planning to launch new studies to use NEO-201 with chemotherapy or other anti-cancer drugs during neutrophil nadir. This concept is reinforced by results obtained in animal models harboring human pancreatic and ovarian carcinoma, in which we tested the antitumor activity of NEO-201. In these animals, lacking circulating human neutrophils, NEO-201 showed a potent antitumor activity, resulting in a reduction of tumor volume and/or prolonged survival compared to controls [13, 14]. In addition, preclinical data has demonstrated that NEO-201 can bind and kill AML cell lines in vitro via ADCC [17, 35]. It is possible that hematological cells could be more sensitive than solid tumors to the ADCC or CDC mediated by NEO-201. For this reason, future clinical studies, using NEO-201, will target patients with hematological malignancies, such as AML.

## Conclusions

This first-in-human study of NEO-201 in solid tumors demonstrated that NEO-201 was well-tolerated and the RP2D was established at 1.5 mg/kg. Exploratory studies in serum suggested a correlation between maintenance of SD and lower baseline levels of soluble serum MICA, while serum levels of CEACAM-5 and CEACAM-6 were not related to the outcome of the treatment. In this study we also reported the ability of NEO-201 to bind to circulating Tregs. The reduction in circulating Tregs after NEO-201 infusion in patients with SD was one of the rationales to combine NEO-201 with pembrolizumab in the ongoing phase II clinical trial in adults with chemo-resistant solid tumors [46].

Patients with malignancies for which checkpoint inhibitors are clinically indicated, including NSCLC, HNSCC, uterine cancer, and cervical cancer, often do not respond to initial treatment and eventually become resistant to many therapies. Resistance to immunotherapy is a great challenge from a therapeutic standpoint and necessitates the development of novel approaches to circumvent resistance, including combination therapies. Combining NEO-201 with pembrolizumab in our ongoing phase II clinical trial could be a promising strategy to enhance immune system mediated anti-tumor activity for two reasons: 1) NEO-201 may overcome resistance to checkpoint inhibitors, by depleting Tregs involved in the immunosuppression of immune cells that play a role in the control of tumor growth; 2) NEO-201 can kill via ADCC and/or CDC, tumors expressing its target antigen in subjects for whom pembrolizumab is currently indicated.

## Supplementary Information

**Additional file 1:** **Supplementary Table 1.** Genetic alterations of all patients enrolled in the study.

**Additional file 2:** **Supplementary Table 2.** Report of all adverse events (grade 1-4) in all patients.

**Additional file 3:** **Supplementary Table 3.** IHC staining profile of patients enrolled in the study.

**Additional file 4:** **Supplementary Figure 1.** Time of administration of filgrastim affects NEO-201 PK. Correlation between timing of administration of filgrastim and NEO-201 serum concentrations after first NEO-201 infusion in patients that received NEO-201 at DL 2 and DL 1.5. A. Correlation between timing of administration of filgrastim and NEO-201 serum concentrations after first NEO-201 infusion in 4 patients of 2 mg/kg cohort (DL 2). B. Correlation between timing of administration of filgrastim and NEO-201 serum concentrations after first NEO-201 infusion in 6 patients of 1.5 mg/kg cohort (DL 1.5).

**Additional file 5:** **Supplementary Figure 2.** IHC staining of patients’ tumor tissue by murine version of NEO-201 (m16C3) A-C. Representative staining from malignant tissues (6 colon, 2 pancretic, and 2 breast cancer tissues). All images were obtained at 20X magnification. PT: patient.

**Additional file 6:** **Supplementary Figure 3.** Flow cytometry analysis of NEO-201 binding to CD4^+^/CD25^+^/CD127^-^/Foxp3^+^/CD15s^+^ population in whole PBMCs from patients treated with NEO-201 DL 1.5. Left plot: percentage of CD4^+^/NEO-201^+^ cells in whole viable PBMCs. Central plot: percentage of CD25^+^/CD127^-^ cells from CD4^+^/NEO-201^+^ cells. Right plot: percentage of Foxp3^+^/CD15s^+^ and Foxp3^+^/CD15s^-^ cells from CD25^+^/CD127^-^ cells. Data are presented as percentage of viable cells expressing cell-surface Treg cells markers. Positivity was determined by using fluorescence-minus-one controls. Analysis was performed using BD FACSuite software.

## Abbreviations

mAb: Monoclonal antibody
DL: Dose level
DLT: Dose limiting toxicity
MTD: Maximum tolerated dose
RP2D: Recommended phase 2 dose
SD: Stable disease
PD: Progressive disease
ICI: Immune checkpoint inhibitor
ADCC: Antibody-dependent cellular cytotoxicity
CDC: Complement-dependent cytotoxicity
CEACAM: Carcinoembryonic antigen-related cell adhesion molecule
IHC: Immunohistochemistry
NK: Natural killer
PK: Pharmacokinetics
NCI: National Cancer Institute
NIH: National Institutes of Health
ORR: Objective Response Rate
CR: Complete response
PR: Partial response
PFS: Progression Free Survival
EOI: End-of-infusion
Cmax: Maximum serum concentration
Cmin: Minimum serum concentration
AUC: Area under the serum concentration
ELISA: Enzyme-linked immunosorbent assay
FFPE: Formalin-fixed, paraffin-embedded
CRS: Cytokine release syndrome
PBMCs: Peripheral blood mononuclear cells
ANC: Absolute neutrophil count
AE: Adverse event
Tregs: Regulatory T cells
AML: Acute myeloid leukemia
MM: Multiple myeloma
MDSCs: Myeloid-derived suppressor cells
TME: Tumor microenvironment
NSCLC: Non-Small Cell Lung Cancer
HNSCC: Head and Neck Squamous Cell Carcinoma

## Declarations

### Availability of data and materials

All data generated or analyzed during this study are included in this published article [and its supplementary information files].

Data not shown in the manuscript are available from the corresponding author on reasonable request.
