# Correction: First-in-human phase 1 clinical trial of anti-core 1 O-glycans targeting monoclonal antibody NEO-201 in treatment-refractory solid tumors

**DOI:** 10.1186/s13046-023-02668-3

**Published:** 2023-04-26

**Authors:** Christopher B. Cole, Maria Pia Morelli, Massimo Fantini, Markku Miettinen, Patricia Fetsch, Cody Peer, William D. Figg, Tyler Yin, Nicole Houston, Ann McCoy, Stanley Lipkowitz, Alexandra Zimmer, Jung-min Lee, Miroslava Pavelova, Erin N. Villanueva, Kathryn Trewhitt, B. Brooke Solarz, Maria Fergusson, Sharon A. Mavroukakis, Anjum Zaki, Kwong Y. Tsang, Philip M. Arlen, Christina M. Annunziata

**Affiliations:** 1 Women’s Malignancies Branch, Center for Cancer Research, National Cancer Institute, National Institutes of Health, Bethesda, MD USA; 2 Precision Biologics, Inc, Bethesda, MD USA; 3 Laboratory of Pathology, Center for Cancer Research, National Cancer Institute, National Institutes of Health, Bethesda, MD USA; 4 Clinical Pharmacology Program, National Cancer Institute, National Institutes of Health, Bethesda, MD USA

**Correction:** ***J Exp Clin Cancer Res*** **42, 76 (2023)**

**https://doi.org/10.1186/s13046-023-02649-6**

Following publication of the original article [1], an error was identified in Fig. 2, specifically:

- Fig. 2b - CL/F (mL/hr) needs to be corrected in CL/F (L/hr)

Furthermore, two Supplementary Materials should also be uploaded. The added Supplementary Materials are:

- Additional file 7: Supplementary Materials and Methods.
- Additional file 8: NEO-201 protocol.

This modification does not affect the integrity of the results.

**Fig. 2:**
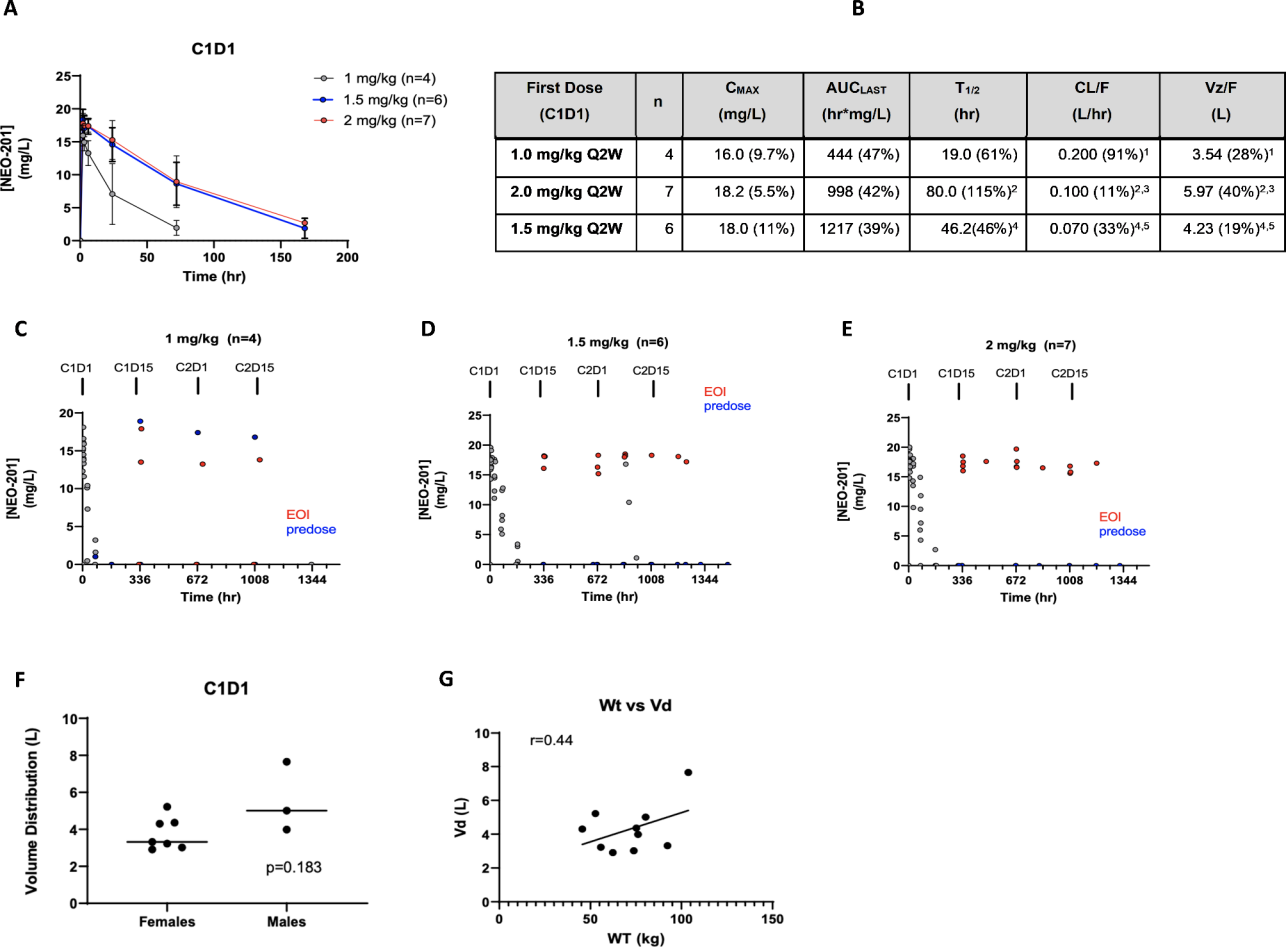
Clinical Pharmacokinetics of NEO-201 **A**. NEO-201 serum concentrations averaged for each dose level and plotted over time. **B.** Noncompartmental analysis of first dose of NEO-201 including the mean estimates for relevant PK parameters during dense PK sampling following the first dose given on C1D1. *Data presented as arithmetic means (%CV) due to low numbers in each group. ^1^ Two patients had inaccurate estimates of C1D1 clearance and volume due to an overestimation of AUCinf. ^2^ One patient had insufficient terminal data to accurately estimate a half-life, thus clearance and volume too. ^3^ Four patients had inaccurate estimates of C1D1 clearance and volume due to an overestimation of AUCinf. ^4^ One patient had insufficient terminal data to accurately estimate a half-life, thus clearance and volume too. ^5^ Two patients had inaccurate estimates of C1D1 clearance and volume due to an overestimation of AUCinf. **C.** Individual NEO-201 serum concentration–time profiles of 1.0 mg/kg cohort. **D-E**. Individual NEO-201 serum concentration–time profiles of patients in 1.5 mg/kg cohort (**D**) and 2.0 mg/kg cohort (**E**). **F-G**. Differences in NEO-201 distribution volume by sex (**F**) and body size (**G**)

## Electronic supplementary material

Below is the link to the electronic supplementary material.

Additional file 7: Supplementary Materials and Methods.

Additional file 8: NEO-201 protocol.

## References

[1] Cole CB, Morelli MP, Fantini M (2023). First-in-human phase 1 clinical trial of anti-core 1 O-glycans targeting monoclonal antibody NEO-201 in treatment-refractory solid tumors. J Exp Clin Cancer Res.

