# Supplementary material for: First-in-human phase 1 clinical trial of anti-core 1 O-glycans targeting monoclonal antibody NEO-201 in treatment-refractory solid tumors
Source: J Exp Clin Cancer Res. 2023 Mar 29;42:76. doi: 10.1186/s13046-023-02649-6 (PMC10053355; doi:10.1186/s13046-023-02649-6)
Supplement: Supplementary file 1 — Additional file 1: Supplementary Table 1. Genetic alterations of all patients enrolled in the study. [file 13046_2023_2649_MOESM1_ESM.docx]

| **Pt #** | **Cohort** | **Cancer type** | **BRAF** | **RAS** | **TP53** | **MSI** | **PDL-1** | **TMB status** | **Other mutations** | **Previous immunotherapies or targeted therapies** |
| --- | --- | --- | --- | --- | --- | --- | --- | --- | --- | --- |
| 1 | 1.0 mg/kg | Colorectal | N.A. | KRAS WT | N.A. | MSS | N.A. | N.A. |  | Anti VEGF therapy (bevacizumab): anti EGFR therapy (panitumumab) |
| 2 | 1.0 mg/kg | Colorectal | TRIM24-BRAF fusion | KRAS and NRAS WT | TP53 A161T mutation | MSS | N.A. | Intermediate  (7.99 muts/Mb) |  | Anti VEGF therapy (bevacizumab): anti EGFR therapy (panitumumab); anti BRAF therapy (dafrafenib); anti PD-1 therapy (pembrolizumab) |
| 3 | 1.0 mg/kg | Pancreas | N.A. | N.A. | N.A. | N.A. | N.A. | N.A. |  |  |
| 4 | 1.0 mg/kg | Colorectal | BRAF D594G-subclonal | KRAS G12S mutation | Mutated | MSS | N.A. | N.A. |  | Anti VEGF therapy (bevacizumab) |
| 5 | 2.0 mg/kg | Colorectal | WT | WT | Somatic mutations | MSS | N.A. | N.A. | BRCA1 mutated | Anti VEGF therapy (bevacizumab) |
| 6 | 2.0 mg/kg | Colorectal | N.A. | KRAS G12D mutation | N.A. | MSS | N.A. | N.A. | PIK3A mutated | Anti VEGF therapy (bevacizumab) |
| 7 | 2.0 mg/kg | Colorectal | N.A. | KRAS and NRAS WT | N.A. | MSS | N.A. | N.A. |  | Anti VEGF therapy (bevacizumab): anti EGFR therapy (cetuximab) |
| 8 | 2.0 mg/kg | Pancreas | N.A. | KRAS G12V mutation | TP53 C238Y mutation | MSS | Negative | Intermediate | BRCA1 mutated;  RRM1, TOPO1, TS, TUBB3 positive |  |
| 9 | 2.0 mg/kg | Colorectal | WT | KRAS WT; NRAS mutated | N.A. | MSS | N.A. | Low  (3 muts/mb) |  | Anti VEGF therapy (bevacizumab); anti EGFR therapy (panitumumab) |
| 10 | 2.0 mg/kg | Breast (ER^+^/PR^-^/HER2^-^) | N.A. | N.A. | N.A. | N.A. | N.A. | N.A. |  | Anti estrogen therapy (fulvestrant) |
| 11 | 2.0 mg/kg | Colorectal | N.A. | KRAS WT | N.A. | MSS | N.A. | N.A. |  | anti EGFR therapy (panitumumab) |
| 12 | 1.5 mg/kg | Colorectal | WT | KRAS and NRAS WT | N.A. | MSS | Negative | N.A. |  | Anti VEGF therapy (bevacizumab): anti EGFR therapy (panitumumab) |
| 13 | 1.5 mg/kg | Colorectal | WT | KRAS mutated (exon 12); NRAS mutated | mutated | MSS | N.A. | N.A. |  | Anti VEGF therapy (bevacizumab) |
| 14 | 1.5 mg/kg | Colorectal | N.A. | KRAS mutated (exon 2) | N.A. | MSI | N.A. | N.A. | MMR proficient | Anti VEGF therapy (bevacizumab) |
| 15 | 1.5 mg/kg | Breast  (ER^+^/PR^-^/HER2^-^) | N.A. | N.A. | N.A. | MSS | Negative | Intermediate  (9 muts/Mb) |  | Anti estrogen therapy (fulvestrant); anti CTLA-4 + anti PD-1 therapy (ipilimumab+ nivolumab) |
| 16 | 1.5 mg/kg | Pancreas | N.A. | N.A. | N.A. | N.A. | N.A. | N.A. | positive for CK7 and weakly focally positive for GATA3; negative for CDX2, CK20, calretinin and WT1 | Anti CTLA-4 + anti PD-1 therapy (ipilimumab+ nivolumab) |
| 17 | 1.5 mg/kg | Pancreas | N.A. | N.A. | N.A. | MSS | N.A. | N.A. | CK7 and CK19 positive; CK20, TTF1, and CDX2 negative | anti PD-1 therapy (pembrolizumab) |

**Supplementary Table 1. Genetic alterations of all patients enrolled in the study**

**Table legend**. WT: Wild Type; MSS: Microsatellite Stable; N.A.: Not Available

Patient #4, #6, #9, #13 are patients with stable disease
