# Supplementary material for: First-in-human phase 1 clinical trial of anti-core 1 O-glycans targeting monoclonal antibody NEO-201 in treatment-refractory solid tumors
Source: J Exp Clin Cancer Res. 2023 Mar 29;42:76. doi: 10.1186/s13046-023-02649-6 (PMC10053355; doi:10.1186/s13046-023-02649-6)
Supplement: Supplementary file 2 — Additional file 2: Supplementary Table 2. Report of all adverse events (grade 1-4) in all patients. [file 13046_2023_2649_MOESM2_ESM.docx]

| **Body System,**  Adverse Event | **Dose Level 1**  **1 mg/kg** n= number of events in 4 patients/  12 doses (%) | | | | **Dose Level 2**  **2 mg/kg**  n= number of events in 7 patients/  34 doses (%) | | | | **Dose Level 1.5**  **1.5 mg/kg**  n= number of events in 6 patients/  23 doses (%) | | | | **Cumulative Incidence**  n = number of events in 17 patients/  69 doses (%) |
| --- | --- | --- | --- | --- | --- | --- | --- | --- | --- | --- | --- | --- | --- |
|  | **Gr 1** | **Gr 2** | **Gr 3** | **Gr 4** | **Gr 1** | **Gr 2** | **Gr 3** | **Gr 4** | **Gr 1** | **Gr 2** | **Gr 3** | **Gr 4** |  |
| **Blood and Lymphatic System Disorders** | | | | |  |  |  |  |  |  |  |  |  |
| Anemia | 2 (17%) | 4 (33%) | -- | -- | 4 (12%) | 9 (26%) | 2 (6%) | -- | 3 (13%) | 3 (13%) | -- | -- | 27 (39%) |
| Febrile Neutropenia | -- | -- | -- | -- | -- | -- | 2 (6%) | 1 (3%) | -- | -- | 1 (4%) | -- | 4 (6%) |
| **Cardiac Disorders** | | | | |  |  |  |  |  |  |  |  |  |
| Electrocardiogram QTc prolonged | 1 (8%) | -- | -- | -- | -- | -- | -- | -- | -- | -- | -- | -- | 1 (1%) |
| Tachycardia | 1 (8%) | -- | -- | -- | 1 (3%) | -- | -- | -- | -- | 1 (4%) | -- | -- | 2 (3%) |
| **Gastrointestinal disorders** | | | | |  |  |  |  |  |  |  |  |  |
| Constipation | -- | -- | -- | -- | 1 (3%) | 1 (3%) |  | -- | -- | -- | -- | -- | 2 (3%) |
| Diarrhea | 1 (8%) | -- | -- | -- | 1 (3%) | -- | -- | -- | -- | -- | -- | -- | 2 (3%) |
| Gum pain | -- | -- | -- | -- | 1 (3%) | -- | -- | -- | -- | -- | -- | -- | 1 (1%) |
| Mucositis | -- | -- | -- | -- | 1 (3%) | 1 (3%) | -- | -- | -- | -- | -- | -- | 2 (3%) |
| Nausea | 2 (17%) | -- | -- | -- | 1 (3%) | -- | -- | -- | -- | -- | -- | -- | 3 (4%) |
| Rectal Pain | -- | 1 (8%) | -- | -- | -- | -- | -- | -- | -- | -- | -- | -- | 1 (1%) |
| Vomiting | 1 (8%) | -- | -- | -- | 1 (3%) | -- | -- | -- | -- | -- | -- | -- | 2 (3%) |
| **General Disorders and Administration Site Conditions** | | | | |  |  |  |  |  |  |  |  |  |
| Chills/Rigors | 2 (17%) | 1 (8%) | -- | -- | 2 (6%) | 1 (3%) | -- | -- | -- | -- | -- | -- | 6 (9%) |
| Fatigue | 1 (8%) | 1 (8%) | -- | -- | -- | 2 (6%) | -- | -- | 2 (9%) | -- | -- | -- | 6 (9%) |
| Fever | 6 (50%) | 1 (8%) | -- | -- | 4 (12%) | 1 (3%) | -- | -- | 4 (17%) | -- | -- | -- | 16 (23%) |
| Flu like symptoms | -- | 1 (8%) | -- | -- | -- | -- | -- | -- | -- | -- | -- | -- | 1 (1%) |
| Malaise | -- | -- | -- | -- | -- | -- | -- | -- | 1 (4%) | -- | -- | -- | 1 (1%) |
| Non-cardiac chest pain | -- | -- | -- | -- | -- | -- | -- | -- | 1 (4%) | -- | -- | -- | 1 (1%) |
| Weakness | 1 (8%) | -- | -- | -- | -- | -- | -- | -- | -- | -- | -- | -- | 1 (1%) |
| **Infections and Infestations** | | | | |  |  |  |  |  |  |  |  |  |
| Sepsis | -- | -- | -- | -- | -- | -- | -- | 1 (3%) | -- | -- | -- | -- | 1 (1%) |
| **Injury, Poisoning and procedural complications** | | | | |  |  |  |  |  |  |  |  |  |
| Infusion related reaction | -- | 8 (66%) | -- | -- | -- | 7 (21%) | -- | -- | -- | 3 (13%) | -- | -- | 18 (26%) |
| **Investigations** | | | | |  |  |  |  |  |  |  |  |  |
| APTT prolonged | 1 (8%) | -- | -- | -- | -- | -- | -- | -- | -- | -- | -- | -- | 1 (1%) |
| Alkaline phosphatase increased | -- | -- | -- | -- | 1 (3%) | 1 (3%) | -- | -- | 1 (4%) | -- | -- | -- | 3 (4%) |
| Aminotransferase Increased | -- | -- | -- | -- | 2 (6%) | -- | -- | -- | 1 (4%) | -- | -- | -- | 3 (4%) |
| Blood bilirubin increased | -- | -- | -- | -- | -- | -- | -- | -- | -- | -- | -- | -- | 1 (1%) |
| C-reactive protein high | -- | -- | -- | -- | 2 (6%) | -- | -- | -- | -- |  |  |  | 2 (3%) |
| Creatinine increased | 1 (8%) | -- | -- | -- | -- | -- | -- | -- | -- | -- | -- | -- | 1 (1%) |
| Lymphocyte count decreased | 1 (8%) | -- | 1 (8%) | -- | -- | 6 (18%) | 4 (12%) | 2 (6%) | 2 (9%) | 1 (4%) | -- | 1 (4%) | 18 (26%) |
| Neutrophil count decreased | -- | -- | 2 (17%) | 4 (33%) | 1 (3%) | -- | 2 (6%) | 10 (29%) | 2 (9%) | -- | 1 (4%) | 7 (30%) | 29 (42%) |
| Platelet count decreased | 1 (8%) | -- | -- | -- | 5 (15%) | 1 (3%) | -- | -- | 2 (9%) | -- | -- | -- | 9 (13%) |
| Weight loss | -- | -- | -- | -- | 2 (6%) | -- | -- | -- | -- | -- | -- | -- | 2 (3%) |
| White blood cell decreased | 4 (33%) | -- | 2 (17%) | -- | 2 (6%) | 3 (9%) | 7 (21%) | 2 (6%) | -- | 2 (9%) | 1 (4%) | 4 (17%) | 27 (39%) |
| **Metabolism and Nutrition Disorders** | | | | |  |  |  |  |  |  |  |  |  |
| Anorexia | -- | -- | -- | -- | 1 (3%) | -- | -- | -- | -- | -- | -- | -- | 1 (1%) |
| Hyperglycemia | -- | -- | -- | -- | 1 (3%) | -- | -- | -- | -- | -- | -- | -- | 1 (1%) |
| Hyperuricemia | 1 (8%) | -- | -- | -- | -- | -- | -- | -- | -- | -- | -- | -- | 1 (1%) |
| Hypoalbuminemia | -- | -- | -- | -- | -- | -- | -- | -- | 1 (4%) | -- | -- | -- | 1 (1%) |
| Hypocalcemia | 1 (8%) | -- | -- | -- | -- | -- | -- | -- | -- | -- | -- | -- | 1 (1%) |
| Hyponatremia | 2 (17%) | -- | -- | -- | 1 (3%) | -- | -- | -- | 1 (4%) | -- | -- | -- | 4 (6%) |
| Hypophosphatemia | -- | -- | -- | -- | -- | 1 (3%) | -- | -- | -- | -- | -- | -- | 1 (1%) |
| **Musculoskeletal and Connective Tissue disorders** | | | | |  |  |  |  |  |  |  |  |  |
| Pain in extremity | -- | -- | -- | -- | 1 (3%) | 1 (3%) | -- | -- | 1 (4%) | -- | -- | -- | 3 (4%) |
| **Nervous System Disorders** | | | | |  |  |  |  |  |  |  |  |  |
| Headache | 1 (8%) | -- | -- | -- | 1 (3%) | -- | -- | -- | 1 (4%) |  |  |  | 3 (4%) |
| Lethargy | -- | -- | -- | -- | -- | 1 (3%) | -- | -- | -- | -- | -- | -- | 1 (1%) |
| Paresthesia | -- | -- | -- | -- | -- | -- | -- | -- | 1 (4%) | -- | -- | -- | 1 (1%) |
| Peripheral neuropathy | -- | -- | -- | -- | -- | 1 (3%) | -- | -- | -- | -- | -- | -- | 1 (1%) |
| **Renal and urinary disorders** | | | | |  |  |  |  |  |  |  |  |  |
| Bladder Spasm | -- | -- | -- | -- | 1 (3%) | -- | -- | -- | -- | -- | -- | -- | 1 (1%) |
| **Respiratory, Thoracic and Mediastinal Disorders** | | | | |  |  |  |  |  |  |  |  |  |
| Cough | 1 (8%) | -- | -- | -- | -- | -- | -- | -- | -- | -- | -- | -- | 1 (1%) |
| Dyspnea | -- | 2 (17%) | -- | -- | -- | -- | -- | -- | 1 (4%) | -- | -- | -- | 3 (4%) |
| **Skin and Subcutaneous tissue disorders** | | | | |  |  |  |  |  |  |  |  |  |
| Night Sweats | -- | -- | -- | -- | 2 (6%) | -- | -- | -- | -- | -- | -- | -- | 2 (3%) |
| Rash | 1 (8%) | -- | -- | -- | 1 (3%) | -- | -- | -- | -- | -- | -- | -- | 2 (3%) |
| **Vascular Disorders** | | | | |  |  |  |  |  |  |  |  |  |
| Hypertension | -- | -- | 1 (8%) | -- | -- | -- | -- | -- | -- | -- | -- | -- | 1 (1%) |
| Hypotension | 1 (8%) | 1 (8%) | -- | -- | -- | -- | -- | -- | -- | -- | -- | -- | 2 (3%) |

**Supplemental Table 2 Report of all adverse events (grade 1-4) in all patients**
