# Supplementary material for: First-in-human phase 1 clinical trial of anti-core 1 O-glycans targeting monoclonal antibody NEO-201 in treatment-refractory solid tumors
Source: J Exp Clin Cancer Res. 2023 Mar 29;42:76. doi: 10.1186/s13046-023-02649-6 (PMC10053355; doi:10.1186/s13046-023-02649-6)
Supplement: Supplementary file 3 — Additional file 3: Supplementary Table 3. IHC staining profile of patients enrolled in the study. [file 13046_2023_2649_MOESM3_ESM.docx]

| **Patient #** | **Cohort** | **Cancer type** | **% Tissue NEO-201 positive** | **Staining intensity** |
| --- | --- | --- | --- | --- |
| 1 | 1.0 mg/kg | Colorectal | 100% | 3+ |
| 2 | 1.0 mg/kg | Colorectal | 100% | 3+ |
| 3 | 1.0 mg/kg | Pancreas | >90% | 3+ |
| 4 | 1.0 mg/kg | Colorectal | 100% | 3+ |
|  | | | | |
| 5 | 2.0 mg/kg | Colorectal | >90% | 3+ |
| 6 | 2.0 mg/kg | Colorectal | 100% | 3+ |
| 7 | 2.0 mg/kg | Colorectal | 100% | 3+ |
| 8 | 2.0 mg/kg | Pancreas | 100% | 3+ |
| 9 | 2.0 mg/kg | Colorectal | >90% | 3+ |
| 10 | 2.0 mg/kg | Breast (ER^+^/PR^-^/HER2^-^) | >90% | 3+ |
| 11 | 2.0 mg/kg | Colorectal | 100% | 3+ |
|  | | | | |
| 12 | 1.5 mg/kg | Colorectal | 100% | 3+ |
| 13 | 1.5 mg/kg | Colorectal | 100% | 3+ |
| 14 | 1.5 mg/kg | Colorectal | >90% | 3+ |
| 15 | 1.5 mg/kg | Breast (ER^+^/PR^-^/HER2^-^) | 20% | 3+ |
| 16 | 1.5 mg/kg | Pancreas | Tissue lost | NA |
| 17 | 1.5 mg/kg | Pancreas | Sample not adequate | NA |

**Supplementary Table 3 IHC staining profile of patients enrolled in the study**
