# Supplementary figures and images for: First-in-human phase 1 clinical trial of anti-core 1 O-glycans targeting monoclonal antibody NEO-201 in treatment-refractory solid tumors
Source: J Exp Clin Cancer Res. 2023 Mar 29;42:76. doi: 10.1186/s13046-023-02649-6 (PMC10053355; doi:10.1186/s13046-023-02649-6)

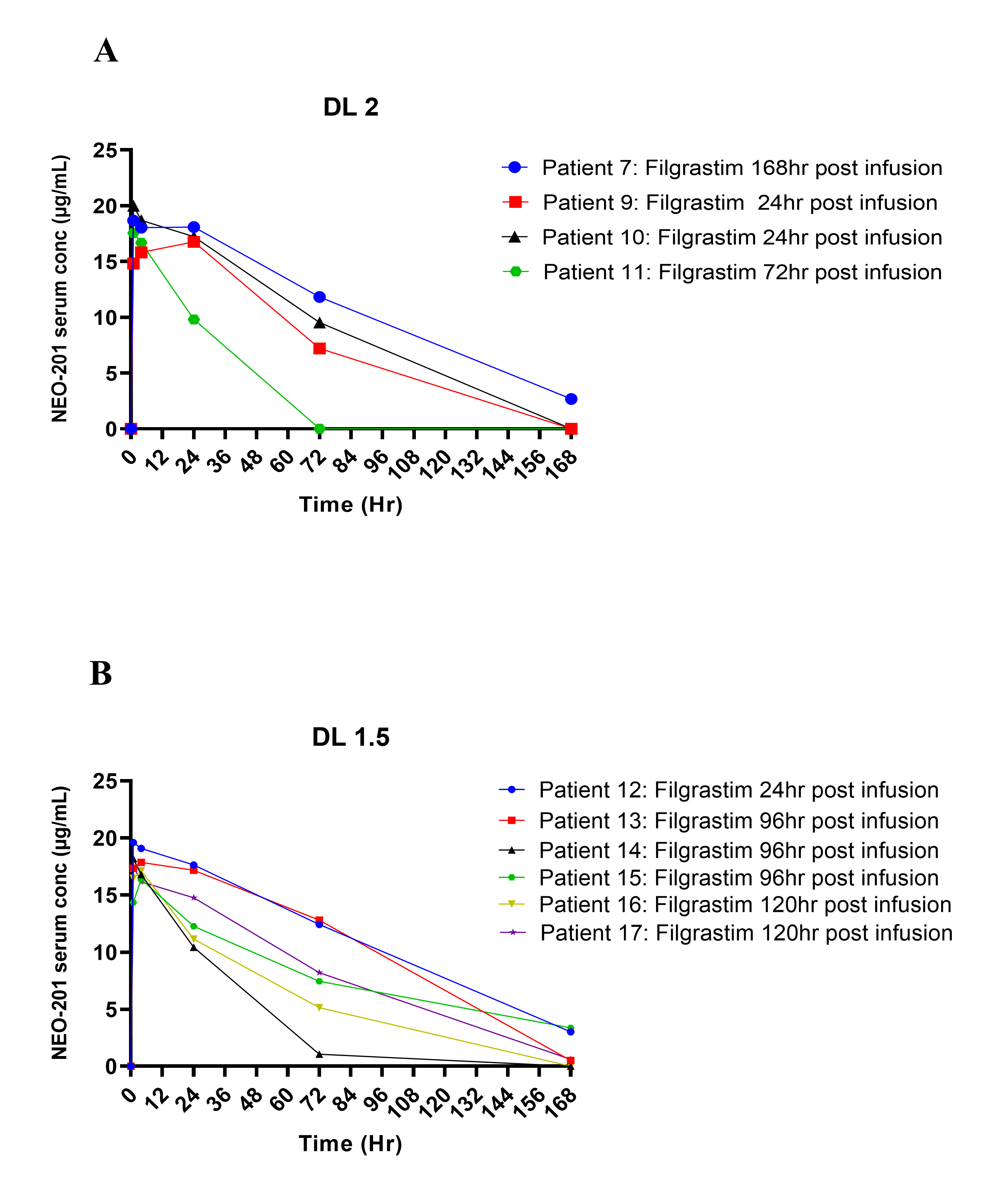

Supplement: Supplementary file 4 — Additional file 4: Supplementary Figure 1. Time of administration of filgrastim affects NEO-201 PK. Correlation between timing of administration of filgrastim and NEO-201 serum concentrations after first NEO-201 infusion in patients that received NEO-201 at DL 2 and DL 1.5. A. Correlation between timing of administration of filgrastim and NEO-201 serum concentrations after first NEO-201 infusion in 4 patients of 2 mg/kg cohort (DL 2). B. Correlation between timing of administration of filgrastim and NEO-201 serum concentrations after first NEO-201 infusion in 6 patients of 1.5 mg/kg cohort (DL 1.5). [file 13046_2023_2649_MOESM4_ESM.tif]

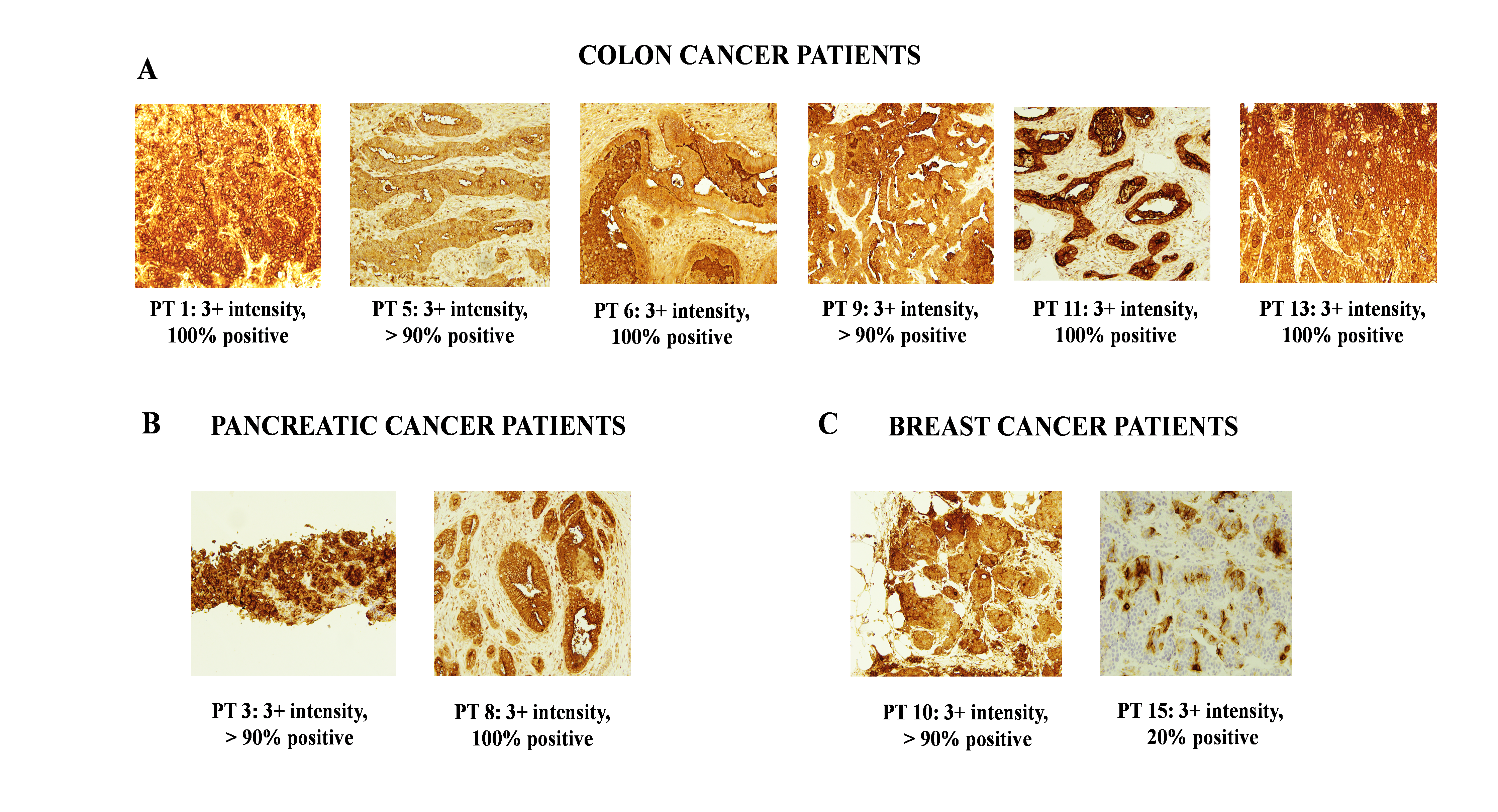

Supplement: Supplementary file 5 — Additional file 5: Supplementary Figure 2. IHC staining of patients’ tumor tissue by murine version of NEO-201 (m16C3) A-C. Representative staining from malignant tissues (6 colon, 2 pancretic, and 2 breast cancer tissues). All images were obtained at 20X magnification. PT: patient. [file 13046_2023_2649_MOESM5_ESM.tif]

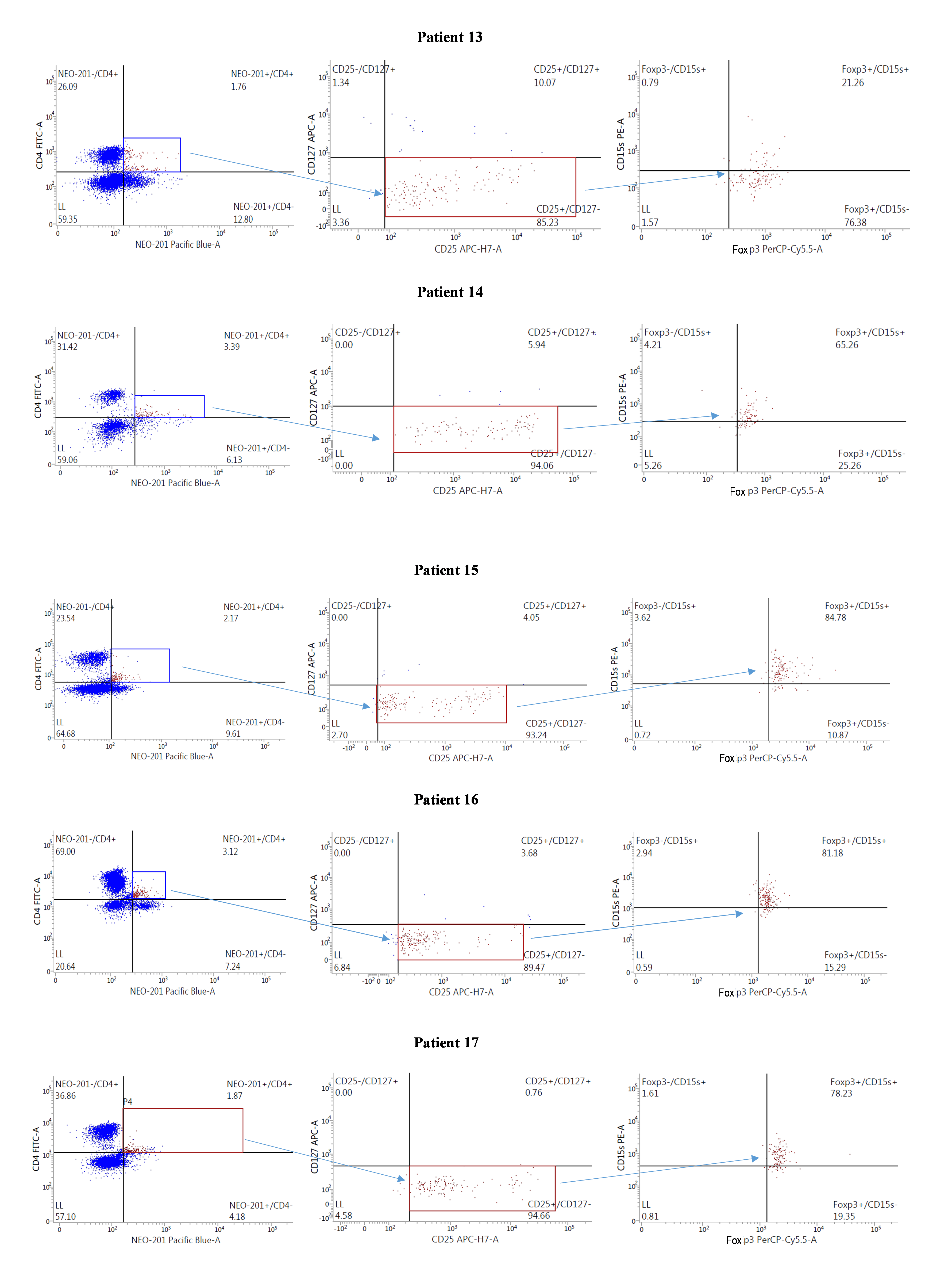

Supplement: Supplementary file 6 — Additional file 6: Supplementary Figure 3. Flow cytometry analysis of NEO-201 binding to CD4+/CD25+/CD127-/Foxp3+/CD15s+ population in whole PBMCs from patients treated with NEO-201 DL 1.5. Left plot: percentage of CD4+/NEO-201+ cells in whole viable PBMCs. Central plot: percentage of CD25+/CD127- cells from CD4+/NEO-201+ cells. Right plot: percentage of Foxp3+/CD15s+ and Foxp3+/CD15s- cells from CD25+/CD127- cells. Data are presented as percentage of viable cells expressing cell-surface Treg cells markers. Positivity was determined by using fluorescence-minus-one controls. Analysis was performed using BD FACSuite software. [file 13046_2023_2649_MOESM6_ESM.tif]
