# Supplementary material for: Correction: First-in-human phase 1 clinical trial of anti-core 1 O-glycans targeting monoclonal antibody NEO-201 in treatment-refractory solid tumors
Source: J Exp Clin Cancer Res. 2023 Apr 26;42:102. doi: 10.1186/s13046-023-02668-3 (PMC10131449; doi:10.1186/s13046-023-02668-3)
Supplement: Supplementary file 1 — Additional file 7: Supplementary Materials and Methods. [file 13046_2023_2668_MOESM1_ESM.pdf]

Abbreviated Title: Phase 1 NEO-201
Sponsor Protocol #: PB-1801
Version Date: January 8, 2020
Amendment #: 5

Title: PHASE 1 WITH EXPANSION COHORTS IN A STUDY OF NEO-201 IN ADULTS WITH CHEMO-RESISTANT SOLID TUMORS

Study Chair:

Christina M. Annunziata, M.D., Ph.D.
Women's Malignancies Branch (WMB)
National Institutes of Health
10 Center Drive, Room 3B43A
Bethesda, MD 20892
Telephone: 301-402-7189
Fax: 301-402-0172
E-mail: annunzic@mail.nih.gov

Sponsor Information:

**Medical Advisor**	Philip M. Arlen, M.D., Chief Medical Officer<br>Precision Biologics, Inc.<br>4814 Del Ray Avenue<br>Bethesda, Maryland 20814<br>Cell Phone: 301-728-4883<br>Tel: 301-500-8648<br>FAX: 240-669-9828<br>e-mail: philip.arlen@Precision-Biologics.com
**Sponsor:**	Precision Biologics, Inc.<br>4814 Del Ray Avenue<br>Bethesda, Maryland 20814

Investigational Agents:

Drug Name:	NEO-201 (h16C3)
IND Number:	134656
Sponsor:	PRECISION BIOLOGICS, Inc.
Manufacturer:	Catalent Pharma Solutions, LLC

CONFIDENTIAL:
NOT TO BE DISCLOSED WITHOUT PERMISSION FROM PRECISION BIOLOGICS - FOR QUALIFIED INVESTIGATORS AND THEIR IRBS ONLY

TABLE OF CONTENTS

Table of Contents 2
List of Abbreviations 6
Statement of Compliance 7
Protocol Summary 8
Phase 1 with Expansion Cohorts in a Study of NEO-201 In Adults with Chemo-resistant Solid Tumors 8
Schematic of Study Design 10
1 Introduction: Background Information and Scientific Rationale 11
1.1 Background Information 11
1.2 Rationale 26
1.3 Potential Risks and Benefits 26
2 Objectives and Purpose 28
2.1 Primary Objective: 28
2.2 Secondary Objective(s) 28
2.3 Exploratory Objectives 28
3 Study Design and Endpoints 29
3.1 Description of the Study Design 29
3.2 Study Endpoints 34
4 Study Enrollment and Withdrawal 37
4.1 Participant Inclusion Criteria 37
4.2 Participant Exclusion Criteria 38
4.3 Strategies for Recruitment and Retention 39
4.4 Subject Screening, Enrollment and Registration Procedures 39
4.5 Participant Withdrawal or Termination 40
4.6 Premature Termination or Suspension of Study 41
5 Study Agent 41
5.1 Study Agent and Control Description 41
6 Study Procedures and Schedule 44
6.1 Study Procedures/Evaluations 44
6.2 Study Schedule 48

CONFIDENTIAL:
NOT TO BE DISCLOSED WITHOUT PERMISSION FROM PRECISION BIOLOGICS - FOR QUALIFIED INVESTIGATORS AND THEIR IRBS ONLY

6.3 Concomitant Medications, Treatments, and Procedures 53
7 Assessment of Safety 53
7.1 Specification of Safety Parameters 53
7.2 Classification of an Adverse Event 55
7.3 Time Period and Frequency for Event Assessment and Follow Up 56
7.4 Reporting Procedures 57
7.5 Study Halting Rules 59
7.6 Safety Oversight 59
8 Clinical Monitoring 59
8.1 Good Clinical Practice 59
8.2 Ethical Considerations 60
8.3 Subject Information and Informed Consent 60
8.4 Subject Confidentiality 60
8.5 Protocol Compliance 60
8.6 Data Collection and Entry 61
8.7 Sponsor Study monitoring 61
8.8 On-site audits 61
8.9 Data entry completion 62
8.10 Record Retention 62
9 Statistical Considerations 62
9.1 Statistical and Analytical Plan 62
9.2 Statistical Hypotheses 63
9.3 Analysis Datasets 63
9.4 Description of Statistical Methods 63
9.5 Sample Size 66
9.6 Measures to Minimize Bias 66
10 Source Documents and Access to Source Data/Documents 66
11 Quality Assurance (QA) and Quality Control (QC) 67
12 Ethics/Protection of Human Subjects 67
12.1 Ethical Standard 67
12.2 Institutional Review Board 67

12.3 Informed Consent Process ... 68
12.4 Participant and Data Confidentiality ... 69
12.5 Future Use of Stored Specimens ... 70
13 Data Handling and Record Keeping ... 70
13.1 Data Collection and Management Responsibilities ... 70
13.2 Study Records Retention ... 71
13.3 Protocol deviations ... 72
13.4 Publication and Data Sharing Policy ... 72
14 Study Administration ... 73
14.1 Study Leadership ... 73
15 Conflict of Interest Policy ... 73
16 Appendices ... 74
16.1 APPENDIX A-Performance Status Criteria ... 74
16.2 Appendix B: Hypersensitivity/Infusion Reaction Algorithm ... 75
16.3 Appendix C: Study Calendar ... 76
16.4 Appendix D: Response Criteria ... 78
17 Investigator Agreement ... 84
18 References ... 85

CONFIDENTIAL:
NOT TO BE DISCLOSED WITHOUT PERMISSION FROM PRECISION BIOLOGICS - FOR QUALIFIED INVESTIGATORS AND THEIR IRBS ONLY

Table of Figures

Figure 1: NEO-201 IHC of tumor tissue microarrays compared with normal tissues. Positive immuno reactivity is the percentage of core samples that stained positive for NEO-201. 14

Figure 2: Immunohistochemistry peroxidase staining of NEO-201 mAb (16C3) and commercial CEACAM 5/6 antibodies in A. Colon cancer and normal colon tissue, B. Pancreas cancer and normal pancreas tissue, and C. Lung cancer and normal lung tissue. 14

Figure 3: Immunoreactivity to NEO-201 in over 600 samples for ovarian cancer from Roswell Park Cancer Institute 15

Figure 4: NEO-201 (Humanized 16C3 (h16C3) anti-tumor efficacy study in the CFPAC-1 tumor xenograft model 16

Figure 5: Humanized 16C3 anti-tumor efficacy results in CFPAC-1 tumor-bearing mice 17

Figure 6: Humanized 16C3 anti-tumor efficacy results 17

Figure 7: ADCC and CDC Killing in Pancreatic Cancer Cell Lines 18

Figure 8: Tissue Distribution (cpm/g) of [^{125}I]h16C3 Antibody in Male Nude Mice with CFPAC-1 Tumors 19

Figure 9: Tissue Distribution (cpm/g) of [^{125}I]h16C3 Antibody in Female Nude Mice with CFPAC-1 Tumors 19

Figures 10: A. Neutrophil Counts in Male and B. Female Cynomolgous Monkeys after NEO-201 24

Figure 11: Study Schema* 29

Table of Tables

Table 1: NEO-201 IHC Profile: Results from Lung Cancer Microarray 15

Table 2: Dose Escalation Schedule 30

Table 3: Rules for Dose Escalation and Definition of Maximum Tolerated Dose (MTD) 31

LIST OF ABBREVIATIONS

ADCC	Antibody-dependent cellular cytotoxicity
AE	Adverse Event
AUC	Area Under the Curve
CEACAM	carcinoembryonic antigen- related cell adhesion molecules
CDC	complement dependent cytotoxicity
Cmax	maximum (or peak) serum concentration
Cmin	Minimum serum concentration
CR	Complete response
CTCAE	Common Terminology Criteria for Adverse Events
DLT	Dose limiting toxicities
ECOG	Eastern Cooperative Oncology Group
EDC	Electronic Data Capture
FDA	U.S. Food and Drug Administration
IHC	Immunohistochemistry
IND	Investigational New Drug Exemption
IRB	Institutional Review Board
MDSCs	myeloid derived suppressor cells
mNSCLC	metastatic non-small cell lung cancer
MTD	maximum tolerated dose
NOAEL	no observed adverse event level
ORR	Objective Response Rate
PR	Partial Response
RECIST	Response Evaluation Criteria In Solid Tumors
RP2D	Recommended Phase 2 Dose
SAE	Serious Adverse Event
SD	Stable disease
TCR	Tissue Cross Reactivity
Treg	Regulatory T cells

STATEMENT OF COMPLIANCE

This trial will be carried out in accordance with Good Clinical Practice (GCP) as required by the following*:* United States (US) Code of Federal Regulations (CFR) applicable to clinical studies (45 CFR Part 46, 21 CFR Part 50, 21 CFR Part 56, 21 CFR Part 312, and/or 21 CFR Part 812)

ICH E6

All key personnel (all individuals responsible for the design and conduct of this trial) have completed Human Subjects Protection Training.

I agree to ensure that all staff members involved in the conduct of this study are informed about their obligations in meeting the above commitments.

Principal Investigator: Christina M. Annunziata, M.D., Ph.D.__ Print/Type Name

Signed: ____________________________________ Signature

Date: ______________

Abbreviated Title: Phase 1 + expansion in NEO-201
Version Date: January 8, 2020

PROTOCOL SUMMARY

Title:	PHASE 1 WITH EXPANSION COHORTS IN A STUDY OF NEO-201 IN ADULTS WITH CHEMO-RESISTANT SOLID TUMORS
Precis:	This is an open label, first-in-human standard 3 + 3 phase 1 dose escalation design to determine the MTD and RP2D of the monoclonal antibody NEO-201 in adults with refractory or recurrent advanced solid tumors, a majority of which tested positive for the NEO-201 antigen in IHC laboratory studies. Subjects will be given NEO-201 intravenously every two weeks for two doses (1 cycle = 28 days) in groups of 3-6 subjects at doses of 1 mg/kg, 1.5 mg/kg, 2 mg/kg, 4 mg/kg, or 6 mg/kg. Additional dose levels (de-escalation to 3 mg/kg or 5 mg/kg) may be explored if -DLTs occur in subjects receiving NEO-201 at a dose level (i.e. 4 mg/kg dose) and no DLTs or signs of activity were observed in subjects receiving NEO-201 at the next lower dose level (i.e. 2 mg/kg dose).<br><br>Subjects will be evaluated with weekly laboratory testing for safety, and evaluated every 2 cycles for clinical response using RECIST v1.1. Correlative samples will be collected to evaluate the tumor expression of NEO-201 in archival tumor tissue, characterize the pharmacokinetics (PK), assess the immunogenicity and explore the effects of NEO-201 on immunologic correlates, including functional and phenotypic immune response and serum cytokines, chemokines and soluble factors.<br><br>Once RP2D is determined, data will be analyzed to identify potential expansion cohorts that may benefit from NEO-201 administration. In a protocol amendment, expansion cohorts will be defined, eligibility criteria described including IHC criteria for NEO-201 tumor expression, and accrual ceilings established with input from a protocol biostatistician to conduct a preliminary analysis of efficacy and to gain further experience with safety and pharmacokinetics.
Objectives:	*Primary Objective:*<br><br>To determine the safety (including dose limiting toxicities (DLT), maximal tolerated dose (MTD)) and recommended phase 2 dose (RP2D) of escalating doses of NEO-201 in adults with advanced cancer (solid tumors).<br><br>*Secondary Objective(s)*<br><br>Describe the character and incidence of Grade 1-4 toxicities based on CTCAE v5.0 that occur in adults receiving monotherapy with NEO-201.<br>Characterize the pharmacokinetics (PK) of NEO-201 monotherapy, including AUC, Cmax, Cmin using intensive sampling.<br><br>*Exploratory Objectives*<br><br>Assess immunogenicity of NEO-201 in adults with relapsed or chemo-resistant solid tumors.

	Determine in a preliminary fashion the Objective Response Rate (ORR = CR, PR, SD) as determined by RECIST v1.1 guidelines, and progression free survival, (PFS). PFS is defined as the duration of time from the first dose of NEO-201 to time of progression or death, whichever occurs first, to be assessed in both the dose-escalation cohort and dose expansion cohorts.<br><br>Describe NEO-201 IHC results in various cancer tumor types and explore the relevance in determining eligibility to receive NEO-201 mAb therapy.<br><br>Evaluate possible correlations between response rate and positivity of NEO-201 on tumor tissue samples. Immunohistochemistry (IHC) testing will be performed on pre-treatment tumor samples in order to perform exploratory correlations between level of positivity and clinical outcome.<br><br>Explore the effects of NEO-201 on immunologic correlates associated with administration of NEO-201 monoclonal antibody therapy in subjects with relapsed or refractory solid tumors, including<br>o Functional and phenotypic immune responses<br>o Serum cytokine and chemokines and soluble factors.
Population:	Adult subjects with recurrent, refractory or metastatic solid tumors, including colorectal cancer, pancreatic cancer, adenocarcinoma of the lung, squamous cell lung cancer, breast cancer, and mucinous and signet cell ovarian cancer (cancer types in which tumor samples (> 50%) historically stain positive for NEO-201 antigen).
Phase:	1
Number of Sites enrolling participants:	1
Study Duration:	The phase 1 portion of this study will be completed in 18 months. The expansion cohorts may take up to an additional 1.5 years to complete.
Participant Duration:	Subjects may continue to receive additional cycles as long as there is no unacceptable toxicity and had not developed progressive disease.

SCHEMATIC OF STUDY DESIGN

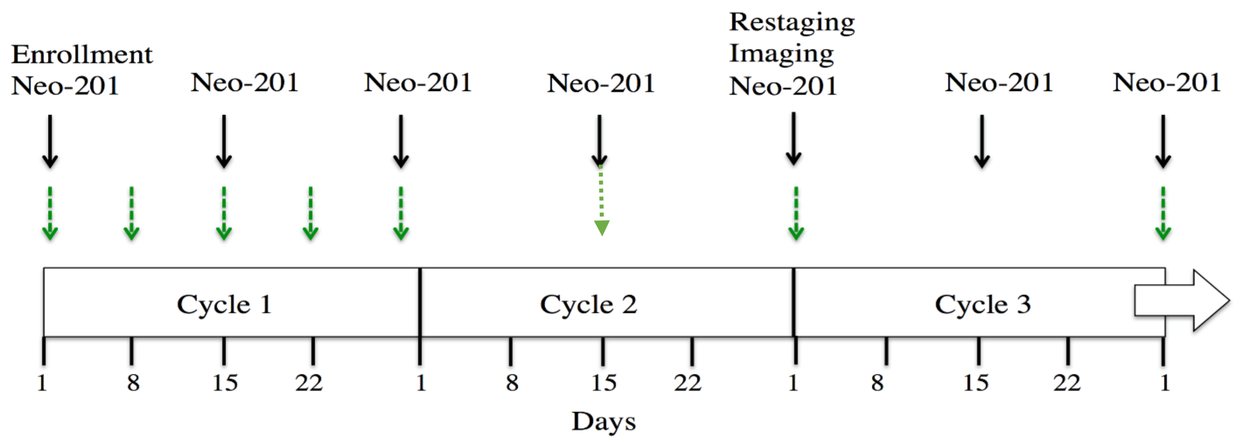

*** PK sampling: C1D1 (pre dose, immediately post dose, 1 and 4 hours post dose; C1D2 (24 hours post 1st dose); C1D4 (72 hours post 1st dose); C1D8 (7 days post 1st dose); C1D15 (pre dose, immediately post dose); C2D1 (pre dose, immediately post dose); C2D15 (pre dose, immediately post dose); C2D28 (evaluation).**

CONFIDENTIAL:
NOT TO BE DISCLOSED WITHOUT PERMISSION FROM PRECISION BIOLOGICS - FOR QUALIFIED INVESTIGATORS AND THEIR IRBS ONLY

1 INTRODUCTION: BACKGROUND INFORMATION AND SCIENTIFIC RATIONALE

1.1 BACKGROUND INFORMATION

1.1.1 Carcinoembryonic antigen

Carcinoembryonic antigen (CEA) was initially discovered as an oncofetal antigen found to be produced by epithelial tumor cells in the digestive tract. This glycoprotein is normally repressed during differentiation in the developing digestive tract and becomes overexpressed with de-differentiation seen in malignancy[1]. CEA is used as a safe, inexpensive biomarker for monitoring in subjects with colorectal cancer[2] and has been shown to be elevated in other solid tumors such as pancreatic cancer[3], lung cancer[4], ovarian cancer[5] and breast cancer[6] however the role of CEA as a prognostic marker and marker of progression is unclear and it's clinical role is frequently debated[7],[4]. High levels of CEA preoperatively have been correlated with metastasis and an overall poor prognosis[6]. Overexpression of carcinoembryonic antigen- related cell adhesion molecules (CEACAMs) have been implicated in cancer progression, inflammation, angiogenesis and metastasis. CEACAM5 is the original CEA however there are a total of 12 separate CEACAMs that play distinct roles. They all belong to the immunoglobulin (Ig) supergene family with one variable domain followed by none or up to 6 C2-like Ig domains to support the role as a heterophilic and homophilic intercellular adhesion molecule. These molecules can then be transmembrane or as in the case of CEACAM 5 and 6, through a glycosylphosphotidylinositol anchor associated with the plasma membrane[8, 9].

While CEA (CEACAM5) is used as a marker of prognosis, its role in tumor development is unclear. CEA has been shown to play a role in inhibiting differentiation and apoptosis[8]. CEA binds to CEA receptor (CEAR) promoting pro-inflammatory cytokine secretion with an increase in IL-1α and IL-1β, IL-6 and TNF-α[10]. In turn, these cytokines promote liver metastases in a nude mouse model. CEA and also CEACAM6 enhance liver metastasis in a colon cancer mouse model through direct binding to type 1 transforming growth factor-β receptor 1 (TGF- βR1) acting to attenuate TGF-β signaling, promoting tumorigenesis[11]. CEA promotes metastasis additionally by inhibiting cells that detach from the extracellular matrix-cell contacts to undergo apoptosis in a process called anoikis. CEA directly binds to TRAIL-R2 resulting in early inactivation of caspase-8[12], inactivation of caspase-9 and activation of the PI3-K/Akt survival pathway[13]. This mechanism, preventing apoptosis through anoikis, allows cancer cells to detach from their primary site, travelling through the lymphatic and circulatory system to implant and initiate tumor growth at a distant site.

CEACAM 6 is overexpressed in multiple tumor types including breast cancer and pancreatic cancer. In a retrospective study of subjects with atypical ductal hyperplasia, overexpression of CEACAM 6 is associated with progression to invasive breast cancer[14]. In a model for tamoxifen resistant breast there is an up-regulation of CEACAM 6 and enhanced migration and invasion of the cancer cells[15]. CEACAM 6 is also expressed on the surface of neutrophils and acts to regulate the adhesive activity of endothelial cells through modulating the adherence to endothelial-leukocyte adhesion molecule-1 on activated endothelial cells[16]. In pancreatic cancer cell lines,

CONFIDENTIAL:
NOT TO BE DISCLOSED WITHOUT PERMISSION FROM PRECISION BIOLOGICS - FOR QUALIFIED INVESTIGATORS AND THEIR IRBS ONLY

increased CEACAM6 expression resulted in anoikis resistance and an upregulation of PI3-K and Akt. This activation was then inhibited by CEACAM6 gene silencing[17].

Antineoplastic therapy targeting CEA has become an area if intense interest. CEA-based antitumor vaccines are currently being explored and focus on the host immune response and antitumor memory[18],[18-20]. Three different monoclonal antibodies to CEACAM 5 and CEACAM6 were used *in vitro* in pancreatic, breast and colon cancer cell lines and were found to inhibit cell migration and adhesion of tumor cells to endothelial cells. In a mouse model of intrapulmonary colonic micrometastases, treatment with the monoclonal antibodies resulted in improved survival[21]. In a phase 1 clinical trial with yttrium-90 labeled anti-CEA antibody in combination with gemcitabine in subjects with CEA positive tumors resulted in 1/36 partial response and 4/36 subjects with stable disease and a >50% reduction in baseline CEA levels[22]. In an antibody-directed enzyme prodrug phase 1 clinical trial, a recombinant fusion protein with anti-CEA and bis-iodo phenol mustard prodrug was studied and found to be safe and well tolerated. 11/28 subjects treated had stable disease[23].

1.1.2 NEO-201 (h16C3)

A monoclonal antibody was developed against a semi-purified human membrane protein preparation derived from colon cancer tissues. The protein preparation was used in previous clinical trials for use as a cancer vaccine, where it was demonstrated to be safe and efficacious[24],[25],[26]. The antibody, 16C3, was shown to react with the immunizing antigen preparation, as well as several human tumor cell lines and tissues from colorectal, pancreas, lung, and ovarian cancer subjects. 16C3 did not cross-react significantly with normal human tissues, thus representing a potential therapeutic product. The target of 16C3 was studied and shown to be related to CEACAM-5/6, a member of the carcinoembryonic antigen family of proteins, which has been shown to be associated with several cancer types. As with CEACAM-6, NEO-201 demonstrates cross reactivity with granulocytes.

Precision Biologics, Inc. engineered 16C3 to a humanized antibody (called h16C3 or NEO-201) and expressed the recombinant antibody in CHO cells. The purified h16C3 antibody was tested rigorously both *in vitro* and *in vivo* as part of pre-clinical development process. Through truncation, deletion and mutagenesis screening, the NEO-201 binding regions in CEACAM-5 and CEACAM-6 were determined and found to be unique to malignant tumor tissue types, thus evidence supporting these regions in the CEACAM-5/6 are associated with the binding of NEO-201. The following preclinical experiments provided evidences that NEO-201 binds to CEACAM5/6 isoforms:

1. **Antigen identification**: Immunoprecipitation was performed using cell lysates from LS174 and CFPAC-1 cell lines against NEO-201, the purified bands were submitted to Mass Spectrometry (MS) analysis. CEACAM5 and CEACAM6 were identified as the potential target antigens for NEO-201.
2. **Antigen transduction**: CEACAM5 and CEACAM6 genes were transduced to 293T cells separately, and Western blot experiments were performed using anti-CEACAM5 antibody, anti-CEACAM6 antibody and NEO-201. Both CEACAM5 and CEACAM6 genes transduced 293T cells can be recognized by NEO-201 in Western blot analysis.

3. **Antigen knockdown**: LS174 cell line was treated with anti-CEACAM5 siRNA and CFPAC-1 cell line was treated with anti-CEACAM6 siRNA. Western blot experiments were performed with siRNA treated cell lysates against anti-CEACAM5 antibody, anti-CEACAM6 antibody and NEO-201. The binding of NEO-201 was reduced with siRNA treated cell lysates in a dose-dependent manner.
4. **Antigen truncation**: N-terminal and C-terminal truncated CEACAM5 and CEACAM6 genes were transduced to BL21 or 293 T cell lines, Western blot and binding affinity analysis indicated the epitope region for NEO-201 binding was between aa191 and aa319 in both CEAMCAM 5 and CEACAM6 genes.
5. **Antigen Mutation**: Mutagenesis was employed to screen residues aa 197, 201-319 for CEACAM5 and CEACAM6; mutants were expressed in bacteria and mammalian expression system, and the binding activity to NEO201 was analyzed by western-blot and ELISA. The following residues are involved in NEO-201 binding to both CEACAM-5 and CEACAM-6: 236G, 259C, 269Y, 271W, 277F, 281T, 285F, 297Y, 299C, 300Q, 301A, 302H.
6. **Antigen specificity in CEACAM family**: CEACAM1, 5, 6 and 8 share the highest homology among all the members in CEACAM family. When transduced extracellular domains of these for genes to 293 T cells, NEO-201 only detected CEACAM5 and CEACAM6 by Western blot. When aligning the critical AAs that were determined in antigen mutation study among these four genes, most of them were conserved, except aa 300-302 in CEACAM8, and aa 300 and 302 in CEACAM1. When convert aa300-302 in CEACAM8, and aa300 and 302 in CEACAM1 to the aa sequences in CEACAM5/6, NEO201 could then bind to mutated CEACAM1 and CEACAM8.

An ELISA was developed to measure NEO-201 binding. Purified CEACAM-5/6 was used for the standard. Antibody-dependent cell cytotoxicity (ADCC) assay was developed to test the effector activity of NEO-201. Immunohistochemistry (IHC) protocol was developed to determine the expression of NEO-201 on human tissue.

1.1.3 Tumor Tissue Reactivity

IHC staining comparing binding specificity between commercial CEACAM 5/6 antibodies and NEO-201 are unique and there does not appear to be significant cross-reaction in healthy epithelial tissue that expresses wild type CEACAM 5/6. In adult normal tissue, CEACAM 5 is expressed in the apical border and to a lesser extent in the cytoplasm of the columnar cells of colon, and stomach, tongue, esophagus and uterine cervix. CEACAM 6 is expressed in the epithelium of similar organs but also includes granulocytes and lymphocytes. Using 32 different colon adenocarcinoma tissue samples, 28/32 (87.5%) were found to stain positive for NEO-201, while 0/31 positivity was seen in the normal adjacent tissue. In comparison, wild-type anti-CEACAM 5 stained positive in 31/32 (96.9%) colon cancer specimens and 29/32 (90.6%) of adjacent normal tissue. Wild type anti- CEACAM 6 stained positive in 31/32 (96.9%) of colon cancer specimens and 29/31 (93.6%) of adjacent normal tissue. This is consistent for pancreatic cancer and lung cancer (**Figure 1**, **Figure 2**).

CONFIDENTIAL:
NOT TO BE DISCLOSED WITHOUT PERMISSION FROM PRECISION BIOLOGICS - FOR QUALIFIED INVESTIGATORS AND THEIR IRBS ONLY

Figure 1: NEO-201 IHC of tumor tissue microarrays compared with normal tissues. Positive immuno reactivity is the percentage of core samples that stained positive for NEO-201.

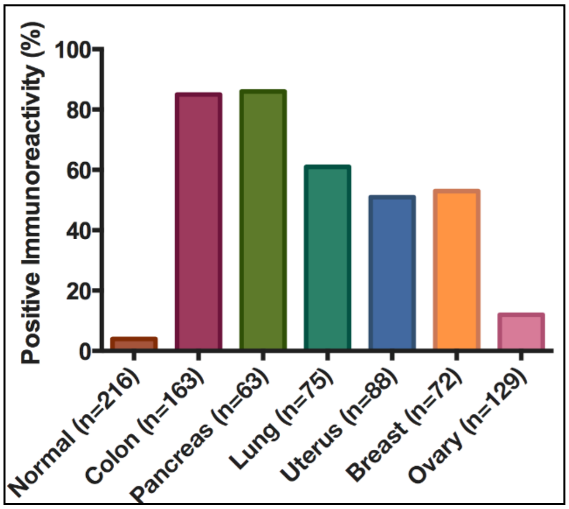

Figure 2: Immunohistochemistry peroxidase staining of NEO-201 mAb (16C3) and commercial CEACAM 5/6 antibodies in A. Colon cancer and normal colon tissue, B. Pancreas cancer and normal pancreas tissue, and C. Lung cancer and normal lung tissue.

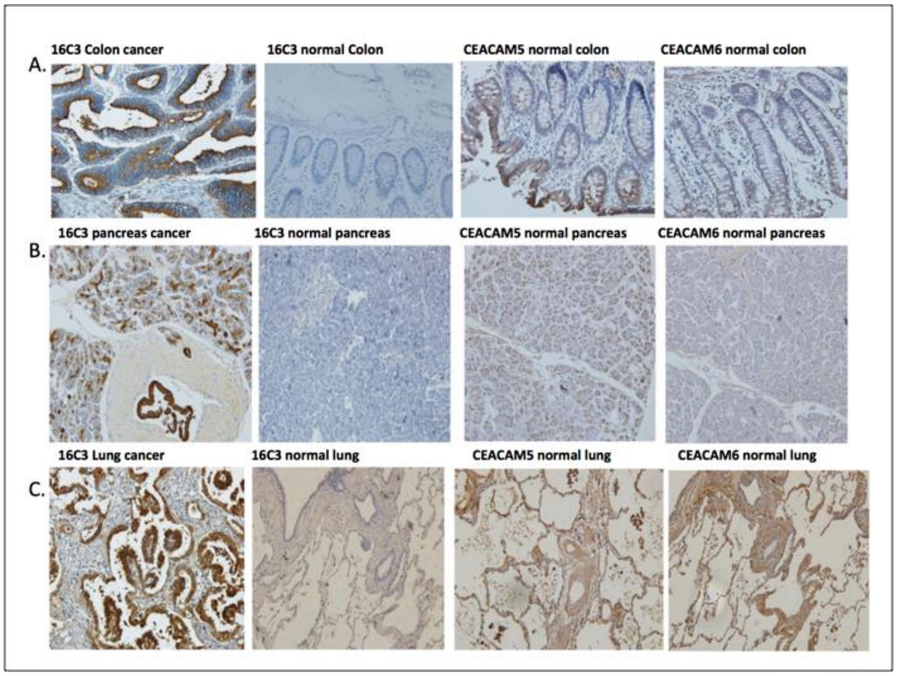

Endometrial, breast and ovarian cancer have additionally been found to have increased expression in a specific cohort of tumor samples. In endometrial cancer, 45/88 (51%) of tissue samples show reactivity through immunohistochemistry, 38/72 (53%) of breast, and although 16/129 (12%) of ovarian cancer specimens stain positive in this series, two subtypes, mucinous 15/22 (68%) and signet cell 2/2 (100%) ovarian cancers, shows significant reactivity (>50%) in an IHC of ovarian cancer tissue arrays representing over 600 samples (**Figure 3**).

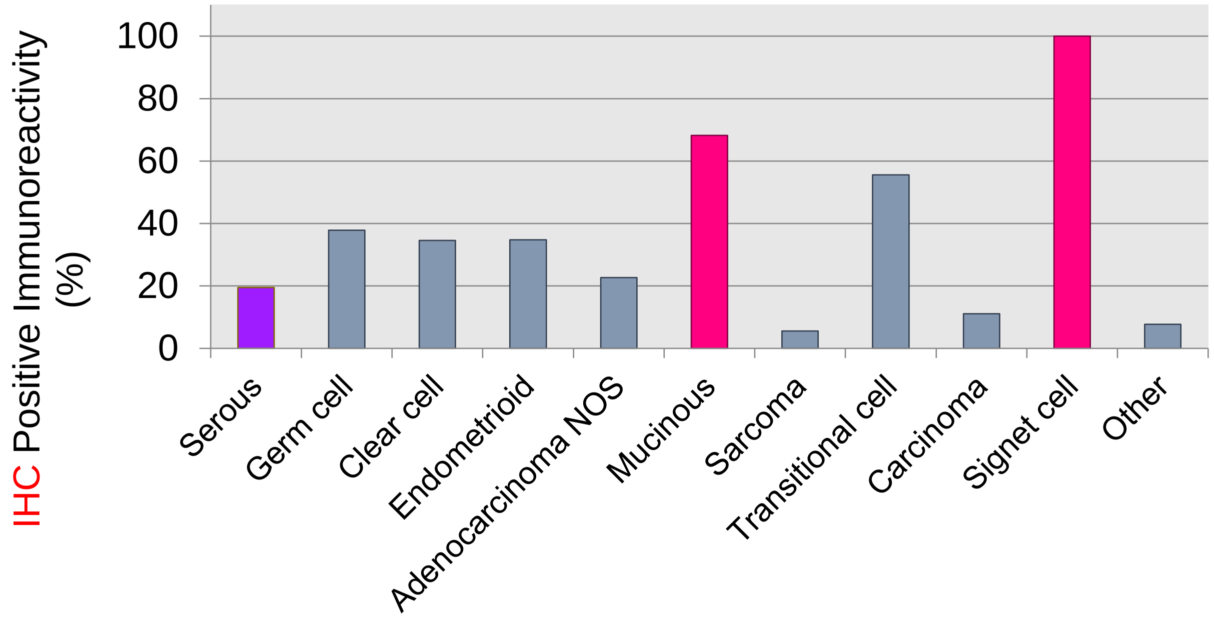

Figure 3: Immunoreactivity to NEO-201 in over 600 samples for ovarian cancer from Roswell Park Cancer Institute

The expression of NEO-201 antigen on lung cancer tissues was investigated and the results are shown in **Table 1.**

Table 1: NEO-201 IHC Profile: Results from Lung Cancer Microarray

**Lung Cancer Type**	**Positive#/Total Case (% Reactivity)**
**Lung adenocarcinoma**	**27/34 (79.4%)**
**Lung Squamous Cell Cancer**	**18/34 (52.9%)**
**NOS non-small cell lung cancer**	**0/4 (0%)**
**Bronchi alveolar Cancer**	**1/2 (50%)**
**Large cell neuroendocrine Cancer**	**0/1 (0%)**
**Lung Cancer (Total)**	**46/75 (61.3%)**

In addition a GLP normal human tissue cross reactivity (TCR) study was conducted at Charles River Laboratories, Inc. as describes in section **1.1.7.2.1**

CONFIDENTIAL:
NOT TO BE DISCLOSED WITHOUT PERMISSION FROM PRECISION BIOLOGICS - FOR QUALIFIED INVESTIGATORS AND THEIR IRBS ONLY

1.1.4 Antitumor Activity

NEO-201 shows antitumor efficacy *in vivo* in a mouse model with xenografts using CFPAC-1 (human pancreatic adenocarcinoma) cells. 100ug or 250ug of NEO-201 was injected intraperitoneally on days 13, 17 and 20 following CFPAC-1 injection (**Figure 5:** Humanized 16C3 anti-tumor efficacy results in CFPAC-1 tumor-bearing mice, followed by intraperitoneal injection of peripheral blood mononuclear cells (PBMCs) on days 14, 18 and 21. In mice that received both the PBMCs and NEO-201 there was a significant reduction in tumor volume (**Figure 5** and **Figure 6)**. In mice only receiving NEO-201, there was a reduction in tumor volume that did not reach statistical significance. This suggests that there is antibody dependent cellular cytotoxicity (ADCC) with both immune and direct tumor killing. There were no toxicities in the mice based on total body weight.

Figure 4: NEO-201 (Humanized 16C3 (h16C3) anti-tumor efficacy study in the CFPAC-1 tumor xenograft model

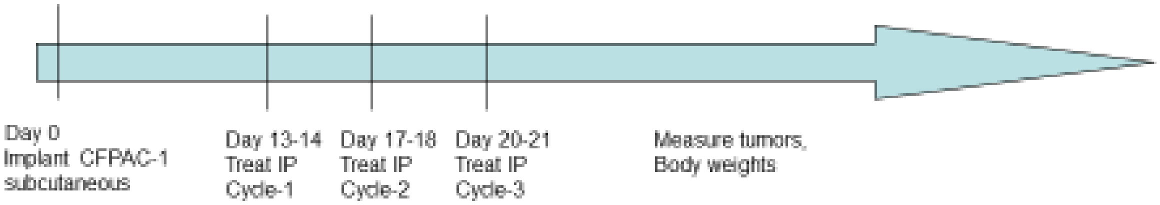

Group (n=10)	Antibody, dose	PBMC (NK cells)
1	Saline	~1x10^{7}
2	Human IgG, 250ug	~1x10^{7}
3	h16C3, 100ug	~1x10^{7}
4	h16C3, 250ug	No PBMC
5	h16C3, 250ug	~1x10^{7}

CONFIDENTIAL:
NOT TO BE DISCLOSED WITHOUT PERMISSION FROM PRECISION BIOLOGICS - FOR QUALIFIED INVESTIGATORS AND THEIR IRBS ONLY

Figure 5: Humanized 16C3 anti-tumor efficacy results in CFPAC-1 tumor-bearing mice

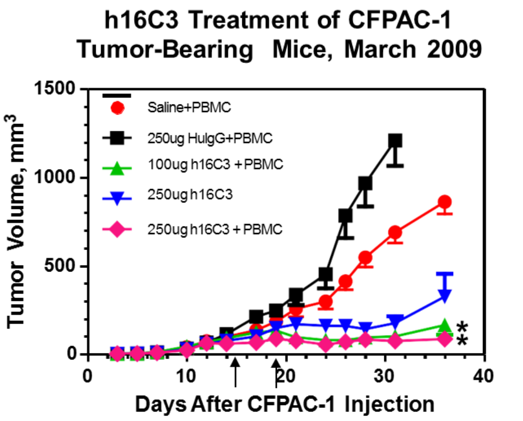

*, indicates p<0.05 vs. saline group

h16C3 injected intraperitoneally on Days 13, 17, 20
PBMC injected intraperitoneally on Days 14, 18, 21

<u>On Day 36:</u>
100ug h16C3+PBMC, 1/10 mice tumor-free
250ug h16C3+PBMC, 4/10 mice tumor-free

Figure 6: Humanized 16C3 anti-tumor efficacy results

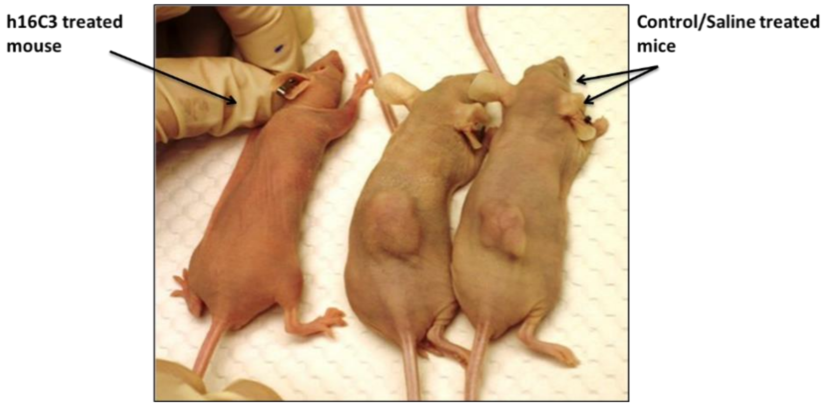

Preclinical studies in cancer cell lines treated with NEO-201 demonstrate antibody dependent cell-mediated cytotoxicity (ADCC) (**Figure 7 A and B**). In addition, NEO-201 has been demonstrated killing of certain cell lines by complement dependent cytotoxicity (CDC) (**Figure 7 C).** Additional mechanisms of tumor killing by NEO-201 remain under investigation.

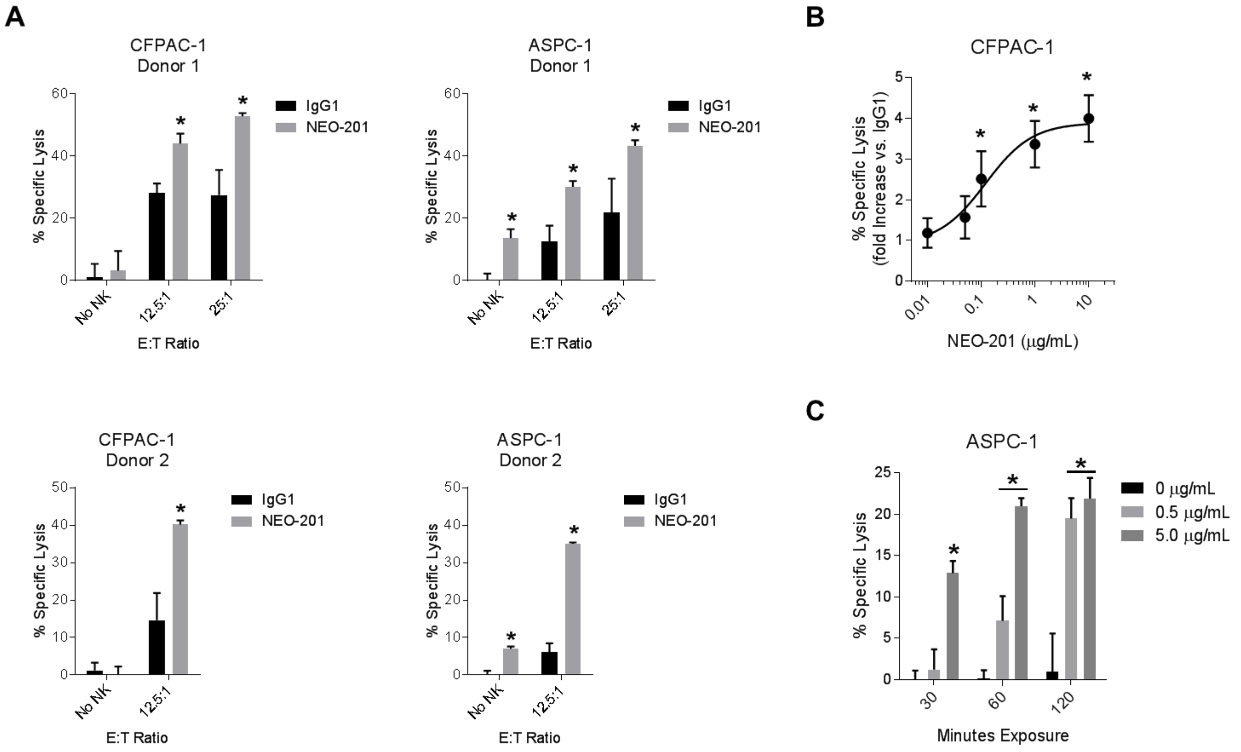

Figure 7: ADCC and CDC Killing in Pancreatic Cancer Cell Lines

(A) ADCC activity using CFPAC-1 or ASPC-1 cells as target cells. Cells were treated with 10µg/mL of NEO-201 or human IgG1 (negative control). Purified NK cells from two healthy donors were used as effector cells at the indicated E:T ratios. *, statistically significant ($p < 0.05$) by T-test. (**B**) ADCC assay using CFPAC-1 cells treated with increasing doses of NEO-201. NK cells isolated from a healthy donor were used as effector cells at an E:T ratio of 12.5:1. The graph depicts the fold increase in % specific lysis of NEO-201-treated tumor cells versus that of control cells treated with 10µg/mL human IgG1. *, statistically significant ($p < 0.05$) by T-test. (**C**) CDC assay using ASPC-1 cells treated with rabbit complement (1:8 dilution) and the indicated doses of NEO-201 for the indicated durations. *, statistically significant ($p < 0.05$) by T-test.

1.1.5 Biodistribution of [^{125}I] labeled h16C3 (NEO-201)

The biodistribution of humanized recombinant mouse monoclonal antibody h16C3 (NEO-201) was examined in male and female nude mice (Hsd: athymic nude-Foxn1^{nu}) bearing CFPAC-1 pancreatic cell tumors (injected subcutaneously in the flank with a 200µL suspension of 4.0×10^6 CFPAC-1 cells) (Comparative Biosciences, Sunnyvale, CA). On day 14 after engraftment, 20 µCi of [^{125}I] labeled h16C3 (NEO-201) antibody was injected into the tail vein of the nude mice and groups of mice (4 of each sex) were necropsied 1, 2, 4, and 7 days later. Blood, tumor, and the following organs were removed, weighed and counted in their entirety: lungs, kidneys (2), liver, spleen, pancreas, intestines, and stomach. Radioactivity measurements (cpm) were measured using a gamma counter, and were corrected for decay to the day of injection and expressed as cpm/g tissue. Data for each mouse was first calculated as cpm/mg tissue, and then tissue cpm

values were normalized relative to blood cpm values. Figure 8 and Figure 9 depicts mean ± SD, n = 4.

Cpm/g in organs and blood were highest the day after injection and decreased with time. Cpm/g tumor in male mice were equivalent on days 1 and 2, and decreased thereafter. In females, cpm/g tumor remained elevated on days 1, 2, and 4 and decreased on day 7. Mean cpm/g tumor normalized for cpm in blood (T:B), increased with time in both male and female mice reaching levels 12.6 (females) and 14.6 (males) times higher than those in blood on day 7.

Figure 8: Tissue Distribution (cpm/g) of [^{125}I]h16C3 Antibody in Male Nude Mice with CFPAC-1 Tumors

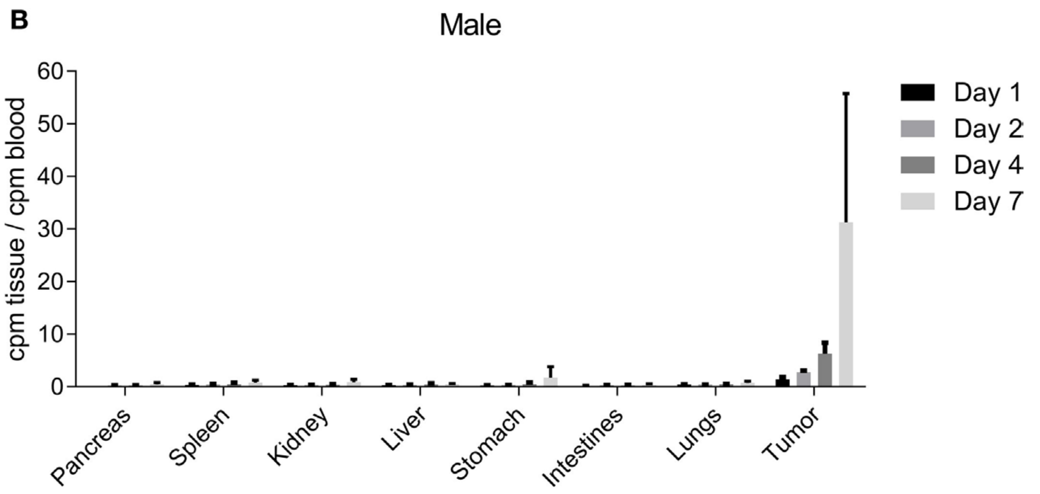

Figure 9: Tissue Distribution (cpm/g) of [^{125}I]h16C3 Antibody in Female Nude Mice with CFPAC-1 Tumors

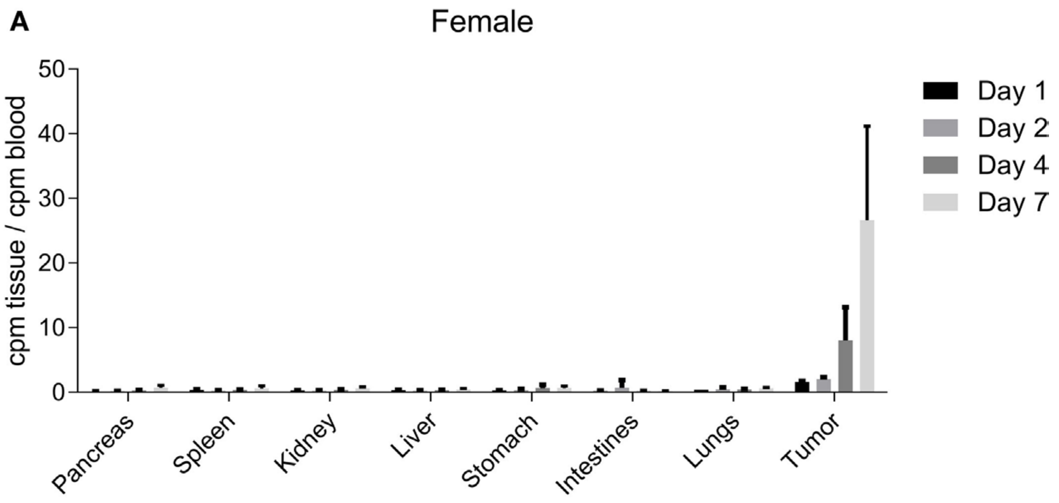

As shown above, when normalized for blood levels of radioactivity, both male and female nude mice showed preferential accumulation of [^{125} I] labeled h16C3 (NEO-201) antibody in tumors, with higher levels in tumor than any other tissue at all time points, reaching 12-14 times higher than those of blood samples on necropsy on day 7. Qualitatively similar results were obtained for male and female mice.

1.1.6 Manufacturing

Precision Biologics, Inc. contracted Catalent Pharma Solutions, LLC to select the best producing NEO-201 clone, perform cell line development, manufacture a master cell bank and perform upstream process development and harvest and purify a scale-down run of the NEO-201 selected clone. One engineering run was produced for IND enabling studies, followed by cGMP manufacturing of NEO-201, which is being submitted to the FDA on IND #134656.

1.1.7 Preclinical pharmacology and toxicology

1.1.7.1 Selection of an animal model

NEO-201 has been shown by IHC to bind to the cell surface of a wide variety of human epithelial carcinomas but rarely cross reacts to surrounding normal tissue. As observed with CEACAM6 related antibodies, NEO-201 has shown cross reactivity with human granulocyte populations. We performed two studies to determine which animal species would be most relevant for preclinical Pharmacology/Toxicology studies based on their similarity to humans.

An animal IHC study was performed using gastro-intestinal tissues (esophagus, stomach, colon and rectum) from normal C57BL/6 and BALB/c mice (1 male for each strain), cynomolgus monkey (1 male), mini pig (1 male), cow (1 male), rat (11 GI tissues from 1 male) and processed for IHC. Sections were stained with biotinylated NEO-201 antibodies (according to SOP AP-003: NEO-201 Immunohistochemistry (IHC)); biotinylated human IgG1 kappa (isotype control); or pan CK (positive tissue control). As a positive (antibody) control, known positive reactors (human colon carcinoma and pancreatic adenocarcinoma previously demonstrated to be stained by NEO-201 antibody) were also processed and stained by IHC. Staining of the target cells in the human colon carcinoma and pancreatic adenocarcinoma (positive reactors) was observed with the biotinylated NEO-201 antibody but not with the isotype control, thus validating the IHC detection of this antigen. Similarly, staining was observed with NEO-201 antibody in esophagus and colon of cynomolgus monkey and mice. NEO-201 tissue cross reactivity was not detected in the cow, rat or mini pig gastro-intestinal tissues.

To further characterize the relevant species, flow cytometry was performed using PBMCs from human, monkey and mouse, to stain granulocyte marker and NEO-201 antibody. The granulocytes from both human and monkey PBMCs stained similarly positive with NEO-201. However mouse granulocytes did not stain with NEO-201. Thus, the use of the cynomolgus monkey is the most relevant species for animal toxicology studies of NEO-201 antibody.

1.1.7.2 Preclinical toxicology

1.1.7.2.1 Human Tissue Cross Reactivity (TCR) Study

CONFIDENTIAL:
NOT TO BE DISCLOSED WITHOUT PERMISSION FROM PRECISION BIOLOGICS - FOR QUALIFIED INVESTIGATORS AND THEIR IRBS ONLY

A GLP normal human tissue cross reactivity (TCR) study was conducted at Charles River Laboratories, Inc. To determine the potential cross reactivity of NEO-201 with human tissues, biotinylated NEO-201 was applied to cryosections of normal human tissues (3 donors per tissue) at two concentrations (5 and 1 µg/mL). The test article was substituted with a biotinylated monoclonal human IgG1 antibody as the control article. NEO-201 produced strong to intense membrane and cytoplasmic staining of frequent positive control pancreatic CFPAC-1 cells at both staining concentrations, and produced moderate to intense membrane and cytoplasmic staining of frequent positive control OV-90 cells at the higher concentration with a reduction in staining frequency at the lower concentration. NEO-201 did not specifically react with the negative control OVCAR8 cells at both staining concentration, and the control article, HuIgG1 did not specifically react with either the positive or negative control materials. There also was no staining of the assay control slides. Staining with NEO-201 was observed in the human tissue panel of plasma membrane and cytoplasmic elements of leukocytes and hematopoietic cells (including mononuclear and granulocyte precursors) in the bone marrow and in most human tissues. There was some instances of staining of epithelium (GI, lung, pancreas, prostate, skin, tonsil and uterus) and some staining of cytoplasmic elements of the epithelium in the breast, colon, salivary gland (acini, ducts), thyroid, ureter, and uterus (cervix [internal ostium, endocervical glands]); endothelium in the brain (cerebellum), breast, colon, GI tract (small intestine), parathyroid, spinal cord, and thyroid; spindle cells in the pituitary; microglia in the spinal cord. There was also some staining observed in the extracellular material in the breast (intraductal secretory material), lung (in alveolar and intravascular spaces), prostate (secretory material in glands and ducts), and thyroid (colloid).

The staining of leukocytes, bone marrow hematopoietic cells, and various epithelia with NEO-201 is consistent with the reported expression of CEACAM5/6 proteins[27],[28],[21],[29],[30],[31],[32],[33],[34],[35],[36],[37],[38],[39],[40],[41],[42] which are similar to the target antigen for NEO-201. Additionally, cytoplasmic staining only with NEO-201 was observed in endothelium, spindle cells, and microglia in the human tissues, although it is unlikely that the cytoplasmic compartment would be accessible to the test article *in vivo*. Staining of extracellular material with NEO-201 was also present in a few human tissues, which may represent binding to a secreted form of the protein.

1.1.7.2.2 Single Dose Toxicology Study

A single-dose toxicity study was conducted in purpose-bred cynomolgus monkeys to test NEO-201 for toxicity and toxicokinetics after a single dose of the monoclonal antibody NEO-201(SNBL USA, Ltd. Everett, WA). Eight (8) male and 8 female animals (2 animals/sex/group) were dosed by slow intravenous (IV) infusion after 14 days of acclimation at dose levels of 5 mg/kg (Group 2), 20 mg/kg (Group 3) or 49 mg/kg (Group 4) on Day 1 (Group 1 received 0 mg/kg NEO-201). Observations and examinations included clinical observations (twice daily), food consumption (once daily), body weight (Days 7 and 14), urine and blood evaluations (incl. urinalysis, hematology, coagulation tests, serum chemistry, and toxicokinetics). Pharmacokinetic samples were drawn in all animals in Groups 2 – 4 at the following time points: pre-dose, 10 minutes, 1, 2, 4, 6, 24, 48, 72, 96, 168, and 336 hours.

From our previous experience using a chimeric monoclonal antibody (NEO-102 [ensituximab, NPC-1C]), we deduced a possible starting dose of 1 mg/kg in humans. A comparable starting dose level in cynomolgus monkeys was established as 5 mg/kg (equivalent to human dose of 1.6 mg/kg). The 2nd dose level was almost 3-fold increase with the dose administered at 20 mg/kg. The high dose administered was established at 49 mg/kg because 49 mg/mL is the highest dose that can be given to the monkeys with the current drug concentration of 9.76 mg/mL (volume limitation in monkeys is 5 mL/kg, with an average 3 kg weight of the animal).

The only significant finding during the 14 day acclimation period was bruising of the hindlimb(s) in several animals and hair loss prior to infusion of the test article. According to the animal specialists at SNBL USA where the study was conducted, this is a common finding during the acclimation period. After infusion of NEO-201 on Day 0, there were no significant changes in body weight, food consumption, or laboratory testing, except for changes in neutrophil counts. Neutrophil alterations were initially detected at Day 2, for animals in all treatment groups (5, 20 and 49 mg/kg). The decreases were of varying magnitudes, ranging from mild to marked, and a clear dose response was not evident. For the majority of animals, this was a transient finding, as improvements were typically noted by Day 8 and neutrophil decreases had resolved by Day 15 (although neutrophil values generally did not return to acclimation levels). For two Group 3 males (Animals 10 and 12), one Group 4 female (Animal 15) and one Group 4 male (Animal 16), the decreases persisted through to Day 15, with minimal to mild improvements in neutrophil counts noted.

CEACAM6 has been found to be expressed on granulocytes[40], macrophages and monocytes[43]. Hence the observed decrease in neutrophils in the treated animals versus the control group may reflect an on-target side effect of the NEO-201. The laboratory monitoring of the repeated dose toxicity studies will evaluate this effect more closely and provide additional details for the design of the clinical study. Vigilant monitoring of CBC and differential is included in the proposed monitoring of this clinical trial to ensure the safety of NEO-201 administration in humans during this first-in-human study.

Preclinical pharmacokinetics

Preclinical pharmacokinetics were conducted for two weeks after administration of a single intravenous dose of NEO-201 using the cynomolgus monkeys to evaluate responses and toxicokinetics (TK). Preclinical PKs will also be conducted in the 4-week repeat-dose toxicity study.

- Materials and Methods

TK data evaluation of NEO-201 in serum of cynomolgus monkeys was performed for each individual animal profile using serum concentration and nominal time data. The nominal IV doses administered to animals in Group 2, 3, and 4 were 5, 20, and 49 mg/kg, respectively. The nominal blood collection times for TK data analysis were: 0 (infusion end time) and at 0.167, 1, 2, 4, 6, 24, 48, 72, 96, 168, and 336 hours after infusion completion.

Analysis of serum samples for NEO-201 was performed by the Sponsor using a qualified ELISA method. TK analysis was performed using noncompartmental methods with validated Phoenix WinNonlin, version 6.1 software (Pharsight Corporation) and data from Groups 2 - 4.

- Results and Conclusions

Toxicokinetic results are summarized in **Table 2**: Mean TK results.

Table 2: Mean TK results

Group	HL (hr)	Tmax (hr)	Cmax (µg/mL)	Cmax/D (µg/mL/mg)	AUCinf (hr*µg/mL)	AUCinf/D (hr*µg/mL/mg)	CL (mL/hr)	Vz (mL)
2 (5 mg/kg)	46.2	0.584	138	11.4	8,220	680	1.54	103
3 (20 mg/kg)	167	0.167	579	11.2	70,100	1,360	0.746	179
4 (49 mg/kg)	170	0.167	1,470	11.8	157,000	1,260	0.830	191

When administered as a 30-minute IV infusion, quantifiable and dose-dependent serum concentrations of NEO-201 were observed through the last collection time point, 14 days post-dose. There was no difference between dose groups in T_{max} (0.167 or 0.584 hour). Over the dose range evaluated, peak (C_{max}) exposure was dose proportional; total (AUC) exposure was greater than dose proportional at the lowest doses and approximately proportional from 20 mg/kg to 49 mg/kg. Differences in exposure at the lowest dose were attributed to an approximately 2-fold greater mean clearance (CL) and lesser volume of distribution (V_Z). Mean half-life (HL) was 167 (20 mg/kg) or 170 (49 mg/kg) hours at the higher doses, approximately 3.7-fold greater than at the 5 mg/kg dose (46.2 hours). Sex-differences were not observed.

1.1.7.2.3 Multiple Dose Toxicology Study

A 5 week toxicity GLP study with a 6 week recovery period has been conducted in purpose-bred cynomolgus monkeys (SNBL USA, Ltd., Everett, WA). NEO-201 was administered to 3 groups of cynomolgus monkeys (group 1 (control group), group 2 (5 mg/kg of 1 mg/mL NEO-201), group 3 (20 mg/kg of 4 mg/mL NEO-201) and group 4 (50 mg/kg of 10 mg/mL NEO-201) on Days 1, 8, 15, 22 and 29, followed by a recovery period. There were 5 animals/sex/group for the control and high dose groups and 3 animals/sex/group for the low and middle dose groups. All animals underwent 14 days of acclimation prior to dosing. Terminal necropsy was performed on Day 30 and recovery necropsy was performed on Day 71. Monitoring parameters included clinical observations, body weight, ECG, ophthalmology, urinalysis and blood laboratory analyses, and toxicokinetics (samples on Day 1 and Day 29 pre-dose, 10 min, 2, 6, 24, 48, 72, and 168 hours post dose, as well as Day 15 and 22 pre-dose, and Days 9, 16, 23 post-dose, and on Days 43, 50, 57, 64, and 71). Final results are included in the IND submitted to the FDA.

Findings were similar to the single dose study of NEO-201 in that several animals in all 3 treatment groups experienced drops in neutrophils. There were also changes in red blood cell counts, hemoglobin and hematocrit, but the trend was less significant than that seen in neutrophils. The neutrophil changes in males versus female monkeys can be seen in **Figures 10**. After the initial precipitous drop in neutrophils, there was a slow, and fairly consistent rise in neutrophils after the first dose.

Figures 10: A. Neutrophil Counts in Male and B. Female Cynomolgous Monkeys after NEO-201

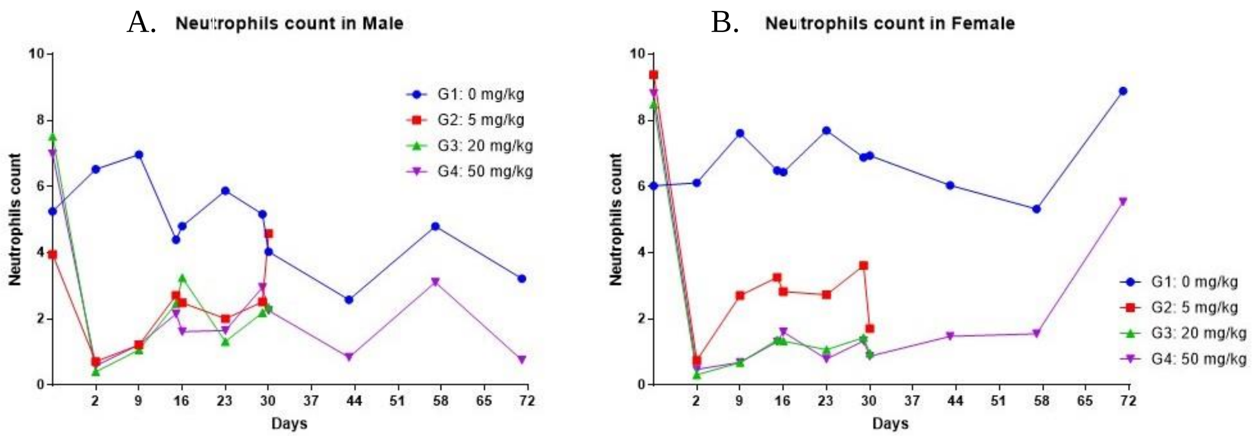

There were no trends in clinical changes in animals treated at any dose level. The organ weights at necropsy were generally unremarkable. One animal (animal 4505) in the high dose group experienced liquid/soft feces on Day 27, and slightly decreased body weight; animal 4504 developed an infection in the tail region. Both animals were treated symptomatically and recovered without sequellae.

Pharmacokinetic studies results can be found in the IND for this study. The PK and toxicology findings appear to indicate that NEO-201 can be given safely in the doses proposed in this study to support use of this neoantigen in humans.

1.1.8 Correlative Studies

1.1.8.1 Cellular Immune Monitoring Assays

1.1.8.1.1 Cell surface marker Analysis

Regulatory Tcells (Treg) and myeloid derived suppressor cells (MDSCs) have been shown to suppress immune function allowing for continued tumor development and growth. Preliminary laboratory data indicates that NEO-201 can bind to both granulocytes, as well as MDSCs, and may play a role in reducing suppressor cell levels and function. Therefore, we plan to analyze the effect of NEO-201 on levels of regulatory Tcells, Treg-to-lymphocyte ratio, gMDSC and mMDSCs to determine if changes correlate with clinical outcome. Other markers that may be of prognostic value including neutrophil-to-lymphocyte ratio will also be tested.

1.1.8.1.2 Functional Analysis

In addition to evaluating the above markers, functional assays will be performed to determine whether changes in levels alter immune function *in vitro*. These assays of function include proliferation, as well as Treg and MDSCs suppression assays (CSFE assay).

The phenotype of the CD16 receptor on NK cells has been shown to effect the killing activity through antibody dependent cell mediated cytotoxicity (ADCC), with the V/V phenotype correlating with a high affinity receptor and better killing. Conversely the F/F phenotype has been

correlated with weak affinity and poor killing activity. We plan to evaluate the phenotypes of subjects' NK cells and perform functional testing of ADCC to conduct further testing of this immunologic observation.

Finally, we will analyze immune response gene expression and correlate the results with clinical outcome in subject using NanoString nCounter evaluation.

1.1.8.1.3 Humoral immune monitoring assays:

Preclinical testing of NEO-201 indicates this novel neoantigen elicits humoral responses in addition to the cellular immune changes. These include the possibility of stimulating both Pro-inflammatory and anti-inflammatory cytokines and chemokine. Hence samples will be collected to evaluate for specific humoral changes both pre and post therapy.

1.1.9 Phase 1 dose escalation disease populations

Based on tissue reactivity studies, the dose escalation phase of this trial will be open to the following tumor types in which reactivity occurs in the majority (> 50%) samples including colon cancer (85%), pancreatic cancer (86%), adenocarcinoma of the lung (79%), squamous cell lung cancer (53%), breast cancer (53%) and mucinous and signet cell ovarian cancer (>50%).

1.1.9.1 Rationale for selection

Targeted immunotherapy is currently the focus of a significant portion of anticancer drug development. The development of targeted therapies requires not only the identification of functional targets, but also a means by which the target can be detected on cancer tumor and consistently measured, as well as determining the level of expression which indicates potential for targeted therapy benefit. One example of variable acceptable measures of expression was recently approved by the U.S. Food and Drug Administration (FDA), KEYTRUDA®, which is indicated for subjects with metastatic non-small cell lung cancer (mNSCLC) whose tumors have high PD-1 expression (tumor proportion score [TPS] ≥ 50%), and is also indicated for subjects with mNSCLC who have disease that has progressed on or after platinum-based therapy if the tumor expresses PD-L1 of TPS ≥ 1%. TPS is defined as the percentage of viable tumor cells showing partial or complete staining (≥ 1+).

Immunohistochemistry (IHC) testing, although widely used, and approved in some instances for drug selection, as in HER2 IHC for Herceptin therapy, faces several challenges related to reproducibility, standardization, and determination of a reasonable cut off to predict benefit. Patel and Kurzrock described significant ranges of expression of PD-L1 when IHC was studied in various tumor types, including melanoma which ranged from 38%[44] in one series to 100%[45] in another series[46], or NSCLC which ranged from 49%[47] to 95%[45].

There is no clear definition of "positive" tumor staining. The FDA approved Herceptin® (trastuzumab) for HER2-overexpressing metastatic breast cancer[48], but 'overexpression' is not defined in the package insert. Several studies reported in the package insert in subjects with breast cancer and metastatic gastric cancer indicate that overexpression was defined in the referenced study as 3+ by IHC, but do not indicate the % of tumor cells that must stain at this level. One study referenced accepted overexpression eligibility as 2+ or 3+. The European Commission

specifically defines Herceptin treatment as indicated for HER2 overexpression with IHC intensity of 3+, but again does not indicate the percent of cells that must stain at this intensity.

During the phase 1 portion of this study, subjects with colorectal cancer, pancreatic cancer, adenocarcinoma of the lung, squamous cell lung cancer, breast cancer, and mucinous and signet cell ovarian cancer will be eligible to participate based on the preliminary IHC testing that has been conducted showing that the majority of subjects with these types of cancer have some NEO-201 expression (>50% of subjects with this tumor-type express NEO-201 at ≥ 2+ intensity in ≥ 10% of tumor cells). Subjects will be required to have archived tissue available (at least 10 unstained slides or tissue block) or have easily accessible tumor which can be safely biopsied upon enrollment; tumor from enrolled subjects will be tested by IHC in an NCI research laboratory (CLIA laboratory, Frederick MD; pathologist Steven Hewitt, M.D) to further explore IHC expression, relationship to benefit and gain further experience with the IHC procedure. Once the RP2D is determined, eligibility criteria will be modified to include 1) tumor types for enrollment in the expansion cohorts, 2) modifications to the IHC SOP, and 3) IHC determinations for enrollment. Expansion cohorts will be added to the study with a protocol amendment based on this analysis.

1.2 RATIONALE

The primary purpose of this first in human targeted phase 1 open-label study with NEO-201 in subjects with advanced solid tumors is to determine the safety of NEO-201 and select a dose for phase 2 clinical trials. Initially subjects will be enrolled based on experience with NEO-201 expression by immunohistochemistry (IHC) of the CEA-CAM variant in the subject population. Tissue specimens from subjects screened during the dose escalation cohort will be batched for NEO-201 IHC analysis to provide essential information for identifying the eligibility criteria in the expansion cohorts.

Escalating doses of NEO-201 will be administered in a standard 3+3 dose escalation design. The starting dose of 1 mg/kg was chosen based upon the safety analysis in the non-human primate multi-dose toxicity study. Given the lack of no observed adverse event level (NOAEL), the dosing (1, 2, 4, 6 mg/kg) was calculated based on the safety observed in the highest dose cohort (50 mg/kg) in the multi-dose animal study[49]. Once the RP2D in humans is defined, expansion cohorts will be identified and IHC expression eligibility requirements will be defined in a protocol amendment to gain further information regarding toxicity, pharmacokinetics, and clinical activity. Exploratory analysis of functional and phenotypic immune processes, serum cytokine and chemokines and soluble factors, and analysis of changes in the tumor microenvironment (optional) will assist in better defining the mechanism of action and activity of this novel neoantigen.

1.3 POTENTIAL RISKS AND BENEFITS

1.3.1 Known Potential Risks

This study involves clinical research with an experimental monoclonal antibody designed to generate an anti-tumor response in subjects that have tumors that have a probability of expressing the target for the NEO-201 antigen. Subjects will undergo antibody treatment every 2 weeks while

on study and will be asked to have blood drawn to study how this drug works in subjects with cancer.

This is the ‘first-in human’ study of NEO-201. Preclinical studies including human tissue cross reactivity have not identified any specific risks to date, except the NEO-201 impact on granulocytes/neutrophils and possibly hemoglobin. Close monitoring of complete blood counts will be conducted in all subjects. Subjects who develop neutrophil decrease will be treated with growth factor. Subjects are at risk of febrile neutropenia and infections given the incidence of neutropenia. In addition, administration of monoclonal antibodies including NEO-201 can cause infusion reactions, allergic or anaphylactic reactions. Subjects will be administered pre-infusion medications to reduce this risk, and the infusion rate will be modified to tolerance. Also, the antibodies may bind specifically or non-specifically to antigens expressed on non-tumor tissue which could ensue in an immune reaction.

The target population in this study, individuals with cancer who have undergone multiple prior therapies with chemotherapy, radiation and immune modifiers, are at increased risk of infections often due to low bone marrow reserve and altered anatomy. NEO-201 has a known side effect of causing significant neutropenia. Given the high risk of infection based on these predisposing factors, all potential subjects will be screened for recent infections or infectious exposures and have an infectious disease consult. Any indications of a higher risk for infection or presence of indolent infectious sources will be thoroughly evaluated during screening. Subjects initiating study therapy may be asked undergo hospitalization at NIH during the first cycle if grade 3 or greater neutropenia develops, until ANC > 1000 mm^3. Treatment plans to reduce the risk of infection during neutropenia will be generated under the guidance of Infectious Disease Consult.

Toxicities reported with the use of the ‘first in class’ monoclonal antibody, NEO-102 (Ensituximab), derived from the same colon cancer tissues[26],[24],[25] in 83 subjects with colorectal or pancreatic cancer included grade 1 / 2 fatigue, anemia, nausea, vomiting, decreased appetite, flushing, hyperbilirubinemia, hemolysis, allergic reactions, chills, constipation, diarrhea, asthenia, dyspnea, and weight loss. All other grade 1-2 toxicities occurred in 1 or 2 subjects each. Grade 3-4 toxicities included anemia (8%), fatigue (5%), hyperbilirubinemia (4%), hemolysis (2%), and nausea, vomiting, headache, and hypoxia (1% each). Only 1 subject (out of 83) discontinued study drug due to toxicity and only 1 subject required a dose modification due to toxicity.

The potential toxicities extrapolated from preclinical toxicology studies in mice and monkeys were limited to neutropenia and anemia.

1.3.2 Known Potential Benefits

As this is the ‘first-in human’ study of NEO-201, there is no direct data to demonstrate benefit. Preclinical testing, as described in section **1.1.4**, demonstrated antitumor efficacy *in vivo* in a mouse model using human pancreatic adenocarcinoma cells. Laboratory testing showed at least two mechanisms of action, ADCC and CDC. Additional mechanisms of tumor killing by NEO-201 remain under investigation. Although the benefit is unknown, subjects participating in this trial will have recurred after or not responded to all known available curative options and clinical response will be closely monitored during treatment on this study.

CONFIDENTIAL:
NOT TO BE DISCLOSED WITHOUT PERMISSION FROM PRECISION BIOLOGICS - FOR QUALIFIED INVESTIGATORS AND THEIR IRBS ONLY

1.3.3 Risks/Benefits Analysis

This study involves clinical research with an experimental monoclonal antibody designed to generate an anti-tumor response in subjects that have tumors that have a probability of expressing the target for the NEO-201 antigen. Subjects will undergo antibody treatment every 2 weeks while on study and will be asked to have blood drawn to study how this drug works in subjects with cancer. Alternative treatments for subjects will likely include additional chemotherapy, other clinical trials, or supportive care. The potential side effects of the antibody therapy are outlined elsewhere in the protocol. Whether the antibody will have any clinical effect is unknown; therefore, benefit cannot be promised nor can the chance of benefit be accurately predicted. Subjects' participation in this study is voluntary and refusal will not result in penalty or loss of benefit to which subject is otherwise entitled.

2 OBJECTIVES AND PURPOSE

2.1 Primary Objective:

- ***Phase I:*** To determine the safety (including dose limiting toxicities (DLT), maximal tolerated dose (MTD)) and recommended phase 2 dose (RP2D) of escalating doses of NEO-201 in adults with advanced cancer (solid tumors).

2.2 Secondary Objective(s)

- Describe the character and incidence of Grade 1-4 toxicities based on CTCAE v5.0 that occur in adults receiving monotherapy with NEO-201.
- Characterize the pharmacokinetics (PK) of NEO-201 monotherapy, including AUC, Cmax, Cmin using intensive sampling.

2.3 Exploratory Objectives

- Assess immunogenicity of NEO-201 in adults with relapsed or chemo-resistant solid tumors.
- Determine in a preliminary fashion the Objective Response Rate (ORR = CR, PR, SD) as determined by RECIST v1.1 guidelines, and progression free survival, (PFS). PFS is defined as the duration of time from the first dose of NEO-201 to time of progression or death, whichever occurs first, to be assessed in both the dose-escalation cohort and dose expansion cohorts.
- Describe NEO-201 IHC results in various cancer tumor types and explore the relevance in determining eligibility to receive NEO-201 mAb therapy.
- Evaluate possible correlations between response rate and positivity of NEO-201 on tumor tissue samples. Immunohistochemistry (IHC) testing will be performed on pre-treatment tumor samples in order to perform exploratory correlations between level of positivity and clinical outcome.

- Explore the effects of NEO-201 on immunologic correlates associated with administration of NEO-201 monoclonal antibody therapy in subjects with relapsed or refractory solid tumors, including
    - Functional and phenotypic immune responses
    - Serum cytokine and chemokines and soluble factors.

3 STUDY DESIGN AND ENDPOINTS

3.1 DESCRIPTION OF THE STUDY DESIGN

This is a Phase I prospective, open label, dose escalation study using a standard 3 + 3 design to evaluate the safety (including treatment emergent adverse events (TEAE), dose limiting toxicities (DLT), maximal tolerated dose (MTD)) and recommended phase 2 dose (RP2D) of a therapeutic monoclonal antibody, NEO-201 in adults with advanced cancer (solid tumors). Subjects who meet eligibility criteria will be offered enrollment on this study.

NEO-201 will be administered on an outpatient basis (subjects may be admitted overnight for dose 1 and dose 2 for ease of pharmacokinetic sampling and may be hospitalized for grade 3 or 4 neutropenia during cycle 1 dosing of NEO-201 until ANC > 1000/mm^3 to reduce the risk of infection, neither of which will be considered an SAE) and will be infused intravenously every 2 weeks for 2 doses (28 days = 1 cycle). Subjects who receive at least 1 dose of NEO-201 will be evaluable for toxicity. During dose escalation, if a subject is removed from study therapy, for reasons other than toxicity, prior to completing Day 28 evaluation they will be considered inevaluable for DLT and will be replaced in the numbers.

Subjects will be evaluated for disease status after eight (8) weeks of dosing (4 doses of NEO-201), or after every 2 cycles. Subjects receiving at least one dose of NEO-201 will be evaluable for response. At the conclusion of the 2nd cycle, if a subject has not experienced a dose limiting toxicity (DLT), if restaging scans show stable disease (SD) or clinical response (PR) per RECIST criteria, and the subject chooses to proceed, they may be treated with additional cycles of NEO-201 until off treatment/off study criteria (Section **4.5.1**) are met.

Figure 11: Study Schema*

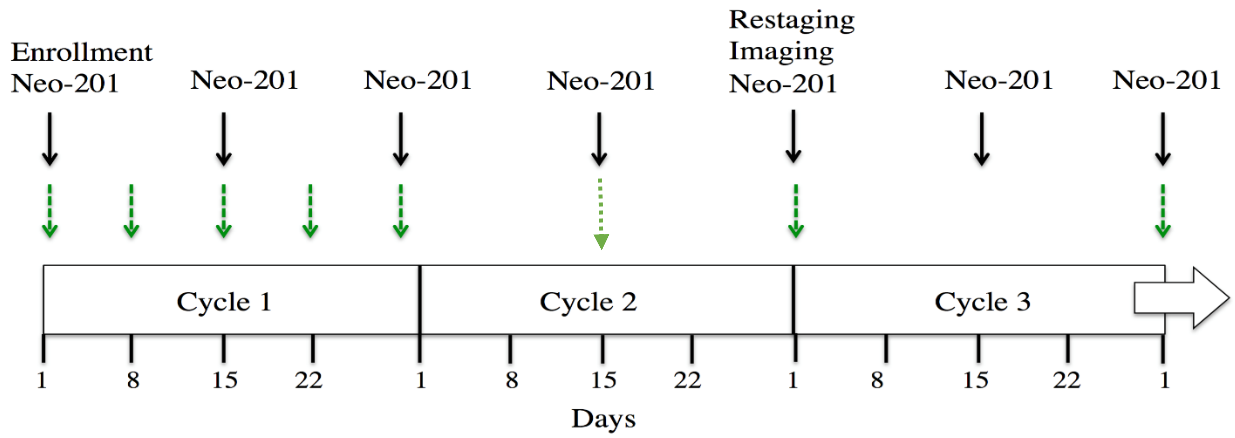

*** PK sampling: C1D1 (pre dose, immediately post dose, 1 and 4 hours post dose; C1D2 (24 hours post 1st dose); C1D4 (72 hours post 1st dose); C1D8 (7 days post 1st dose); C1D15 (pre dose, immediately post dose); C2D1 (pre dose, immediately post dose); C2D15 (pre dose, immediately post dose); C2D28 (evaluation).**

Once the MTD (or highest dose tested) is reached, the data will be analyzed to select expansion cohorts for the purpose of obtaining additional detailed data on toxicity, pharmacokinetics, NEO-201 expression by IHC, and conduct a preliminary evaluation of efficacy. Subjects enrolling in the expansion cohorts must be willing to undergo percutaneous tumor biopsies (twice), as long as these biopsies can be done safely with minimal discomfort.

Selection of expansion cohorts will include analysis of the phase 1 data and will be detailed in an amendment to this protocol. Analysis will include the results of IHC testing, PK analysis, safety evaluations, and any indication of clinical activity, as well as existing knowledge of the targeted tumor types. Analysis of IHC phase 1 data will be used in the determination of Phase 2 cohorts and cut-off eligibility for IHC, which will be conducted in a CLIA certified laboratory as part of screening determination of subjects enrolling in the expansion cohorts. Enrollment to the expansion cohorts will not proceed until the protocol amendment has been approved by IRB(s) and reviewed by the FDA.

3.1.1 Dose Escalation

Intrapatient dose escalation will not be permitted in this study due to the difficulty interpreting toxicity and pharmacokinetics findings in the presence of escalating doses of antibody. Doses will be escalated in 4 sequential dose levels as outlined in **Table 2.**

At least two (2) weeks followed by a toxicity assessment must elapse between the first infusion of study drug in the first subject of each dose cohort, and treatment of the second subject in that dose cohort. Forty-eight (48) hours must elapse between treatment initiation of subsequent subjects in a dose cohort. Subjects will be enrolled sequentially and enrollment will not proceed to the next higher dose level until all subjects have been treated in the prior cohort and the last subject has undergone a safety assessment on Day 29 (±4 days) of Cycle 1.

The safety assessment on Day 29 is essential for toxicity evaluation in determining the MTD. In the event a subject does not complete 2 doses of NEO-201 followed by a Day 29 safety assessment (for reasons other than toxicity), that subject will be considered inevaluable for MTD and will be replaced in the enrollment numbers.

Table 2: Dose Escalation Schedule

**Dose Escalation Schedule**		
**Dose Cohorts**	**Dose of IND Agent (mg/kg)**	**Number of Subjects planned for enrollment**
Level 1	1	3 - 6
*Level 1.5***	*1.5*	*3-6*
Level 2	2	3 - 6
*Level 2.5***	*3*	*3-6*
Level 3	4	3 - 6

*Level 3.5***	*5*	*3-6*
Level 4*	6	3 - 6
*additional doses may be investigated if no DLTs or clinical activity is observed.<br>** dose de-escalation cohorts		

Dose escalation will follow the rules as outlined in **Table 3** below.

Table 3: Rules for Dose Escalation and Definition of Maximum Tolerated Dose (MTD)

Number of Subjects with DLT at a Given Dose Level	Escalation Decision Rule
0 out of 3	Enter up to 3 subjects at the next dose level
≥ 2	Dose escalation will be stopped. This dose level will be declared the maximally administered dose (highest dose administered). Up to three (3) additional subjects will be entered at the next lowest dose level if only 3 subjects were treated previously at that dose.
1 out of 3	Enter up to 3 more subjects at this dose level.<br>• If 0 of these 3 subjects experience DLT, proceed to the next dose level.<br>• If 1 or more of this group suffer DLT, then dose escalation is stopped, and this dose is declared the maximally administered dose. UP to three (3) additional subjects will be entered at the next lowest dose level if only 3 subjects were treated previously at that dose.
≤1 out of 6 at highest dose level below the maximally administered dose	This is the MTD and is generally the recommended phase 2 dose. At least 6 subjects must be entered at the recommended phase 2 dose.

If 2 DLTs occur in subjects receiving NEO-201 at the 2 mg/kg dose and no DLTs or signs of activity were observed in subjects receiving NEO-201 at the 1 mg/kg dose, an additional 3-6 subjects may be enrolled to evaluate NEO-201 at 1.5 mg/kg.

If 2 DLTs occur in subjects receiving NEO-201 at the 4 mg/kg dose and no DLTs or signs of activity were observed in subjects receiving NEO-201 at the 2 mg/kg dose, an additional 3-6 subjects may be enrolled to evaluate NEO-201 at 3 mg/kg.

Similarly, if 2 DLTs occur in subjects receiving NEO-201 at the 6 mg/kg dose and no DLTs or signs of activity were observed in subjects receiving NEO-201 at the 4 mg/kg dose, an additional 3-6 subjects may be enrolled to evaluate NEO-201 at 5 mg/kg.

If no DLTS or signs of activity are observed in subjects receiving NEO-201 at 6 mg/kg, the investigators may propose an amendment to add additional dose levels to the Phase 1 design.

3.1.2 Dose Limiting Toxicity

Adverse events that are considered disease-related (not suspected of relationship to NEO-201) will not be considered dose-limiting toxicities. Only those AEs suspected to be related to the investigational agent NEO-201 will be used in the definition of DLT. Dose-limiting toxicity (DLT) will be defined as any one of the following:

- Grade 4 febrile neutropenia or grade 3 febrile neutropenia lasting longer than 72 hours.
- Grade 4 neutropenia that lasts > 7 days despite therapy with growth factor, or grade 4 thrombocytopenia that lasts >7 days, or grade 3 thrombocytopenia with bleeding.
- Any hematologic or non-hematologic toxicity greater than or equal to grade 3 (≥ grade 3), with the following exceptions:
    - Transient known toxicity related to infusion of monoclonal antibodies including fatigue, local reactions, flu-like symptoms (chills, muscle/body aches, nausea, vomiting or diarrhea), fever, and headache that recover to grade 1 or less within 8 hours after standard supportive treatment.
    - Grade 3 neutropenia that resolves to grade ≤ 2 with supportive growth factor therapy by the next scheduled dose (14 days) will **NOT** be considered a DLT.
    - Anemia that is considered a Grade 3 by CTCAEv5.0 (i.e. < 8.0 gm/dL) but is less than 2 gm/dL below baseline will **NOT** be considered a DLT.
    - Grade 3 Anemia that resolves to grade 1 or baseline by the next scheduled dose (14 days) will **NOT** be considered a DLT.
    - Toxicities with an alternative explanation to outside causes will **NOT** be considered a DLT.
    - The threshold for allowable grade 3 non-hematological events is <72 hours.
- Any infusion related reaction or allergic reaction/hypersensitivity to NEO-201 that meets the criteria for severe as defined in Section 16.2 Appendix B: Hypersensitivity/Infusion Reaction Algorithm.
- Any other Grade 4 toxicity suspected of relationship to NEO-201.
- Any death not clearly due to underlying disease or other extraneous causes
- ALT or AST > 8 X ULN (*ALT >328 U/L in men; >264 U/L in women; AST >320 U/L in men; >256 U/L in women*)
- ALT or AST > 5 X ULN (*ALT >205 U/L in men; >165 U/L in women; AST >320 U/L in men; >160 U/L in women*) for more than 14 days
- ALT or AST > 3 X ULN (*ALT >123 U/L in men; >99 U/L in women; AST >120 U/L in men; >96 U/L in women*) AND total bilirubin level > 2X ULN (> *2.4 mg/dL*) or INR > 1.5
- ALT or AST >3 X ULN (*ALT >123 U/L in men; >99 U/L in women; AST >120 U/L in men; >96 U/L in women*) with the appearance of fatigue, nausea, vomiting, right upper quadrant pain or tenderness, fever, rash, and/or eosinophilia (>5%)

CONFIDENTIAL:
NOT TO BE DISCLOSED WITHOUT PERMISSION FROM PRECISION BIOLOGICS - FOR QUALIFIED INVESTIGATORS AND THEIR IRBS ONLY

- All AEs of the specific grades should count as DLTs except those that are clearly and incontrovertibly due to disease progression or extraneous causes

3.1.3 Definition of Maximum Tolerated Dose (MTD)

The MTD will be the dose level at which no greater than 1/6 subjects has a DLT, and the next higher dose level has at least 2 subjects with a DLT.

3.1.4 Update to Dose Escalation with Amendment 4 and 5, version 27 October 2019 and 08 January 2020 respectively.

Three (3) subjects received NEO-201 at dose level 1 (1 mg/kg) for two doses without DLT. After completion of 2 doses in 3 subjects at dose level 2 (2 mg/kg), subject PB1801-006 had a DLT-prolonged neutropenia. The protocol was revised to recognize neutropenia as a known toxicity and allow for administration of filgrastim. Dose level 2 was subsequently expanded to 6 subjects (1 subject withdrew consent after the first dose and was therefore not evaluable for DLT and was replaced in the enrollment). PB1801-011 developed grade 4 febrile neutropenia (DLT) after requiring vasopressor support in the ICU. Given the high risk of infection based on predisposing factors in this patient population and the on target off tumor effects of NEO-201, all potential subjects will be screened for recent infections or infectious exposures. Any indications of a higher risk for infection or presence of indolent infectious sources will be thoroughly evaluated during screening. All subjects will be reviewed with an infectious disease consultant. Subjects initiating study therapy will have daily CBC testing daily during the first week (cycle 1) and may be hospitalized for grade 3 or 4 neutropenia during cycle 1 dosing of NEO-201 until ANC > $1000/mm^3$ to reduce the risk of infection (this hospitalization will not be considered an SAE as it is prophylactic in nature). An additional 3-6 subjects will be enrolled to an intermediate dose level 1.5 (1.5 mg/kg) to evaluate the ongoing safety of NEO-201 and the effectiveness of the risk mitigation interventions in this high-risk oncology population.

3.1.5 Expansion Cohorts

Upon determining MTD or highest dose evaluated if no MTD is found, additional subjects will be enrolled in select tumor groups based on analysis of Phase 1 data (to be added with a protocol amendment with input from biostatistician) and existing knowledge of targeted tumor types. The expansion cohorts will include those subjects evaluated at MTD in the dose escalation portion, and will conduct a preliminary evaluation of efficacy and collect additional data on NEO-201 safety and PK and immunohistochemistry of the target expression of NEO-201. Subjects enrolling in the expansion cohorts will be required to undergo immunohistochemistry (in a CLIA certified laboratory) testing of tumor samples (archived or biopsied) and meet the eligibility requirement established by analysis of Phase 1 data.

Selection of expansion cohorts will include analysis of the phase 1 data, including IHC, PK, safety, and indication of clinical activity, as well as knowledge of the targeted tumor types, and will be added with a protocol amendment. Selection of expansion cohorts may include subjects with colorectal cancer, pancreatic cancer, adenocarcinoma of the lung, squamous cell lung cancer, breast cancer, and mucinous and signet cell ovarian cancer. No additional subjects will be enrolled after Phase 1 until such protocol amendment is approved by the IRB and FDA.

CONFIDENTIAL:
NOT TO BE DISCLOSED WITHOUT PERMISSION FROM PRECISION BIOLOGICS - FOR QUALIFIED INVESTIGATORS AND THEIR IRBS ONLY

3.2 Study Endpoints

3.2.1 Primary Endpoint

- To determine the safety and RP2D, subjects will be assessed at each visit for incidence and severity of treatment-emergent adverse events (TEAE) recorded and graded according to the Common Terminology Criteria for Adverse Events (CTCAE) Version 5.0, as evidenced by:
    - Changes in clinical laboratory tests (clinical chemistry, hematology, etc).
    - Changes in vital signs (blood pressure, pulse, respiratory rate and body temperature).
    - Changes in physical exams. Signs and symptoms assessed may require additional testing as clinically indicated such as ECG, radiographic studies, etc.
    - Subject reported signs and symptoms.
- Dose Limiting Toxicities (DLTs) will be evaluated at each subject visit during the first cycle (from onset of first NEO-201 infusion through the Day 28 evaluation) according to the definition in section **3.1.2**. The safety assessment on Day 29 is essential for toxicity evaluation in determining the MTD. In the event a subject does not complete 2 doses of NEO-201 followed by a Day 29 safety assessment, unless the subject is removed from treatment for suspected adverse event, that subject will be considered inevaluable for toxicity and will be replaced in the enrollment numbers.
- Maximum tolerated dose (MTD) will be used in determining RP2D, and is defined in section **3.1.3.** To most accurately determine RP2D, if -DLTs occur at 2 mg/kg, and no DLTs or signs of activity were observed in subjects at 1 mg/kg, an additional 3-6 subjects may be enrolled to evaluate NEO-201 at 1.5 mg/kg; additionally, if DLTs occur at 4 mg/kg and no DLTs or signs of activity were observed in subjects at 2 mg/kg, an additional 3-6 subjects may be enrolled to evaluate NEO-201 at 3 mg/kg. Similarly, if DLTs occur at 6 mg/kg and no DLTs or signs of activity were observed in subjects at 4 mg/kg, an additional 3-6 subjects may be enrolled to evaluate NEO-201 at 5 mg/kg. If no DLTS or signs of activity are observed in subjects receiving NEO-201 at 6 mg/kg, the investigators may propose an amendment to add additional dose levels to the Phase 1 design. Statistical analyses will be descriptive in nature and consist of the number and percentage of subjects in each category for discrete variables, and the sample size, mean, median, S.D., minimum and maximum for continuous variables.

3.2.2 Accrual Target and Study Duration

To complete the primary objective, 3 to 6 subjects will be enrolled in each of the 5 (1mg/kg, 1.5mg/kg, 2mg/kg, 4mg/kg, 6 mg/kg) dose cohorts to a maximum of 30 subjects to determine MTD. If one of the additional dose cohorts is added (either 3 mg/kg or 5 mg/kg), an additional 6 subjects may be enrolled. With Amendment 4 (version 27 OCT 2019) an additional 3 subjects were added to dose level 2 (2 mg/kg) to expand the safety evaluation after instituting additional safety parameters. Therefore, a maximum (6 + 9 + 6+ 6 + 6) of 36 subjects may be enrolled in the Phase I study. The study will allow for up to 5 subjects to be replaced in a dose cohort in the event the subject become inevaluable for MTD and require replacement in the protocol numbers. The maximum accrual for the phase 1 portion of this protocol will be 36 +5 for a total of 41 subjects.

CONFIDENTIAL:
NOT TO BE DISCLOSED WITHOUT PERMISSION FROM PRECISION BIOLOGICS - FOR QUALIFIED INVESTIGATORS AND THEIR IRBS ONLY

It is expected that with accrual of 3-4 subjects per month, the phase 1 portion of this study will be completed in 18 months.

3.2.3 Secondary Endpoints

The secondary endpoint for this study is the character and incidence of grade 1 through grade 4 toxicities following biweekly infusions of NEO-201 according to the Common Terminology Criteria for Adverse Events (CTCAE) Version 5.0., to include severity, relationship to study agent, and expectedness.

- Safety data will be analyzed per standard methods and interpreted descriptively for each dose cohort. Safety data will be summarized for each dose cohort separately and for all dose cohorts combined. Adverse events will be assessed using the CTCAE version 5.0 for type and severity of event. Serious Adverse Events will be summarized for each dose cohort and for all dose cohorts.
- Safety analysis will include AE incidence summary tables and incidence of treatment emergent adverse events (TEAEs) (i.e. AEs that began on or after the first dose of any study drug through 30 days after the last dose of study drug. If a subject experiences multiple episodes of the same event, the subject will be counted once for that particular event at the highest grade.

An additional secondary endpoint is to characterize the pharmacokinetics (PK) of NEO-201 monotherapy, including AUC, Cmax, Cmin using intensive sampling. Pharmacokinetics (PK) will be characterized from blood samples collected on all subjects according to the schedule in section **6.1.2.1.** Bioanalysis of serum samples will be performed using a precise, validated ELISA method. NEO-201 pharmacokinetics will be evaluated using a non-compartmental approach. Individual concentration-time profiles will be constructed for each subject for the first cycle (28 days). The reported maximum plasma concentration (C_{max}) and the time of maximum plasma concentration (T_{max}) will be the observed values. Peak and trough concentrations for each dose will be reported as the concentration of NEO-201 within three minutes after the end of infusion and the NEO-201 concentration immediately prior to the next treatment (approximately 14 days later). Drug exposure will be estimated using the area under the concentration-time curve (AUC). The AUC from time zero to the time of the final quantifiable sample (AUC_{last}) will be calculated using the linear trapezoidal method. The AUC from time zero to infinity (AUC_{inf}) will be calculated by extrapolation, using the terminal rate constant (λ_Z) from the last measurable concentration. Half-life ($t_{1/2}$) will be defined by the terminal rate constant, using a standard equation. The AUC during the dosing interval (AUC_{tau}) will be calculated by extrapolation using the terminal rate constant from the last measurable concentration to estimate concentration at 336 h (the 14 day dosing interval), followed by calculation utilizing the log-linear trapezoidal method. Estimated accumulation will be calculated as C1 AUC_{inf}/C1 AUC_{tau} observed accumulation ratio will be calculated as C4 C_{max}/C1 C_{max}.

3.2.4 Exploratory Endpoints

- Assess immunogenicity of NEO-201 in adults with relapsed or chemo-resistant solid tumors.

While monoclonal antibodies represent potential effective therapies, the development of immunogenicity can impact both the safety and pharmacokinetic properties of the mAb, affecting

both the utility and efficacy of the NEO-201. Human anti-human antibody testing will be conducted by Precision Biologics, Inc. to evaluate the immunogenicity of NEO-201. Results will be reported descriptively.

- Determine in a preliminary fashion the Objective Response Rate (ORR = CR, PR, SD) as determined by RECIST v1.1 guidelines, and progression free survival, (PFS). PFS is defined as the duration of time from the first dose of NEO-201 to time of progression or death, whichever occurs first, to be assessed in both the dose-escalation cohort and dose expansion cohorts.

RECIST v1.1 will be used to assess response by radiologists. Evaluable subjects will be assigned one of the following categories: 1) Complete Response; 2) Partial Response; 3) Stable Disease; 4) Progressive Disease; and 5) not evaluable (defined as death from malignant disease, death from toxicity, death due to other causes, or unknown-not assessable, insufficient data, unable to complete at least two doses of NEO-201). The percentage of subjects that achieve the complete or partial overall response at C2D28 will be summarized. The following will be presented: sample size, overall response rate, and 95% CI of the response rate using exact binomial method. Best overall response at C2D28 will be presented in a data listing.

- Describe NEO-201 IHC results in various cancer tumor types and explore the relevance in determining eligibility to receive NEO-201 mAb therapy.

IHC evaluations of tumor samples will be conducted in all subjects enrolling on this trial. Results will include the percentage of tumor cells staining with NEO-201 and staining intensity, as formal validation of the IHC assay is completed. When available analysis of the IHC results will be correlated with the characteristics of enrolled subjects, including tumor type, response to NEO-201, and occurrence of adverse events, to aid in the design of the expansion cohorts, which will be added to this study with an amendment.

- Evaluate possible correlations between response rate and positivity of NEO-201 on tumor tissue samples. Immunohistochemistry (IHC) testing will be performed on pre-treatment tumor samples in order to perform exploratory correlations between level of positivity and clinical outcome.

- Explore the effects of NEO-201 on immunologic correlates associated with administration of NEO-201 monoclonal antibody therapy in subjects with relapsed or refractory solid tumors, including
    - Functional and phenotypic immune responses
    - Serum cytokine and chemokines and soluble factors.

Results of immunologic correlates will be reported descriptively, with means, medians, ranges, where appropriate, and when available will be correlated with clinical factors including response. The analysis of these results will be hypothesis generating and may be expanded in the expansion cohorts for further analysis.

4 STUDY ENROLLMENT AND WITHDRAWAL

4.1 PARTICIPANT INCLUSION CRITERIA

Eligible subjects must meet the following inclusion criteria:

- ✓ AGE: ≥ 18 years of age.
- ✓ DIAGNOSIS:
  - o Subjects must have histologically or cytologically confirmed recurrent, locally advanced unresectable or metastatic cancer confirmed by the Laboratory of Pathology, NCI.
    - o Subjects who are not eligible for standard therapy that is known to confer clinical benefit for respective tumor type
  - o Must have archived tissue (10 unstained slides or tissue block), or must have tumor which can be safely biopsied percutaneously and be willing to undergo a tumor biopsy.
  - o Dose Escalation: Subjects must have colorectal cancer, pancreatic cancer, adenocarcinoma of the lung, squamous cell lung cancer, breast cancer, and mucinous and signet cell ovarian cancer (cancer types in which tumor samples (> 50%) historically stain positive for NEO-201 expression).
- ✓ MEASURABLE/EVALUABLE DISEASE: Subjects must have disease that is measurable by RECIST 1.1 or evaluable by bone scan, peritoneal or pleural effusions, or carcinomatosis.

- PERFORMANCE STATUS: ECOG ≤ 2; or Karnofsky performance status of ≥ 50% (See section **16.1** Appendix A)

- LABORATORY FUNCTION:

  Screening laboratory data within 21 days of the first dose of study drug. Subject must have adequate organ function:

  - ✓ Hemoglobin > 9 g/dL, or on stable doses (hematocrit stable within 1 gram and dose stable for one month) of erythropoietin or similar medication.
  - ✓ Absolute neutrophil count (ANC) ≥1,500/mm3.
  - ✓ Platelets ≥ 100,000/mm3.
  - ✓ Total bilirubin ≤ 2.0 mg/dL
  - ✓ ALT and AST ≤ 3 times the ULN, or, if the subject has liver metastases, ≤ 5 times the ULN.
  - ✓ Creatinine ≤ 1.5 mg/dL or creatinine clearance > 40 mL/min/1.73 m^2 for subjects with creatinine levels above institutional normal, as calculated by the Cockcroft Gault formula.

- INFORMED CONSENT: Voluntary written informed consent before performance of any study-related procedure that is not part of normal medical care.

CONFIDENTIAL:
NOT TO BE DISCLOSED WITHOUT PERMISSION FROM PRECISION BIOLOGICS - FOR QUALIFIED INVESTIGATORS AND THEIR IRBS ONLY

- PRIOR THERAPY:
  - ✓ At least 14 days must have elapsed since treatment with oral tyrosine kinase inhibitors, or until toxicities associated with TKI therapy have resolved.
  - ✓ At least 21 days must have elapsed since treatment with previous monoclonal antibodies, or until toxicities associated with mAb therapy have resolved.
  - ✓ At least 4 weeks must have elapsed since any chemotherapeutic agents at the time of enrollment (or 6 weeks for regimens containing BCNU or mitomycin C).
  - ✓ At least 2 weeks must have elapsed since any systemic corticosteroids at the time of enrollment
  - ✓ Immunotherapy: At least 42 days after the completion of any type of immunotherapy, e.g. tumor vaccines.
  - ✓ XRT: At least 7 days after local palliative XRT (small port)
- Subjects must have recovered from any acute toxicity related to prior therapy, except for alopecia. Toxicity should be ≤ grade 1, or ≤ grade 2 for peripheral neuropathy or hypothyroidism.
- Subject is expected to be able to remain on a study protocol for at least 8 weeks.
- BIRTH CONTROL: Female subject is post-menopausal, surgically sterilized, or willing to use acceptable methods of birth control (i.e., a hormonal contraceptive, intra-uterine device, diaphragm with spermicide, or condom with spermicide, or abstinence) for the duration of the study.

  Should a woman become pregnant or suspect she is pregnant while she or her partner is participating in this study, she should inform her treating physician immediately. Men treated or enrolled on this protocol must also agree to use adequate contraception prior to the study, for the duration of study participation, and 2 weeks after completion of NEO-201 administration.

4.2 PARTICIPANT EXCLUSION CRITERIA

Subjects meeting any of the following exclusion criteria are <u>not</u> to be enrolled in the study:

- Subject has history of disseminated or uncontrolled brain metastases or central nervous system disease. Brain metastases will be considered controlled if SD on two consecutive brain MRIs, performed at least 2 months apart, and subject is without seizures.
- History of allergic reactions attributed to compounds of similar chemical or biologic composition to NEO-201 or other agents used in this study.
- Any major surgery within 14 days of enrollment.
- Subjects who are receiving any other investigational agents.
- Subject does not have archival tissue available and does not have a lesion(s) that can be safely biopsied via percutaneous route, or is unwilling to undergo biopsy.

CONFIDENTIAL:
NOT TO BE DISCLOSED WITHOUT PERMISSION FROM PRECISION BIOLOGICS - FOR QUALIFIED INVESTIGATORS AND THEIR IRBS ONLY

- Subject has an uncontrolled concomitant illness including, but not limited to, ongoing or active infection, uncontrolled diabetes mellitus, symptomatic congestive heart failure, unstable angina pectoris, hypokalemia, family history of Long QT Syndrome or presence of cardiac arrhythmia.
- Subjects who are assessed to have unacceptable risk of developing infection from neutropenia will be excluded at the Investigator's discretion.
- HIV-positive subjects on combination antiretroviral therapy are ineligible because of the unknown potential for pharmacokinetic interactions with NEO-201. In addition, these subjects are at increased risk of lethal infections which could complicate the toxicity assessment of this study. Appropriate studies will be undertaken in subjects receiving combination antiretroviral therapy when indicated.
- Subject has other serious medical illness, including a second malignancy, or psychiatric illness that could, in the Investigator's opinion, potentially interfere with the completion of treatment according to this protocol.
- Pregnant women are excluded from this study because the potential for teratogenic or abortifacient effects due to NEO-201 is unknown. Because there is an unknown but potential risk for adverse events in nursing infants secondary to treatment of the mother with NEO-201, breastfeeding should be discontinued if the mother is treated with NEO-201.

4.3 Strategies for Recruitment and Retention

The following recruitment strategies will be employed to elicit potential candidates for this trial:

1. During the Phase 1 portion of the study, subjects being seen at NIH clinics with the targeted disease types;
2. Subjects treated on other institutional trials who are eligible for participation;
3. Listed on clinical trials.gov;
4. Listed in PDQ.

Subject accrual to the expansion cohorts in this protocol will be facilitated by adding participating institutions, developed to increase the accrual to clinical studies via community outreach, as well as recruitment letters to referring physicians. Prior to distribution of any recruitment materials, such materials will be submitted to the IRB for review.

4.4 Subject Screening, Enrollment and Registration Procedures

Prospective subjects will be screened on this study (or on a screening protocol as per institutional SOP).

Before conducting any screening tests or evaluations that are not part of standard medical care, the subject must give written informed consent. All subjects must sign and date the IRB/IEC approved consent form before initiating any study specific procedures or activities that are not part of a subject's routine care.

CONFIDENTIAL:
NOT TO BE DISCLOSED WITHOUT PERMISSION FROM PRECISION BIOLOGICS - FOR QUALIFIED INVESTIGATORS AND THEIR IRBS ONLY

Each subject who completes the screening period and is enrolled on the study will receive from the sponsor a unique subject identification number before any study specific procedures or activities are initiated. This number will be used to identify the subject throughout the study and must be used on all study documentation related to the subject. Furthermore, the subject identification number must remain constant throughout the entire clinical study.

Subjects who are evaluated and determined to be eligible will have an Eligibility Checklist completed according to source documentation, and e-mailed to the Medical Advisor (Phil Arlen, MD; cell phone: 301-728-4883) of the Sponsor; E-mail: Philip.Arlen@Precision-Biologics.com, prior to initiation of treatment with study drug. Subjects will be identified only by the subject initials. Once the Medical Advisor reviews and approves eligibility (via e-mail) and assigns the subject identification number and dose level, the subject may proceed to treatment. During dose escalation, subjects will be approved on a first come, first serve basis. Contact the Sponsor if there is a question about enrollment slot availability. The assigned subject identification number will be used to identify the subject for all procedures and study-related documentation.

Subjects that do not meet screening criteria should be removed from the study following the procedure in section **4.5.2**.

4.5 PARTICIPANT WITHDRAWAL OR TERMINATION

4.5.1 Reasons for Withdrawal or Termination

4.5.1.1 Off Protocol Therapy

Prior to documenting removal from protocol therapy, effort must be made to have all subjects complete a safety visit approximately 30 days following the last dose of study therapy.

Subjects will be taken off protocol therapy for any of the following events:

- Progressive disease
- Participant requests to be withdrawn from active therapy
- Dose Limiting Toxicity as defined in Section 3.1.2.
- Investigator discretion
- Positive pregnancy test

4.5.1.2 Off-Study Criteria

Subjects will be taken off study for any of the following events:

- Completed study follow-up period
- Participant requests to be withdrawn from study
- Death
- Screen failure

CONFIDENTIAL:
NOT TO BE DISCLOSED WITHOUT PERMISSION FROM PRECISION BIOLOGICS - FOR QUALIFIED INVESTIGATORS AND THEIR IRBS ONLY

4.5.2 Handling of Participant Withdrawals or Termination

Authorized staff must notify the Sponsor when a subject is taken off protocol therapy and when a subject is taken off-study. A Participant Status Updates Form from sponsor must be completed and sent via encrypted email the sponsor.

If a subject enrolled in the Phase 1 portion of the study is taken off treatment prior to Day 28 evaluation for any reason other than treatment-emergent toxicity, that subject will be replaced in the accrual numbers. Every effort will be made to complete a Day 30 evaluation after the last dose of NEO-201. If the subject cannot return to the participating site for this visit, a request will be made to collect required clinical labs (specify as needed) from a local physician or laboratory. If this is not possible, subjects may be assessed by telephone for symptoms.

4.5.2.1 Subject Withdrawals (off therapy and off study Procedures)

Authorized staff must notify the Sponsor when a subject is taken off protocol therapy and when a subject is taken off-study. A Participant Status Updates Form from sponsor must be completed and sent via encrypted email the sponsor.

4.6 PREMATURE TERMINATION OR SUSPENSION OF STUDY

This study may be prematurely terminated, if in the opinion of Precision Biologics Inc., there is sufficient reasonable cause. The Investigator may terminate his/her participation for reasonable cause. The terminating party must provide written notification documenting the reason for study termination.

Circumstances that may warrant termination include, but are not limited to:

- Determination of unexpected, significant, or unacceptable risk to subjects;
- Failure to enter subjects at an acceptable rate;
- Insufficient adherence to protocol requirements;
- Insufficient and/or inadequate evaluable data; or
- Plans to modify, suspend or discontinue the development of the study drug.

5 STUDY AGENT

5.1 STUDY AGENT AND CONTROL DESCRIPTION

5.1.1 Acquisition

NEO-201 (h16C3) is a monoclonal antibody that was developed against a semi-purified human membrane protein preparation derived from colon cancer tissues. cGMP NEO-201 was manufactured by Catalent Pharma Solutions for Precision Biologics Inc. and will be supplied by Precision Biologics Inc. for use in this study. Participating site PIs (or their pharmacy designee) may request NEO-201 supply by contacting Precision Biologics Inc. according to the PB SOP Drug Requests.

5.1.2 Formulation, Appearance, Packaging and Labeling

NEO-201 was formulated in Tween 80 (Polysorbate 80) to achieve a final concentration of 0.1% Tween 80 in 25 mM Citrate, 150 mM NaCl, pH 6.5. Each vial should be inspected prior to use, and appears as a clear, colorless to slightly yellow solution, essentially free from particles. The concentration of the NEO-201 final vialed product is 10.2 mg/mL, in 10 mL per vial, thus 100 mg/vial (single use vials). Each vial is labeled with the drug name and concentration, sponsor name, and storage conditions. The vials are also labeled "for investigational use only". DO NOT ADMINISTER IF THERE IS VISUAL PRECIPITATE IN THE BAG OR VIAL. Any unused agent should be discarded as medical waste and should not be reused or re-frozen for later use.

5.1.3 Product Storage and Stability

Vials of NEO-201 should be stored frozen at -20 ^{o}C ± 10 ^{o}C (short excursions outside this range [but still < 0 ^{o}C] can be tolerated but must be reported to sponsor) until ready for subject administration. Stability testing studies are ongoing.

5.1.4 Preparation

NEO-201 must be administered through an in-line, nonpyrogenic, low-protein binding filter (0.2 or 0.22 microns) and controlled by a continuous infusion device. Administration through a separate IV line, without mixing with other medications is required as compatibility testing has not yet been performed.

Prior to administration, vial(s) should be thawed at room temperature. NEO-201 should be reconstituted within 24 hours of thawing (stored in refrigerator or at room temperature). Reconstituted NEO-201 in 0.9% sodium chloride USP may be stored to up to 48 hours refrigerated (2 to 8 °C).

NEO-201 infusion should be completed within 6 hours of infusion initiation. DO NOT ADMINISTER IF THERE IS VISUAL PRECIPITATE IN THE BAG OR VIAL. Any unused agent should be discarded as medical waste and should not be reused or re-frozen for later use.

5.1.5 Dosing and Administration

NEO-201 will be administered on an outpatient basis (patients may be admitted overnight for dose 1 and dose 2 for ease of pharmacokinetic sampling and may be hospitalized for grade 3 neutropenia during cycle 1 dosing of NEO-201 until ANC > 1000/mm3 to reduce the risk of infection, which will not be considered an AE/SAE). NEO-201 will be infused intravenously every 2 weeks for 2 doses (28 days = 1 cycle). NEO-201 will be administered by intravenous continuous infusion in 250 mL of 0.9% sodium chloride USP using tubing with 0.22 micron non (or low) protein binding filter, with a volumetric pump. Administration through a separate IV line, without mixing with other medications is required as compatibility testing has not yet been performed.

Weight should be obtained on Day 1 of each cycle. This weight will be used for the dose calculation of NEO-201 for that cycle.

NEO-201 should be administered intravenously initially at 0.5mg/min. NEO-201 may be interrupted or infused at a lower rate if clinically indicated, as per discretion of the investigator.

CONFIDENTIAL:
NOT TO BE DISCLOSED WITHOUT PERMISSION FROM PRECISION BIOLOGICS - FOR QUALIFIED INVESTIGATORS AND THEIR IRBS ONLY

Infusion rates will be increased as tolerated in 0.5 mg/min increments every 30 minutes to a maximum rate of 4 mg/min.

Subsequent doses may be administered at the highest tolerated rate of infusion, and/or adjusted based on subject tolerance.

5.1.5.1 Vital Sign Monitoring during Drug Administration

- Pre infusion of study drug.
- Every 15 minutes (± 5 minutes) from start of infusion, times four (4). If vital signs are stable and no evidence of an adverse reaction is noted, vital signs can be performed hourly until one hour after completion of study drug infusion.
- If infusion of study drug is interrupted at any time, vital signs should continue to be taken every 15 minutes (± 5 minutes) during the interruption period and until one hour after the time infusion of study drug is re-started. Vital signs will continue to be assessed every 15 minutes until stable, at which time vital signs will be taken hourly until 1 hour after the completion of infusion.

5.1.5.2 Premedication

To alleviate signs/symptoms of infusion reaction/allergic reaction, subjects may be treated with acetaminophen (650-1000mg PO), dexamethasone (10 mg I.V.), diphenhydramine (25-50 mg I.V. or po) and ranitidine (50 mg I.V.) or equivalent premedication regimen (See Section **16.2** Appendix B).

For management of infusion reactions related to NEO-201 refer to section **5.1.9** Dose Modifications.

5.1.6 Route of Administration

NEO-201 will be administered intravenously.

5.1.7 Incompatibilities

No incompatibility studies are available at this time.

5.1.8 Starting Dose and Dose Escalation Schedule

The starting dose of 1 mg/kg will be administered based on preclinical pharm-tox studies. Doses will be escalated in groups of 3-6 subjects as described in Section **3.1.1.** There will be no intrasubject dose escalation to prevent confounding

5.1.9 Dose Adjustments/Modifications/Delays

There will be no dose modifications of NEO-201 during the phase 1 portion of this trial. Infusion reactions may necessitate slowing or interrupting the intravenous infusion of NEO-201, and supportive medications can be administered (See Section **16.2** Appendix B), but doses will not be skipped or dose reduced.

CONFIDENTIAL:
NOT TO BE DISCLOSED WITHOUT PERMISSION FROM PRECISION BIOLOGICS - FOR QUALIFIED INVESTIGATORS AND THEIR IRBS ONLY

Growth factor support (i.e. filgrastim) will be allowed to facilitate recovery of neutrophil counts.

Doses may be delayed at investigator discretion for events not suspected to be related to NEO-201 study agent.

5.1.10 Duration of Therapy

In the event that a subject is receiving clinical benefit (SD or lessening of symptoms in the absence of PD) without significant toxicity (no SAE or DLT) after the initial 2 cycles, additional cycles may be offered after consultation with the sponsor until off treatment/off study criteria (section **4.5.1.1** and section **4.5.1.2**) are met.

5.1.11 Tracking of Dose

Study drug should only be dispensed to eligible subjects who have been enrolled by the sponsor. Each drug administration should be documented with start time, stop time, total dose administered.

5.1.12 Study Agent Accountability Procedures

Accountability for the study drug at the study center is the responsibility of the Investigator. The Investigator will ensure that the study drug is used only in accordance with this protocol. Where allowed by site SOPs, the Investigator may choose to assign some of the drug accountability responsibilities to a pharmacist or other appropriate individual. Drug accountability records indicating the drug's delivery date to the center, inventory at the center, use by each subject, and return to Precision Biologics Inc. (or disposal of the drug, if approved by Precision) will be maintained by the clinical site. These records will adequately document that the subjects were provided the doses as specified in the protocol and should reconcile all study drug received from Precision Biologics, Inc. Accountability records will include dates, quantities, lot number, expiration dates (if applicable), and subject numbers. The sponsor or its designee will review drug accountability at the study site on an ongoing basis during monitoring visits.

All unused study drug will be retained at the study site until inventoried by the monitor. All unused or expired study drug will be returned to Precision Biologics, Inc. or if authorized, disposed of at the study site as hazardous waste in accordance with governing regulations.

6 STUDY PROCEDURES AND SCHEDULE

6.1 STUDY PROCEDURES/EVALUATIONS

6.1.1 Study Specific Procedures

6.1.1.1 IHC Evaluation

After obtaining a signed consent, 10 or more unstained slides or tissue block will be sent to Dr. Annunziata's research team. Christina M. Annunziata, M.D., Ph.D., Women's Malignancies Branch (WMB), CCR, NCI:

10 Center Drive, Room 3B43A
Bethesda, MD 20892

If archived tissue block is not available, subject must be eligible and willing to undergo percutaneous core biopsy of an easily accessible lesion. Subjects may be enrolled prior to undergoing biopsy, but the biopsy must be performed prior to the first dose of study drug. Tissue samples from subjects in the dose escalation cohorts will be batched for subsequent IHC testing (CLIA laboratory NCI Frederick MD) and evaluation (Stephen Hewitt, M.D., Laboratory of Pathology, CCR, NCI).

6.1.2 Correlative Assays, including Preparation, Handling and Storage

The following samples will be collected during participation in this clinical trial:

**Test/assay**	**Volume blood (approx)**	**Type of tube**	**Collection point (+/- 48hrs)**	**Location of specimen storage/analysis**
Immune- monitoring	20 mL	Green top	Baseline, 24 hrs, 72 hrs post dose 1, pre dose 2, Day 56 (end of cycle 2)	Dr. Christina Annunziata's Laboratory
	10 mL	Red top		
	20 mL	Green top	Baseline, pre dose 2, Day 56 (end of cycle 2)	T-regs:
PK	5 mL	Red top	See Section 6.1.2.1	Precision Biologics, Inc Laboratories
Immunohistochemistry (IHC)	Archival tumor tissue	10 unstained slides or tissue block	Baseline	Dr. Stephen Hewitt, Laboratory of Pathology, NCI
HAHA	5 mL	Red top	Baseline, 72 hrs post dose 1, pre dose 2, Day 56 (end of cycle 2)	Precision Biologics, Inc Laboratories

6.1.2.1 Pharmacokinetics (PK)

Serial PK analysis will be performed in all subjects in the dose escalation portion of this study at the time points listed below. Five (5) ml of blood will be collected in a red-top tube at each time point and processed according to directions in the Laboratory Manual.

<u>*Cycle 1:*</u>

- Day 1 (Dose 1):
    - Pre dose
    - Immediately after end of study drug infusion (no longer than 3 minutes post)
    - 1 (±5 min) hour and 4 (±10 min) hours post infusion (Cycle 1 only)

- Day 2: 24 hours (±1 hour) post infusion(Cycle 1 only)
- Day 4: 72 (±3 hours) hours post infusion (Cycle 1 only)
- Day 8: 7 days (±3 hours) post infusion (Cycle 1 only)
- Day 15 (Dose 2): pre dose and immediately after end of study drug infusion (no longer than 3 minutes post)

<u>*Cycle 2:*</u>

- Day 1 (Dose 3): pre dose and immediately following after end of study drug infusion (no longer than 3 minutes post)
- Day 14 (Dose 4): pre dose and immediately after end of study drug infusion (no longer than 3 minutes post)
- Day 28 (End of 2nd Cycle Evaluation) (±4 days)

6.1.2.2 Human Anti-Human Antibody (HAHA) for Immunogenicity

While monoclonal antibodies represent potential effective therapies, the development of immunogenicity can impact both the safety and pharmacokinetic properties of the mAb, affecting both the utility and efficacy of the NEO-201. Human anti-human antibody testing will be conducted by Precision Biologics, Inc. to evaluate the immunogenicity of NEO-201. Results will be reported descriptively.

During Cycle 1, blood samples will be drawn for HAHA to measure anti-drug antibody development in subjects receiving NEO-201. HAHA will be measured by ELISA using a qualified method for use in this application that is developed in Precision Biologics, Inc.

Blood samples will be drawn, processed and shipped to Precision Biologics, Inc. according to the details specified in the Laboratory Manual at the following time points:

- Day 1: Pre dose 1
- Day 4: 72 hours (±3 hours) after end of infusion of dose 1
- Day 15: Pre dose 2
- Day 56 (End of 2nd Cycle Evaluation) (±4 days)

6.1.2.3 Immunologic Correlates

Samples for immunologic correlates associated with administration of NEO-201 monoclonal antibody therapy including functional and phenotypic immune processes and serum cytokine and chemokines and soluble factors will be collected in all subjects at baseline, 24 and 72 hours after the first dose of NEO-201, prior to dose administration on D15, and at the cycle 2 evaluation (Day 56). Samples will be collected and processed according to the NEO-201 Laboratory Manual. In brief, red top tubes will be processed to collect serum from clotted blood and cryopreserved. Green top tubes will be processed to isolate and cryopreserve PBMCs for immune-monitoring studies. An additional green top tube will be collected for Treg analysis associated with the administration of NEO-201 at Baseline, pre-dose 2 and at the conclusion of cycle 2 (Day 56). Processed samples will be stored in Dr. Annunziata's Laboratory pending analysis.

CONFIDENTIAL:
NOT TO BE DISCLOSED WITHOUT PERMISSION FROM PRECISION BIOLOGICS - FOR QUALIFIED INVESTIGATORS AND THEIR IRBS ONLY

6.1.2.4 IHC Evaluation

After obtaining a signed consent, 10 or more unstained slides or tissue block will be sent to Dr. Annunziata's research team. Christina M. Annunziata, M.D., Ph.D., Women's Malignancies Branch (WMB), CCR, NCI:

10 Center Drive, Room 3B43A
Bethesda, MD 20892

If archived tissue block is not available, subject must be eligible and willing to undergo percutaneous core biopsy of an easily accessible lesion. Subjects may be enrolled prior to undergoing biopsy, but the biopsy must be performed prior to the first dose of study drug. Tissue samples from subjects in the dose escalation cohorts will be batched for subsequent IHC testing (CLIA laboratory NCI Frederick MD) and evaluation (Stephen Hewitt, M.D., Laboratory of Pathology, CCR, NCI).

6.1.2.5 Sample Prioritization

In the event the quantity of blood sample collection at any timepoint, exceeds the institutional limits of blood collected for research samples, blood collection for samples will be completed according to the following prioritization:

1. PK

2. HAHA

3 Immune monitoring

4. T regs

6.1.2.6 Sample Storage, Tracking and Disposition

All specimens obtained in the protocol are used as defined in the protocol. Any specimens that are remaining at the completion of the protocol will be stored in the conditions described within this protocol. The study will remain open so long as sample or data analysis continues. Samples from consenting subjects will be stored until they are no longer of scientific value or if a subject withdraws consent for their continued use, at which time they will be destroyed. The PI will report any loss or destruction of samples to the IRB and the Sponsor as soon as he is made aware of such loss.

If the subject withdraws consent the participant's data/specimens will be excluded from future distributions, but data/specimens that have already been distributed for approved research use will not be able to be retrieved.

The PI will report destroyed samples to the IRB and Sponsor if samples become unsalvageable because of environmental factors (ex. broken freezer or lack of dry ice in a shipping container) or if a subject withdraws consent. Samples will also be reported as lost if they are lost in transit between facilities or misplaced by a researcher. Freezer problems, lost samples or other problems associated with samples will also be reported to the institution's IRB according to local policy and procedures.

CONFIDENTIAL:
NOT TO BE DISCLOSED WITHOUT PERMISSION FROM PRECISION BIOLOGICS - FOR QUALIFIED INVESTIGATORS AND THEIR IRBS ONLY

Instructions for sample collection, labeling, storage and shipment are contained in the Precision Biologics Laboratory Manual.

6.2 STUDY SCHEDULE

6.2.1 Screening

- Demographics, including date of birth, gender, race and ethnicity.
- Complete medical history, including history of other health problems as well as history of cancer and associated therapies.
- Complete history of prior infections (within past 6 months) or history of recurrent infections, including source of infection, treatment of infection, frequency and symptoms.
  - If history of recent (6 months) or recurrent infections, must complete a thorough work up to rule out evidence of subclinical infection, which may include any of the following based on recommendations of Infectious Disease (ID) Consult:
    - ✓ Careful travel history
    - ✓ Exposure history to persons with fever, known infectious diseases, animals, ticks, fresh or sea water; recent dental procedures.
    - ✓ Additional scans or cultures if indicated
  - Subjects must be cleared by ID Consult prior to enrollment in order to be eligible, with a plan for close infection monitoring especially during cycle 1.
- Complete physical examination.
- Body weight and height. (Weight to be used for cycle 1 dose calculation of investigational agent unless body weight on Day 1 increases or decreases greater than 10%, in which case Day 1 body weight will be used.)
- Vital signs: systolic and diastolic blood pressures, respiratory and heart rates, and temperature (°C).
- ECOG or Karnofsky performance status (see section **16.1** Appendix A).
- Laboratory tests:
  - CBC with differential and platelet counts,
  - PT/INR and APTT, fibrinogen.
  - Chemistries: BUN, serum creatinine, uric acid, LDH, calcium, magnesium, sodium, chloride, potassium, carbon dioxide, alkaline phosphatase, total bilirubin, direct bilirubin, AST (SGOT), ALT (SGPT), protein, glucose and albumin, C-reactive protein.
  - Appropriate tumor marker for the cancer type, i.e. CA19-9, CEA, BRCA, etc.
  - Thyroid function: T3, T4, TSH.
- Urinalysis
- Urine/serum for pregnancy test (women of childbearing potential)

- Radiologic evaluation of disease site(s): for example: CT scan with contrast, unless contraindicated, PET-CT, MRI, etc. within 28 days prior to Cycle 1 Day 1.
- Review medical history to determine eligibility based on inclusion/exclusion criteria.
- Review medications history to determine eligibility based on inclusion/exclusion criteria.

6.2.2 Enrollment / Baseline

After enrollment (according to section **4.4**) the following baseline evaluations will be conducted (but need not be repeated if dosing occurs within time-frame listed after screening evaluation):

- The baseline correlative studies as described in section **6.1.2** (obtained within 72 hrs of first dose).
- History and physical exam with vital signs and body weight (obtained within 7 days prior to dosing).
- Imaging studies (obtained within 28 days prior to dosing), studies selected based on location and type of disease, as per investigator discretion, to be used consistently throughout study for disease evaluation:
  - CT with contrast, or PET/CT scan of chest, abdomen and pelvis
  - MRI if unable to obtain CT scan
  - Other x-rays or scans at investigator discretion, based on location and type of disease.
- Baseline Laboratory Assessment (obtained within 72 hrs of first dose, if screening labs are > 72 hours from Cycle 1 Day 1)

  The tests should include at least the following:

  - CBC
  - Urinalysis
  - Electrolytes, BUN, creatinine
  - CEA level
  - Thyroid function: T3, T4, TSH

Results are not required prior to administration of first dose (Cycle 1 Day 1 only) of study drug. Baseline laboratory values obtained on Day 1 Cycle 1 will not affect the subject's eligibility to participate in the study after the subject has undergone Sponsor approval and enrolled on the study.

6.2.3 Treatment Evaluations

6.2.3.1 Day 1 of each cycle (after Cycle 1) prior to dosing

- A symptom directed physical exam and body weight should be performed prior to any dose at the discretion of the investigator, with vital signs as per section **5.1.5.1**
- Laboratory evaluation
  - Hematological profile: CBC with differential and platelet count, PT, aPTT, fibrinogen.
  - Biochemical profile: BUN, serum creatinine, uric acid, LDH, calcium, magnesium, sodium, chloride, potassium, carbon dioxide, alkaline phosphatase, total bilirubin, direct bilirubin, AST (SGOT), ALT (SGPT), protein, glucose and albumin, C-reactive

CONFIDENTIAL:
NOT TO BE DISCLOSED WITHOUT PERMISSION FROM PRECISION BIOLOGICS - FOR QUALIFIED INVESTIGATORS AND THEIR IRBS ONLY

protein.
- Correlative studies as described in Section **6.1.2**.
- Adverse/Serious Adverse Event reporting
- Concomitant medications documentation

6.2.3.2 Cycle 1: Daily for week 1

- Hematological profile: CBC

6.2.3.3 Cycle 1: Day 4, Day 8, Day 18 (+/- 48 hours):

- Vital signs (systolic and diastolic blood pressures, respiratory and heart rates, and temperature (°C)).
- Hematological profile: CBC with differential
- Adverse/Serious Adverse Event reporting
- Concomitant medications documentation

If there is a drop in hemoglobin level of ≥ 2 gm/dL from the hemoglobin level drawn prior to the dose of NEO-201, an evaluation for hemolysis must be conducted to include the following:

- Bilirubin, direct and indirect
- LDH
- Direct Antiglobin Test (DAT)/Coomb's Test

6.2.3.4 Day 14 (prior to dosing) of every cycle (+/- 48 hours) [Also serves as Day 1 if proceeding to another cycle]:

- A symptom directed physical exam should be performed prior to any dose at the discretion of the investigator, with vital signs as per section **5.1.5.1**.
- Laboratory evaluation
  - Hematological profile: CBC with differential and platelet count, PT/INR, aPTT, fibrinogen.
  - Biochemical profile: BUN, serum creatinine, uric acid, LDH, calcium, magnesium, sodium, chloride, potassium, carbon dioxide, alkaline phosphatase, total bilirubin, direct bilirubin, AST (SGOT), ALT (SGPT), protein, glucose and albumin, C-reactive protein.
  - Correlative studies as described in Section **6.1.2**.
- Adverse/Serious Adverse Event reporting
- Concomitant medications documentation

6.2.3.5 End of Cycle Evaluations (Day 28 ± 4 days)

- Clinical evaluation with performance status and vital signs and weight
- Laboratory evaluation:
  - Hematological profile: CBC with differential and platelet count, PT, aPTT, fibrinogen.
  - Biochemical profile: BUN, serum creatinine, uric acid, LDH, calcium,

CONFIDENTIAL:
NOT TO BE DISCLOSED WITHOUT PERMISSION FROM PRECISION BIOLOGICS - FOR QUALIFIED INVESTIGATORS AND THEIR IRBS ONLY

magnesium, sodium, chloride, potassium, carbon dioxide, alkaline phosphatase, total bilirubin, direct bilirubin, AST (SGOT), ALT (SGPT), protein, glucose and albumin, C-reactive protein.

- Adverse/Serious Adverse Event reporting
- Concomitant medications documentation

6.2.3.6 *End Cycle Evaluation-Even Cycles ONLY (Cycle 2, 4, 6, etc)*

In addition to the end of cycle evaluations in section **6.2.3.5,** the following evaluations will be performed:

- CEA levels: every 2 cycles.
- Correlative studies as described in Section **6.1.2**
- Imaging studies every 2 cycles
    - CT scan with contrast of chest, abdomen and pelvis; or PET/CT or MRI based on disease, or if subject cannot undergo CT scan.
    - Tumor evaluations should be conducted according to RECISTv1.1 guidelines (see section **16.4).**
    - Tumor marker evaluations (if applicable)

6.2.4 Follow-Up

If a subject is removed from study therapy for any reason other than progression of disease, subjects will be followed every 3 months for disease progression. At these visits, the following evaluations will be performed:

- Clinical evaluation with performance status and vital signs and weight
- Imaging studies
    - CT scan with contrast of chest, abdomen and pelvis; or PET/CT or MRI based on disease, or if subject cannot undergo CT scan.
    - Tumor evaluations should be conducted according to RECISTv1.1 guidelines (see section **16.4)**
- Adverse/Serious Adverse Event reporting
- Concomitant medications documentation

6.2.5 Final Study Visit

The final study visit will occur 30 days after the last dose of study agent or at the time of progression, whichever is later. If the subject cannot return to the participating site for this visit, a request will be made to collect required clinical labs from a local physician or laboratory. If this is not possible, subjects may be assessed by telephone for symptoms.

- History and physical exam with vital signs.
- Imaging studies (if not performed within the prior 28 days)
- Laboratory Assessment

  The tests should include the following:

  - Hematological profile: CBC with differential and platelet count, PT, aPTT, fibrinogen.
  - Biochemical profile: BUN, serum creatinine, uric acid, LDH, calcium, magnesium, sodium, chloride, potassium, carbon dioxide, alkaline phosphatase, total bilirubin, direct bilirubin, AST (SGOT), ALT (SGPT), protein, glucose and albumin, C-reactive protein
  - CEA level
  - Thyroid function: T3, T4, TSH

- Adverse/Serious Adverse Event reporting
- Concomitant medications documentation

6.2.6 Early Termination Visit

If a subject is taken off treatment prior to completing the end of a cycle or disease evaluation after Cycle 2, the following tests should be performed when feasible:

- History and physical exam with vital signs.
- Imaging studies (if not performed within the prior 28 days)
- Laboratory Assessment
  - Hematological profile: CBC with differential and platelet count, PT, aPTT, fibrinogen.
  - Biochemical profile: BUN, serum creatinine, uric acid, LDH, calcium, magnesium, sodium, chloride, potassium, carbon dioxide, alkaline phosphatase, total bilirubin, direct bilirubin, AST (SGOT), ALT (SGPT), protein, glucose and albumin, C-reactive protein
- Adverse/Serious Adverse Event reporting
- Concomitant medications documentation

6.2.7 Unscheduled Visit

If a subject returns to clinic or is hospitalized for any reason, clinical relevant evaluations should be conducted at the discretion of the investigator or treating physician. Data regarding the evaluations should be captured in an unscheduled visit evaluation to include at a minimum the following information:

- Adverse/Serious Adverse Event reporting
- Concomitant medications documentation

6.2.8 Schedule of Events Table

See Appendix C section **16.3**.

6.3 Concomitant Medications, Treatments, and Procedures

6.3.1 Prohibited Medications, Treatments, and Procedures

There are no prohibited medications on this study, although no other cancer-directed therapies or investigational agents may be administered while on this study.

6.3.2 Prophylactic Medications, Treatments, and Procedures

The following premedications will be administered prior to every dose of NEO-201, consistent with administration of monoclonal antibodies: dexamethasone (10 mg I.V.), diphenhydramine (25-50 mg I.V. or po) and ranitidine (50 mg I.V.) or equivalent (See Section 16.2 Appendix B). These premedications, dosages and schedule may be modified based on patient needs, as per investigator's discretion. Should signs/symptoms of infusion reaction/allergic reaction occur, subjects may be treated with rate of infusion decrease, and additional doses of diphenhydramine and/or dexamethasone, or comparable supportive medications. For subsequent infusions subjects will be premedicated prior to each infusion with dexamethasone, ranitidine and diphenhydramine 20-30 minutes prior to dosing of NEO-201 according to Section **16.2** Appendix B.

Symptomatic interventions may be performed (i.e. stent placement for biliary obstruction, thoracentesis or paracentesis for recurrent fluid accumulation), while participating on this trial, with a delay of up to one cycle (4 weeks between doses) without requiring study discontinuation.

6.3.3 Human Granulocyte Colony-stimulating Factor (G-CSF) and antibiotics

Decreased neutrophil levels may be treated with G-CSF, i.e. filgrastim. Because subjects enrolled in this study often have decreased marrow reserve due to multiple prior chemotherapies and a decrease in neutrophils is an expected toxicity of NEO-201 as was seen in the majority of animals in pre-clinical testing, support with G-CSF for the recovery of neutrophils will be provided to prevent risk of infection during dose escalation and expansion cohorts on this study. All potential subjects will be screened for recent infections or infectious exposures and any indications of a higher risk for infection or presence of indolent infectious sources will be thoroughly evaluated during screening. Subjects initiating study therapy will undergo CBC daily for the first week of cycle 1 and may be hospitalized for grade 3 or 4 neutropenia based on recommendations of Infectious Disease (ID) Consult. Antibiotics will be administered under the direction of the ID Consult.

7 ASSESSMENT OF SAFETY

7.1 Specification of Safety Parameters

7.1.1 Definition of Adverse Event (AE)

An adverse event is defined as any reaction, side effect, or untoward event that occurs during the course of the clinical trial associated with the use of a drug in humans, whether or not the event is considered related to the treatment or clinically significant. For this study, AEs will include events reported by the subject, as well as clinically significant abnormal findings on physical examination or laboratory evaluation. A new illness, symptom, sign or clinically significant laboratory

abnormality or worsening of a pre-existing condition or abnormality is considered an AE. All AEs regardless of attribution must be recorded on the AE case report form.

All AEs, including clinically significant abnormal findings on laboratory evaluations, regardless of severity, will be followed until return to baseline or stabilization of event. Serious adverse events that occur more than 30 days after the last administration of investigational agent/intervention and have an attribution of at least possibly related to the agent/intervention should be recorded and reported.

An abnormal laboratory value will be considered an AE if the laboratory abnormality is characterized by any of the following:

- Results in discontinuation from the study
- Is associated with clinical signs or symptoms
- Requires treatment or any other therapeutic intervention
- Is associated with death or another serious adverse event, including hospitalization.
- Is judged by the Investigator to be of significant clinical impact
- If any abnormal laboratory result is considered clinically significant, the investigator will provide details about the action taken with respect to the test drug and about the subject's outcome.

7.1.2 Definition of Serious Adverse Events (SAE)

An adverse event or suspected adverse reaction is considered serious if in the view of the investigator or the sponsor, it results in any of the following:

- Death,
- A life-threatening adverse drug experience
- Inpatient hospitalization or prolongation of existing hospitalization (except for planned hospitalizations for grade 3 or 4 neutropenia, elective procedures, such as PK blood draws)
- Persistent or significant incapacity or substantial disruption of the ability to conduct normal life functions
- A congenital anomaly/birth defect.
- Important medical events that may not result in death, be life-threatening, or require hospitalization may be considered a serious adverse drug experience when, based upon appropriate medical judgment, they may jeopardize the patient or subject and may require medical or surgical intervention to prevent one of the outcomes listed in this definition.

7.1.2.1 Disability

A substantial disruption of a person's ability to conduct normal life functions.

7.1.3 Definition of Suspected Adverse Event

Suspected adverse reaction means any adverse event for which there is a reasonable possibility that the drug caused the adverse event. For the purposes of IND safety reporting, 'reasonable

possibility' means there is evidence to suggest a causal relationship between the drug and the adverse event. A suspected adverse reaction implies a lesser degree of certainty about causality than adverse reaction, which means any adverse event caused by a drug.

7.1.4 Definition of Unanticipated Problems (UP)

Any incident, experience, or outcome that:

- Is unexpected in terms of nature, severity, or frequency in relation to

  (a) the research risks that are described in the IRB-approved research protocol and informed consent document; Investigator's Brochure or other study documents, and

  (b) the characteristics of the subject population being studied; **AND**
- Is related or possibly related to participation in the research; **AND**
- Suggests that the research places subjects or others at a *greater risk of harm* (including physical, psychological, economic, or social harm) than was previously known or recognized.

7.1.5 Definition of unexpected adverse reaction

An adverse event or suspected adverse reaction is considered "unexpected" if it is not listed in the investigator brochure or is not listed at the specificity or severity that has been observed; or, if an investigator brochure is not required or available, is not consistent with the risk information described in the general investigational plan or elsewhere in the current application. "Unexpected" also refers to adverse events or suspected adverse reactions that are mentioned in the investigator brochure as occurring with a class of drugs or as anticipated from the pharmacological properties of the drug, but are not specifically mentioned as occurring with the particular drug under investigation.

7.1.6 Definition of Serious

An Unanticipated Problem or Protocol Deviation is serious if it meets the definition of a Serious Adverse Event or if it compromises the safety, welfare or rights of subjects or others.

7.2 CLASSIFICATION OF AN ADVERSE EVENT

7.2.1 Severity of Event

The grading scales found in the revised NCI Common Terminology Criteria for Adverse Events (CTCAE) version 5.0 will be utilized for AE reporting. All appropriate treatment areas should have access to a copy of the CTCAE version 5.0. A copy of the CTCAE version 5.0 can be downloaded from the CTEP web site http://ctep.cancer.gov/protocolDevelopment/electronic_applications/ctc.htm. If an AE is not specified in the CTC, the grading of the severity will be done according to the following descriptions:

- Grade 1 Mild; asymptomatic or mild symptoms; clinical or diagnostic observations only; intervention not indicated.
- Grade 2 Moderate; minimal, local or noninvasive intervention indicated; limiting age-appropriate instrumental ADL*.
- Grade 3 Severe or medically significant but not immediately life-threatening; hospitalization or prolongation of hospitalization indicated; disabling; limiting self care ADL**.
- Grade 4 Life-threatening consequences; urgent intervention indicated.
- Grade 5 Death related to AE.

7.2.2 Relationship to Study Agent

The relationship to study drug or attribution of the AE will be assessed as follows:

- Definite – The AE *is clearly related* to the study treatment.
- Probable – The AE *is likely related* to the study treatment.
- Possible – The AE *may be related* to the study treatment.
- Unlikely – The AE *is doubtfully related* to the study treatment.
- Unrelated – The AE *is clearly NOT related* to the study treatment.

7.2.3 Expectedness

All AEs will be evaluated as to whether they are expected or unexpected.

7.3 TIME PERIOD AND FREQUENCY FOR EVENT ASSESSMENT AND FOLLOW UP

The occurrence of an AE or SAE may come to the attention of study personnel during study visits and interviews of a study participant presenting for medical care, or upon review by a study monitor. At each study visit, the investigator will inquire about the occurrence of AE/SAEs since the last visit. Events will be followed for outcome information until resolution or stabilization.

All AEs including local and systemic reactions not meeting the criteria for SAEs will be captured on the appropriate CRF in the EDC. Information to be collected includes event description, time of onset, clinician's assessment of severity, relationship to study product (assessed only by those with the training and authority to make a diagnosis), and time of resolution/stabilization of the event. All AEs occurring while on study must be documented appropriately regardless of relationship. All AEs will be followed to adequate resolution. Subjects will be assessed and AEs, SAEs and deviations documented from the initiation of the first dose of NEO-201 until 30 days after the last dose of NEO-201. Serious adverse events that occur more than 30 days after the last administration of investigational agent/intervention and have an attribution of at least possibly related to the agent/intervention should be recorded and reported.

Any medical condition that is present at the time that the participant is screened will be considered as baseline and not reported as an AE. However, if the study participant's condition deteriorates at any time during the study, it will be recorded as an AE. UPs will be recorded in the data collection system throughout the study.

7.4 REPORTING PROCEDURES

7.4.1 Adverse Event Reporting

Adverse event data collection and reporting, which are required as part of every clinical trial, are done to ensure the safety of subjects enrolled in this study as well as those who will enroll in future studies using similar agents. AEs are reported in a routine manner at scheduled times during the trial using the Sponsor provided electronic database.

7.4.2 Serious Adverse Event Reporting

An investigator must report all SAEs to the Sponsor immediately after the investigator becomes aware of the event, using the mandatory MedWatch form 3500a or institutional SAE Report Form. Any serious adverse event whether or not considered drug related, including those listed in the protocol or investigator brochure and must include an assessment of whether there is a reasonable possibility that the drug caused the event.

Study endpoints that are serious adverse events, such as those meeting the definition of DLT, must be reported to the Sponsor immediately after the investigator becomes aware of the event. In addition, the investigator must immediately report the all deaths to the sponsor, including those not attributed to the study drug.

All deaths and life-threatening events, whether related or unrelated, will be recorded on MedWatch Form 3500A or institutional SAE Report form, and submitted to the Study Sponsor **within 24 hours** of site awareness.

Sponsor contact information: Philip.Arlen@precision-biologics.com

All SAEs will be followed until satisfactory resolution or until the site investigator deems the event to be chronic or the adherence to be stable. Other supporting documentation of the event may be requested by the Study Sponsor and should be provided as soon as possible.

The Study Sponsor will be responsible for notifying FDA of any unexpected fatal or life-threatening suspected adverse reaction as soon as possible but in no case later than 7 calendar days after the sponsor's initial receipt of the information.

7.4.3 Deviation Reporting

All deviations reported to the local IRB, must be reported to the Sponsor within 1 week of reporting to the IRB, regardless of rationale or justification. Deviations can be reported by completing the Protocol Deviation Report form and sending by e-mail to:

Precision Biologics, Inc.
Phil Arlen, M.D.
Tel: 301-500-8648 (office)

Philip.Arlen@Precision-Biologics.com

7.4.4 Unanticipated Problem Reporting

An investigator must report all unanticipated problems to the sponsor **immediately,** but no greater than 7 days after the investigator becomes aware of the event, using the Sponsor provided EDC, and must include an assessment of whether there is a reasonable possibility that the drug caused the event.

7.4.5 Reporting of Pregnancy

7.4.5.1 Maternal exposure

If a subject becomes pregnant during the course of the study, the study treatment should be discontinued immediately and the pregnancy reported to the Sponsor. The potential risk of exposure of the fetus to the investigational agent(s) or chemotherapy agents (s) should be documented in box B5 of the MedWatch form "Describe Event or Problem".

Pregnancy itself is not regarded as an AE unless there is a suspicion that the study treatment under study may have interfered with the effectiveness of a contraceptive medication. However, as subjects who become pregnant on study risk intrauterine exposure of the fetus to agents which may be teratogenic, the CCR is requesting that pregnancy should be reported in an expedited manner as **Grade 3** "***Pregnancy, puerperium and perinatal conditions - Other (pregnancy)***" under the ***Pregnancy, puerperium and perinatal conditions*** SOC.

Congenital abnormalities or birth defects and spontaneous miscarriages should be reported and handled as SAEs. Elective abortions without complications should not be handled as AEs. The outcome of all pregnancies (spontaneous miscarriage, elective termination, ectopic pregnancy, normal birth, or congenital abnormality) should be followed up and documented.

If any pregnancy occurs in the course of the study, then the investigator should inform the Sponsor within 1 day, i.e., immediately, but **no later than 24 hours** of when he or she becomes aware of it.

The designated Sponsor representative will work with the investigator to ensure that all relevant information is provided to the Sponsor within 1 to 5 calendar days for SAEs and within 30 days for all other pregnancies.

The same timelines apply when outcome information is available.

7.4.5.2 Paternal exposure

Male subjects should refrain from fathering a child or donating sperm during the study and for 14 after the last dose of NEO-201.

Pregnancy of the subject's partner is not considered to be an AE. However, the outcome of all pregnancies (spontaneous miscarriage, elective termination, ectopic pregnancy, normal birth, or congenital abnormality) occurring from the date of the first dose until 14 days after the last dose should, if possible, be followed up and documented.

CONFIDENTIAL:
NOT TO BE DISCLOSED WITHOUT PERMISSION FROM PRECISION BIOLOGICS - FOR QUALIFIED INVESTIGATORS AND THEIR IRBS ONLY

7.5 STUDY HALTING RULES

The study accrual may be temporarily suspended in order to allow for comprehensive review of the data by the Principal Investigators and Sponsor with FDA input, to consider possible amendment of the protocol for any of the following events that occur in a study subject:

- A Grade 3 or 4 hemolytic event after administration of NEO-201;
- Any Grade 4 or 5 toxicity by the NCI-CTCAE (Version 5.0) suspected of relationship to NEO-201 with the exception of non-complicated laboratory abnormalities, such as neutropenia or anemia; or
- MTD is exceeded at the first dose level.

7.6 SAFETY OVERSIGHT

7.6.1 Principal Investigator/Research Team

The sponsor will meet with the clinical research teams on a regular basis every 1-2 weeks when subjects are being actively treated on the Phase 1 portion of the trial to discuss each subject. Decisions about dose level enrollment and dose escalation if applicable will be made based on the toxicity data from prior subjects.

All data will be collected in a timely manner and reviewed by the principal investigator or a lead associate investigator. Adverse events will be reported as required above. Any safety concerns, new information that might affect either the ethical and or scientific conduct of the trial, or protocol deviations will be immediately reported to the IRB using EDC and to the Sponsor.

The principal investigator will review adverse event and response data on each subject to ensure safety and data accuracy. The principal investigator will personally conduct or supervise the investigation and provide appropriate delegation of responsibilities to other members of the research staff.

8 CLINICAL MONITORING

8.1 GOOD CLINICAL PRACTICE

The study will be conducted in accordance with the International Conference on Harmonization (ICH) for Good Clinical Practice (GCP) and the appropriate regulatory requirement(s). The Investigator will be thoroughly familiar with the appropriate use of the study drug as described in the protocol and Investigator’s Brochure. Essential clinical documents and electronic files will be maintained to demonstrate the validity of the study and the integrity of data collected. Master files should be established at the beginning of the study, maintained for duration of the study, and retained according to appropriate regulations. The Investigator's Study File will contain the protocol/amendments, Case Report and Query Forms, Institutional Review Board and governmental approval with correspondence, sample informed consent, sponsor correspondence records, staff curriculum vitae and authorization forms, delegation logs, screening and enrollment logs and other appropriate documents/correspondence etc.

CONFIDENTIAL:
NOT TO BE DISCLOSED WITHOUT PERMISSION FROM PRECISION BIOLOGICS - FOR QUALIFIED INVESTIGATORS AND THEIR IRBS ONLY

8.2 Ethical Considerations

The study will be conducted in accordance with ethical principles founded in the Declaration of Helsinki. The Institutional Review Board/Independent Ethics Committee (IRB/IEC) will review all appropriate study documentation in order to safeguard the rights, safety and well-being of the subjects. The study will be conducted only at sites where IRB/IEC approval has been obtained. The protocol, Investigational Drug Brochure, informed consent, advertisements (if applicable), written information given to the subjects, safety updates, annual progress reports, and any revisions to these documents will be provided to the IRB/IEC by the Investigator.

8.3 Subject Information and Informed Consent

After the study has been fully explained, written informed consent will be obtained from either the subject or his/her guardian or legal representative prior to study participation. The method of obtaining and documenting the informed consent and the contents of the consent will comply with ICH-GCP and all applicable regulatory requirement(s).

8.4 Subject Confidentiality

In order to maintain subject privacy, data capture tools, study drug accountability records, study reports, research blood tubes and communications will identify the subject only by a unique code number assigned to the subject. The Investigator will grant monitor(s) and auditor(s) from Precision Biologics or its designee and regulatory authority(ies) access to the subject's original medical records, electronic medical records or certified copies for verification of data gathered and to audit the data collection process. The subject's confidentiality will be maintained and will not be made publicly available to the extent permitted by the applicable laws and regulations.

8.5 Protocol Compliance

The Investigator will conduct the trial in compliance with the protocol provided by Precision Biologics and given approval/favorable opinion by the IRB/IEC and the appropriate regulatory authority(ies). Modifications to the protocol may not be made without agreement of both the Investigator and Precision Biologics. Changes to the protocol will require written IRB/IEC approval/favorable opinion prior to implementation, except when the modification is needed to eliminate an immediate hazard(s) to subjects. The IRB/IEC may provide, if applicable regulatory authority(ies) permit, expedited review and approval/favorable opinion for minor change(s) in ongoing trials that have the approval /favorable opinion of the IRB/IEC. Precision Biologics will submit all protocol modifications to the regulatory authority(ies) in accordance with the governing regulations.

When immediate deviation from the protocol is required to eliminate an immediate hazard(s) to subjects, the Investigator will contact Precision if circumstances permit, to discuss the planned course of action. Any departures from the protocol must be fully documented in the source documentation.

Study drug will be administered only to eligible subjects under the supervision of the Investigator or identified sub-investigator(s). The pharmacist will maintain records of study drug receipt and

dispensing, including the applicable identification numbers. Any discrepancy between the calculated dose and dose administered and the reason for the discrepancy are to be recorded.

8.6 DATA COLLECTION AND ENTRY

Precision or its designee will instruct the participating sites regarding data capture procedures. It is the Investigator's responsibility to ensure the accuracy, completeness, and timeliness of the data reported for each subject.

8.7 SPONSOR STUDY MONITORING

Clinical site monitoring is conducted to ensure that the rights and well-being of human subjects are protected, that the reported trial data are accurate, complete, and verifiable, and that the conduct of the trial is in compliance with the currently approved protocol/amendment(s), with GCP, and with applicable regulatory requirement(s).

Monitoring and auditing procedures developed by Precision Biologics Inc., or its designee will be followed, in order to comply with GCP guidelines. The Sponsor will review all serious adverse events and will monitor the data and toxicities to identify trends at least weekly during treatment of subjects. The participating sites' PI(s) and the coordinating sponsor will discuss all toxicities to determine DLTs and dose escalation decisions. In the event of a DLT, the participating site PIs will be notified within 24 hours of the nature of the event. The Study Sponsor will be responsible for revising the protocol as needed to maintain safety.

The study will be monitored by Precision Biologics, Inc. or its designee on a regular basis throughout the study period. Monitoring will be done by personal visits from a representative of the sponsor (site monitor) who will review the source documents. On-site checking of case report forms for completeness and clarity and consistency as compared with source documents, and clarification of administrative matters will be performed. Source documentation includes but is not limited to the subject's clinic and/or office chart, hospital chart, informed consent forms, treatment notes, laboratory reports, pharmacy records, radiographs, and any other records maintained to conduct and evaluate the clinical study. The investigator must ensure the accuracy and completeness of the data reported, and its consistency with the source documentation. The primary source document for this study will be the subject's medical records. If separate research records are maintained by the investigator(s) both the medical record and the research records will be monitored/audited for the purposes of the study. The site monitor will ensure that the investigation is conducted according to protocol design and regulatory requirements by frequent communications (letter, telephone, and fax).

All unused study drug and other study materials are to be returned to Precision Biologics, Inc. after the clinical phase of the study has been completed.

8.8 ON-SITE AUDITS

Regulatory authorities, the IEC/IRB, and/or Precision Biologics's clinical quality assurance group may request access to all source documents, printouts of any data entry forms, and other study documentation for on-site audit or inspection. Direct access to these documents must be guaranteed by the Investigator, who must provide support at all times for these activities.

8.9 DATA ENTRY COMPLETION

For subjects who provide informed consent and were not entered/randomized into the study, the reason the subject was not entered/randomized, i.e., did not meet one or more inclusion criteria, met one or more exclusion criteria, or other (e.g., lost to follow up, consent withdrawn), will also be collected and maintained on the Screening/Study Log.

It is the Investigator's responsibility to ensure the accuracy, completeness, and timeliness of the data reported for each subject. Source documentation supporting the data should indicate the subject's participation in the study and should document the dates and details of study procedures, adverse events, and subject status.

The Investigator, or designated representative, should complete the data entry as soon as possible after information is collected, preferably within 2 weeks of study visit. Any outstanding entries must be completed immediately after the final examination. An explanation should be given for all missing data.

The Investigator's Study File will contain documents as outlined in ICH ECP E6 Section 8, including the protocol/amendments, Data Query Forms, Institutional Review Board and governmental approval with correspondence, sample informed consent, sponsor correspondence records, staff curriculum vitae and authorization forms and other appropriate documents/correspondence, etc.

8.10 RECORD RETENTION

The Investigator will maintain all study records according to ICH-GCP and applicable regulatory requirement(s). Study documents (including subject records, copies of collected data, study notebook, and pharmacy records) must be kept secured in accordance with Precision Biologics policies and applicable regulatory requirements for a period of 2 years following marketing of NEO-201 or for 2 years after centers have been notified that the study has been closed and the IND has been discontinued. There may be other circumstances for which Precision Biologics is required to maintain study records and, therefore, Precision Biologics should be contacted prior to removing study records for any reason. If the Investigator withdraws from the responsibility of keeping the study records, custody must be transferred to a person willing to accept the responsibility. Precision Biologics Inc. must be notified in writing if a custodial change occurs.

9 STATISTICAL CONSIDERATIONS

9.1 STATISTICAL AND ANALYTICAL PLAN

A formal statistical analysis plan (SAP) will be prepared and finalized before database lock for the final analysis for the study report. The SAP will provide details regarding the definition of analysis subjects (populations), analysis variables, and analysis methodology to meet all study objectives.

The principle and key elements of the SAP are provided as follows:

- In general, safety and efficacy data will be summarized with descriptive statistics, including means, standard deviations, medians, minimums and maximums for continuous variables, the number of subjects and percent in each category for categorical variables.

- Data from each individual will be tabulated as appropriate. Efficacy and safety endpoints will be tabulated by dose cohort and time point.

9.2 Statistical Hypotheses

The primary analytic focus for this study is to determine and describe the safety profile of the investigational agent (NEO-201) at 4 escalating dose levels. Descriptive statistics for safety endpoints will be presented for each dose cohort. Examination of any potential dose-response relationship with respect to safety outcomes will also be assessed. Since the study is not designed to provide adequate power for hypothesis testing related to clinical activity, the analytic focus for both the primary and secondary endpoints will be descriptive. Although hypothesis testing will be conducted for clinical activity, the interpretation will be considered exploratory.

9.3 Analysis Datasets

The primary dataset will be the subset of participants for whom safety analyses can be completed, that is, any subject who received at least one dose (or partial dose) of NEO-201. Data will be descriptively displayed to include demographics, prior medical therapies, performance status, treatment exposure, adverse events (treatment-emergent and treatment-related adverse events), serious adverse events, DLTs, IHC, PKs, and immune responses (including HAHA, functional and phenotypic immune processes and serum cytokine, chemokine and soluble factors), and best overall response at C2D28.

9.4 Description of Statistical Methods

9.4.1 General Approach

Statistical analyses will be descriptive in nature. Descriptive statistics will consist of the number and percentage of subjects in each category for discrete variables, and the sample size, mean, median, S.D., minimum, and maximum for continuous variables. All mean and median values will be formatted to one more decimal place than the measured value. Standard deviation values will be formatted to two more decimal places than the measured value. All percentages will be rounded to whole numbers. The number and percentage of responses will be presented in the form XX (XX%), where the percentage is in the parentheses. Confidence intervals (CIs) will be presented as 2-sided 95% CIs. All listings will be sorted for presentation in order of study phase, dose level, study center, subject, and date of procedure or event. All analysis and summary tables will have the analysis population sample size (i.e., number of subjects).

When necessary for analysis purposes, an adverse event or medication start or stop dates without a specific day of the month given (i.e., JAN1999) will be assigned the 15th day of the month and dates without a specific day or month (i.e., 1999) will be assigned the 30th day of June to complete the date. If the incomplete date is a start date and the above imputation inappropriately results in a date on or before the first dose date, then the incomplete date will be assigned to the day following the first dose date. If either imputation results in an imputed start date after the stop date, then the start date will be set to the day prior to the stop date.

CONFIDENTIAL:
NOT TO BE DISCLOSED WITHOUT PERMISSION FROM PRECISION BIOLOGICS - FOR QUALIFIED INVESTIGATORS AND THEIR IRBS ONLY

When necessary for analysis purposes, disease progression dates without a specific day of the month given (i.e., JAN1999) will be assigned the first day of the month and dates without a specific day or month (i.e., 1999) will be evaluated as appropriate. If the above imputation inappropriately results in a date on or before the last date known to be progression free, then the incomplete date will be assigned to the day following the last date known to be progression free.

The day of the first dose of any study drug will be defined as Day 1.

Baseline value will be defined as the last value before the first dose of any study drug is administered.

Final Evaluation will be defined as the last on-treatment value.

MedDRA version 20.0 will be used for adverse events coding.

9.4.2 Analysis of the Primary Endpoint

The safety of NEO-201 will be assessed by:

- Treatment-emergent adverse events.
- Treatment-emergent serious adverse events.
- Changes in clinical laboratory tests (clinical chemistry, hematology).
- Changes in vital signs (blood pressure, pulse, respiratory rate and body temperature).
- Changes in physical exams. Signs and symptoms assessed may require additional testing as clinically indicated such as ECG, PFT, radiographic studies, etc.

Safety data will be analyzed per standard methods and interpreted descriptively. Safety data will be summarized for each dose cohort separately and for all dose cohorts combined. Adverse events will be assessed using the CTCAE version 5.0 for type and severity of event. Serious Adverse Events will be summarized for each dose cohort and for all dose cohorts combined. Reasons for discontinuation will be tabulated.

Laboratory includes hematology, serum chemistry, and urinalysis; laboratory collected prior to NEO-201 infusion will be the baseline laboratory. The study will utilize local lab for all clinical laboratory testing. Laboratory data will be tabulated based on the following result class.

- Normal: result is within the local lab normal range
- Abnormal: result is either higher or lower than the normal range

All abnormal values will be assessed for clinical significance; clinical significance will be captured in the case report form. Number and percent of subjects within each result class will be tabulated by time point for each lab test without formal inferential statistics. If data permits, shift in result class from baseline to post baseline may also be tabulated.

Vital signs collected immediately prior to receiving study drug will be the baseline vital signs. Observed vital sign values and change from baseline in vital signs at each visit will be summarized without formal statistical testing.

Vital sign result may also be tabulated based on the following result class.

- Normal: result is within the normal range

CONFIDENTIAL:
NOT TO BE DISCLOSED WITHOUT PERMISSION FROM PRECISION BIOLOGICS - FOR QUALIFIED INVESTIGATORS AND THEIR IRBS ONLY

- Abnormal: result is either higher or lower than the normal range

All abnormal values will be assessed for clinical significance; clinical significance will be captured in the case report form. Number and percent of subjects within each result class will be tabulated by time point for each vital sign.

Findings of physical examinations will be tabulated by treatment groups and abnormal values will be assessed for clinical significance; clinical significance will be captured in the case report form.

9.4.3 Analysis of the Secondary Endpoints

Analysis of IHC, PK, immunogenicity and ORR will be conducted as outlined in Section **6.1.2**. All results will be reported descriptively.

9.4.4 Planned Interim Analyses

There is no planned interim analysis during the Phase I portion of the study. Prior to initiating the dose expansion cohorts, once MTD is determined, the following data will be evaluated per tumor group:

- IHC evaluations of tumor samples including percentage of tumor cells staining with NEO-201 and staining intensity. When available analysis of the IHC results will be correlated with the characteristics of enrolled subjects, including tumor type, response to NEO-201, and occurrence of adverse events, to aid in the design of the expansion cohorts, which will be added to this study with an amendment.
- Any evidence of clinical response according to RECIST v1.1 will be used to describe response. Evaluable subjects will be assigned one of the following categories: 1) Complete Response; 2) Partial Response; 3) Stable Disease; 4) Progressive Disease; and 5) not evaluable (defined as death from malignant disease, death from toxicity, death due to other causes, or unknown-not assessable, insufficient data, unable to complete at least two doses of NEO-201).
- Results of immunologic correlates will be reported descriptively, with means, medians, ranges, where appropriate, and when available will be correlated with clinical factors including response. The analysis of these results will be hypothesis generating and may be expanded in the expansion cohorts for further analysis.

This analysis will be used to select expansion cohorts for the Expansion Phase of this study. The expansion phase of this study will be clearly defined in an amendment to this study. Selection of expansion cohorts may include subjects with colorectal cancer, pancreatic cancer, adenocarcinoma of the lung, squamous cell lung cancer, breast cancer, and mucinous and signet cell ovarian cancer. No additional subjects will be enrolled after Phase 1 until such protocol amendment is approved by the IRB and FDA.

9.4.5 Efficacy Review

Clinical responses will be evaluated every 8 weeks, or every 2 cycles while the subject is receiving NEO-201. Responses will be evaluated according to section **16.4** Appendix D. As the efficacy

determination during the Phase 1 enrollment of this study is a secondary outcome, there will be no central review of the clinical response determinations.

9.5 Sample Size

To complete the primary objective, 3 to 6 subjects will be enrolled in each of the 5 dose cohorts to a maximum of 30 subjects to determine MTD. If one of the additional dose cohorts is added (either 3 mg/kg or 5 mg/kg), an additional 6 subjects may be enrolled. The maximum (6 + 6 + 6+ 6 + 6 +6) of 36 subjects may be enrolled in the Phase I study. The study will allow for up to a total of 5 additional subjects to be replaced in the event a subject becomes inevaluable for MTD and requires replacement in the protocol numbers. The maximum accrual for the phase 1 portion of this protocol will be 36 +5 for a total of 41 subjects. It is expected that with accrual of 3-4 subjects per month, the phase 1 portion of this study will be completed in 18 months.

9.6 Measures to Minimize Bias

9.6.1 Enrollment/Randomization/Masking Procedures

Enrollment will be conducted on a first-come, first-serve basis. There will be no randomization or blinding procedures on this study. All care will be taken to minimize side effects, but they can be unpredictable in nature and severity. This study may involve currently unforeseeable risks to subjects. All subjects will have blood tests, examinations and CT scans of the chest/abdomen/pelvis as described in the monitoring schedule. Subjects will also be required to have a local physician to provide long-term care and to monitor for complications. If subjects suffer any physical injury as a result of participation in this study, immediate medical treatment is available at the participating institution. Although no compensation is available, any injury will be evaluated and treated in keeping with the benefits or care to which subjects are entitled under applicable regulations.

10 SOURCE DOCUMENTS AND ACCESS TO SOURCE DATA/DOCUMENTS

Source data are all information, original records of clinical findings, observations, or other activities in a clinical trial necessary for the reconstruction and evaluation of the trial. Source documentation supporting the data should indicate the subject's participation in the study and should document the dates and details of study procedures, adverse events, and subject status. Data recorded on source documents will be transcribed onto a validated data collection instrument (an EDC) provided by Precision Biologics or designee. The investigator must ensure the accuracy and completeness of the data reported, and its consistency with the source documentation. The primary source documents for this study will be the subject's medical records, progress notes, or research team notes in the subject's research file. If separate research records are maintained by the investigator(s) both the medical record and the research records will be monitored/audited for the purposes of the study.

CONFIDENTIAL:
NOT TO BE DISCLOSED WITHOUT PERMISSION FROM PRECISION BIOLOGICS - FOR QUALIFIED INVESTIGATORS AND THEIR IRBS ONLY

11 QUALITY ASSURANCE (QA) AND QUALITY CONTROL (QC)

QC procedures will be implemented beginning with the data entry system and data QC checks that will be run on the database will be generated. Any missing data or data anomalies will be communicated to the site(s) for clarification/resolution.

Following written SOPs, the monitors will verify that the clinical trial is conducted and data are generated, documented (recorded), and reported in compliance with the protocol, GCP, and the applicable regulatory requirements (e.g., Good Laboratory Practices (GLP), Good Manufacturing Practices (GMP)).

The investigational site will provide direct access to all trial related sites, source data/documents, and reports for the purpose of monitoring and auditing by the sponsor, and inspection by local and regulatory authorities.

12 ETHICS/PROTECTION OF HUMAN SUBJECTS

12.1 ETHICAL STANDARD

The investigator will ensure that this study is conducted in full conformity with Regulations for the Protection of Human Subjects of Research codified in 45 CFR Part 46, 21 CFR Part 50, 21 CFR Part 56, and/or the ICH E6

12.1.1 Selection Based on Gender, Ethnicity, and Race

Subjects from all racial/ethnic groups and both genders are eligible for this study if they meet the eligibility criteria. To date, no information suggests that differences in drug metabolism, immune response or disease response would be expected in one group compared with another. Efforts will be made to extend accrual to a representative population, but in this preliminary study, a balance must be struck between subject safety considerations and limitations on the number of individuals exposed to potentially toxic and/or ineffective treatments on one hand and the need to explore gender and ethnic aspects of clinical research on the other hand. If differences in outcome that correlate with ethnic identity are noted, accrual may be expanded or a follow-up study may be written to investigate those differences more fully.

12.1.2 Justification for Exclusions

Pregnant or lactating women are excluded due to unknown side effects from this antibody.

12.1.3 Participation of Children

Individuals under the age of 18 will not be eligible for participation in this study based on the fact that the safety profile of NEO-201 is unknown and the targeted diseases are rare or non-existent in children.

12.2 INSTITUTIONAL REVIEW BOARD

The protocol, informed consent form(s), recruitment materials, and all participant materials will be submitted to the IRB for review and approval. Approval of both the protocol and the consent form must be obtained before any participant is enrolled. Any amendment to the protocol will

require review and approval by the IRB before the changes are implemented to the study. All changes to the consent form will be IRB approved; a determination will be made regarding whether previously consented participants need to be re-consented

12.3 Informed Consent Process

12.3.1 Consent and Other Informational Documents Provided to Participants

A consent form describing in detail the study agent, study procedures, and risks are given to the participant and written documentation of informed consent is required prior to starting intervention/administering study product. The following consent materials are submitted with this protocol: consent template.

12.3.2 Consent Procedures and Documentation

Informed consent is a process that is initiated prior to the individual's agreeing to participate in the study and continues throughout the individual's study participation. Extensive discussion of risks and possible benefits of participation will be provided to the participants and their families. Consent forms will be IRB-approved and the participant will be asked to read and review the document. The investigator will explain the research study to the participant and answer any questions that may arise. All participants will receive a verbal explanation in terms suited to their comprehension of the purposes, procedures, and potential risks of the study and of their rights as research participants. Participants will have the opportunity to carefully review the written consent form and ask questions prior to signing. The participants should have the opportunity to discuss the study with their surrogates or think about it prior to agreeing to participate. The participant will sign the informed consent document prior to any procedures being done specifically for the study. The participants may withdraw consent at any time throughout the course of the trial. A copy of the informed consent document will be given to the participants for their records. The rights and welfare of the participants will be protected by emphasizing to them that the quality of their medical care will not be adversely affected if they decline to participate in this study.

12.3.2.1 Informed consent of non-English speaking subjects:

If there is an unexpected enrollment of a research participant for whom there is no translated extant IRB approved consent document, the principal investigator and/or those authorized to obtain informed consent will follow the institutional policy for unexpected enrollment of non-English speaking subjects. The summary that will be used is the English version of the extant IRB approved consent document. A copy of both the English version and the IRB approved short form consent will be maintained in the subject's medical record.

Unless the PI is fluent in the prospective subject's language, an interpreter will be present to facilitate the conversation (using either the long translated form or the short form). Preferably someone who is independent of the subject (i.e., not a family member) will assist in presenting information and obtaining consent. Whenever possible, interpreters will be provided copies of the relevant consent documents well before the consent conversation with the subject (24 to 48 hours if possible).

CONFIDENTIAL:
NOT TO BE DISCLOSED WITHOUT PERMISSION FROM PRECISION BIOLOGICS - FOR QUALIFIED INVESTIGATORS AND THEIR IRBS ONLY

12.4 PARTICIPANT AND DATA CONFIDENTIALITY

Participant confidentiality is strictly held in trust by the participating investigators, their staff, and the sponsor(s) and their agents. This confidentiality is extended to cover testing of biological samples and genetic tests in addition to the clinical information relating to participants. Therefore, the study protocol, documentation, data, and all other information generated will be held in strict confidence. No information concerning the study or the data will be released to any unauthorized third party without prior written approval of the sponsor. The principal investigator will maintain a subject list with subject identifiers and the Sponsor assigned study ID number, which allows only the research study team at the participating site access to participant data. All CRFs, data reports, logs and records will be identified by the study ID number only.

The study monitor, other authorized representatives of the sponsor, representatives of the IRB or pharmaceutical company supplying study product may inspect all documents and records required to be maintained by the investigator, including but not limited to, medical records (office, clinic, or hospital) and pharmacy records for the participants in this study. The clinical study site will permit access to such records.

The study participant's contact information will be securely stored at each clinical site for internal use during the study. At the end of the study, all records will continue to be kept in a secure location for as long a period as dictated by local IRB and Institutional regulations.

Study participant research data, which is for purposes of statistical analysis and scientific reporting, will be transmitted to and stored at the participating site. This will not include the participant's contact or identifying information. Rather, individual participants and their research data will be identified by a unique study identification number. The study data entry and study management systems used by clinical sites and research staff will be secured and password protected. At the end of the study, all study databases will be de-identified and archived at Precision Biologics, Inc.

12.4.1 Research Use of Stored Human Samples, Specimens or Data

Samples and data collected under this protocol may be used to study the clinical conditions and treatment regimen outlined in this study. No genetic testing will be performed

All data will be kept secure. The PI will be responsible for overseeing entry of data into the Sponsor's password protected electronic system and ensuring data accuracy, consistency and timeliness. The principal investigator, associate investigators/research nurses and/or a data manager will assist with the data management efforts. All human subjects personally identifiable information (PII) as defined in accordance to the Health Insurance Portability and Accountability Act, eligibility and consent verification will be recorded. Primary data obtained during the conduct of the protocol will be kept in secure network drives or in approved alternative sites that comply with all relevant local, state and federal security standards. Primary and final analyzed data will have identifiers so that research data can be attributed to an individual human subject participant.

End of study procedures: Data will be stored according to HHS and FDA regulations as applicable.

Loss or destruction of data: Should we become aware that a major breech in our plan to protect subject confidentiality and trial data has occurred, the IRB will be notified.

CONFIDENTIAL:
NOT TO BE DISCLOSED WITHOUT PERMISSION FROM PRECISION BIOLOGICS - FOR QUALIFIED INVESTIGATORS AND THEIR IRBS ONLY

12.5 Future Use of Stored Specimens

Data collected for this study will be analyzed and stored at Precision Biologics, Inc. After the study is completed, the de-identified, archived data will be transmitted to and stored at Precision Biologics, under the supervision of Dr. Philip Arlen, for use by Precision Biologics.

With the participant's approval and as approved by local IRBs, de-identified biological samples will be stored at Precision Biologics, Inc. with the same goal as outlined in this protocol. These samples could be used for research into the causes of cancer, its complications and other conditions for which individuals with cancer are at increased risk, and to improve treatment. The Precision Biologics laboratory will provide with a code-link that will allow linking the biological specimens with the clinical trial data from each participant, maintaining the masking of the identity of the participant.

During the conduct of the study, an individual participant can choose to withdraw consent to have biological specimens stored for future research. However, withdrawal of consent with regard to biosample storage will not be possible after the study is completed.

When the study is completed, access to study data and/or samples will be provided through Precision Biologics, Inc.

13 DATA HANDLING AND RECORD KEEPING

13.1 Data Collection and Management Responsibilities

Data collection is the responsibility of the clinical trial staff at the site under the supervision of the site PI. The investigator is responsible for ensuring the accuracy, completeness, legibility, and timeliness of the data reported.

All source documents should be completed in a neat, legible manner to ensure accurate interpretation of data. Black ink is required to ensure clarity of reproduced copies. When making changes or corrections, cross out the original entry with a single line, and initial and date the change. DO NOT ERASE, OVERWRITE, OR USE CORRECTION FLUID OR TAPE ON THE ORIGINAL.

Copies of the electronic CRF (eCRF) will be provided for use as source documents and maintained for recording data for each participant enrolled in the study. Data reported in the eCRF derived from source documents should be consistent with the source documents or the discrepancies should be explained and captured in a progress note and maintained in the participant's official electronic study record.

Clinical data (including AEs, concomitant medications, and expected adverse reactions data) and clinical laboratory data will be entered into the EDC, a 21 CFR Part 11-compliant data capture system provided by Precision Biologics, Inc. The data system includes password protection and internal quality checks, such as automatic range checks, to identify data that appear inconsistent, incomplete, or inaccurate. Clinical data will be entered directly from the source documents.

The Investigator, or designated representative, should complete the data entry forms as soon as possible after information is collected, preferably on the same day that a study subject is seen for an examination, treatment, or any other study procedure, **but at least within 2 weeks of study visit**. Any outstanding entries must be completed immediately after the final examination. An explanation should be given for all missing data.

To achieve the primary objective of this study, ongoing collection of all AEs, TEAEs and DLTs will be collected and communicated to the sponsor in a timely fashion.

Subject demographics and key/essential disease baseline characteristics thought to affect outcome, i.e., stratification variables and other prognostic factors will be collected, as available, for all subjects who provide written informed consent.

For subjects who provide informed consent and were not entered/randomized into the study, the reason the subject was not entered/randomized, i.e., did not meet one or more inclusion criteria, met one or more exclusion criteria, or other (e.g., lost to follow up, consent withdrawn), will also be collected and maintained on the IHC Screening Log and/or the Study Screening Log.

The Investigator must sign and date the Investigator's Statement that will be supplied, to endorse the recorded data.

13.1.1 Provisions for Monitoring Data Collection to Ensure Safety of Subjects

As information is gathered from this trial, clinical results will be shared with subjects. Laboratory and clinical data will be frequently gathered and any new significant finding(s) found during the course of the research, which may affect a subject's willingness to participate further, will be explained. Confidentiality of information concerning participants will be maintained, including in all publications and presentations resulting from this study. Names of participants or material identifying participants will not be released without permission, except as such release is required by law.

13.2 Study Records Retention

The Investigator will maintain all study records according to ICH-GCP and applicable regulatory requirement(s). Study documents (including subject records, copies of collected data, study notebook, and pharmacy records) must be kept secured in accordance with Precision Biologics policies. Study documents should be retained for a minimum of 2 years after the last approval of a marketing application in an ICH region and until there are no pending or contemplated marketing applications in an ICH region or until at least 2 years have elapsed since the formal discontinuation of clinical development of the investigational product. These documents should be retained for a longer period, however, if required by local regulations. No records will be destroyed without the written consent of the sponsor, if applicable. It is the responsibility of the sponsor to inform the investigator when these documents no longer need to be retained. There may be other circumstances for which Precision Biologics is required to maintain study records and, therefore, Precision Biologics should be contacted prior to removing study records for any reason. If the Investigator withdraws from the responsibility of keeping the study records, custody must be transferred to a person willing to accept the responsibility. Precision Biologics Inc. must be notified in writing if a custodial change occurs.

CONFIDENTIAL:
NOT TO BE DISCLOSED WITHOUT PERMISSION FROM PRECISION BIOLOGICS - FOR QUALIFIED INVESTIGATORS AND THEIR IRBS ONLY

13.3 Protocol deviations

A protocol deviation is any noncompliance with the clinical trial protocol, GCP, or IRB requirements. The noncompliance may be either on the part of the participant, the investigator, or the study site staff. As a result of deviations, corrective actions are to be developed by the site and implemented promptly.

These practices are consistent with ICH E6:

- 4.5 Compliance with Protocol, sections 4.5.1, 4.5.2, and 4.5.3
- 5.1 Quality Assurance and Quality Control, section 5.1.1
- 5.20 Noncompliance, sections 5.20.1, and 5.20.2.

It is the responsibility of the site to use continuous vigilance to identify and report deviations within 7 working days of identification of the protocol deviation, or within 7 working days of the scheduled protocol-required activity. All deviations must be addressed in study source documents, reported to participating site IRB according to institutional policy and to the Study Sponsor. . The site PI/study staff is responsible for knowing and adhering to their IRB requirements.

13.4 Publication and Data Sharing Policy

This study will comply with the NIH Public Access Policy, which ensures that the public has access to the published results of NIH funded research. It requires scientists to submit final peer-reviewed journal manuscripts that arise from NIH funds to the digital archive PubMed Central upon acceptance for publication.

The International Committee of Medical Journal Editors (ICMJE) member journals have adopted a clinical trials registration policy as a condition for publication. The ICMJE defines a clinical trial as any research project that prospectively assigns human subjects to intervention or concurrent comparison or control groups to study the cause-and-effect relationship between a medical intervention and a health outcome. Medical interventions include drugs, surgical procedures, devices, behavioral treatments, process-of-care changes, and the like. Health outcomes include any biomedical or health-related measures obtained in subjects or participants, including pharmacokinetic measures and adverse events. The ICMJE policy, and the Section 801 of the Food and Drug Administration Amendments Act of 2007, requires that all clinical trials be registered in a public trials registry such as ClinicalTrials.gov, which is sponsored by the National Library of Medicine. Other biomedical journals are considering adopting similar policies. For interventional clinical trials performed under NIH IC grants and cooperative agreements, it is the grantee's responsibility to register the trial in an acceptable registry, so the research results may be considered for publication in ICMJE member journals. The ICMJE does not review specific studies to determine whether registration is necessary; instead, the committee recommends that researchers who have questions about the need to register err on the side of registration or consult the editorial office of the journal in which they wish to publish.

FDAAA mandates that a "responsible party" (i.e., in this study- the sponsor) register and report results of certain "applicable clinical trials":

CONFIDENTIAL:
NOT TO BE DISCLOSED WITHOUT PERMISSION FROM PRECISION BIOLOGICS - FOR QUALIFIED INVESTIGATORS AND THEIR IRBS ONLY

- Trials of Drugs and Biologics: Controlled, clinical investigations, other than Phase I investigations, of a product subject to FDA regulation;

- Trials of Devices: Controlled trials with health outcomes of a product subject to FDA regulation (other than small feasibility studies) and pediatric postmarket surveillance studies.

14 STUDY ADMINISTRATION

14.1 STUDY LEADERSHIP

The Steering Committee will govern the conduct of the study. The Steering Committee will be composed of the Medical Director of Precision Biologics, Inc., the PI of NCI, representatives of the Study Sponsor, the PIs of additional clinical sites after the Phase 1 portion is completed, , and the Scientific Director of the Precision Biologics' Laboratory.

15 CONFLICT OF INTEREST POLICY

The independence of this study from any actual or perceived influence, such as by the pharmaceutical industry, is critical. Therefore any actual conflict of interest of persons who have a role in the design, conduct, analysis, publication, or any aspect of this trial will be disclosed and managed. Furthermore, persons who have a perceived conflict of interest will be required to have such conflicts managed in a way that is appropriate to their participation in the trial. The study Sponsor will apply and require disclosure statements (FORM FDA 3455) from all clinical investigators, and will provide certification (FORM FDA 3454) to the FDA that investigators have no disclosable financial interest or provide detailed information about those financial interests and arrangements (for example, the nature of the contingent payment or the equity holdings of the investigator, or the investigator's spouse or dependent child, that exceeded the threshold) and a description of the steps taken to minimize the potential for bias resulting from the disclosed financial interests and arrangements (21 CFR § 54.4(a)(3))

16 APPENDICES

16.1 APPENDIX A-PERFORMANCE STATUS CRITERIA

**ECOG Performance Status Scale**		**Karnofsky Performance Scale**	
Grade	Descriptions	Percent	Description
0	Normal activity. Fully active, able to carry on all pre-disease performance without restriction.	100	Normal, no complaints, no evidence of disease.
		90	Able to carry on normal activity; minor signs or symptoms of disease.
1	Symptoms, but ambulatory. Restricted in physically strenuous activity, but ambulatory and able to carry out work of a light or sedentary nature (e.g., light housework, office work).	80	Normal activity with effort; some signs or symptoms of disease.
		70	Cares for self, unable to carry on normal activity or to do active work.
2	In bed <50% of the time. Ambulatory and capable of all self-care, but unable to carry out any work activities. Up and about more than 50% of waking hours.	60	Requires occasional assistance, but is able to care for most of his/her needs.
		50	Requires considerable assistance and frequent medical care.
3	In bed >50% of the time. Capable of only limited self-care, confined to bed or chair more than 50% of waking hours.	40	Disabled, requires special care and assistance.
		30	Severely disabled, hospitalization indicated. Death not imminent.
4	100% bedridden. Completely disabled. Cannot carry on any self-care. Totally confined to bed or chair.	20	Very sick, hospitalization indicated. Death not imminent.
		10	Moribund, fatal processes progressing rapidly.
5	Dead.	0	Dead.

CONFIDENTIAL:
NOT TO BE DISCLOSED WITHOUT PERMISSION FROM PRECISION BIOLOGICS - FOR QUALIFIED INVESTIGATORS AND THEIR IRBS ONLY

16.2 APPENDIX B: HYPERSENSITIVITY/INFUSION REACTION ALGORITHM

Hypersensitivity/Infusion Reaction Algorithm
NEO-201

MILD or **MODERATE** Symptoms: **RESTART** infusion or **RETREAT** (as per below)

Preparation and Giving the Drug
Pre-medications*: Give 20-30 minutes prior to infusion
* After 1st dose in dose escalation cohorts

Diphenhydramine- 25-50 mg I.V.
Dexamethasone -10 mg I.V
Ranitidine - 50 mg I.V or equivalent.

Start NEO-201 Infusion at 0.5mg/min

SEVERE Symptoms: DO NOT RETREAT (as per below)

MILD, if any or all of the following symptoms:

- Shortness of breath with minimal change in O2 saturation
- Flushing
- Itching
- Hives
- Chills/Rigors

Then, DO THE FOLLOWING:

- STOP the infusion and NOTIFY physician
- ASSESS vital signs
- OBSERVE and/or Give:

1. Diphenhydramine (Benadryl) 50mg IV push
2. Hydrocortisone (solucortef) 100mg IV push
3. Meperidine HCl (Demerol) (for rigors)

When symptoms resolve **RESTART** at ½ the previous rate.

PB-1801 12-29-16 v2.0

MODERATE, if any or all of the following symptoms occur or symptoms recur

- Shortness of breath with wheezing or drop in O2 saturation
- Chest tightness
- Back pain
- Minimal blood pressure changes requiring only fluid replacement.

Then, DO THE FOLLOWING:

- STOP the infusion and NOTIFY physician
- ASSESS vital signs
- Give:

1. Diphenhydramine (Benadryl) 50mg IV push
2. Hydrocortisone (solucortef) 100mg IV push
3. Oxygen therapy 2L as needed
4. Albuterol Inhaler: 1-2 puffs of Albuterol or Albuterol Inhalation Solution.

When symptoms resolve **RESTART** at ½ the previous rate. If symptoms are prolonged (longer than 2 hours) DO NOT restart, but give/adjust pre-meds for next dose.

SEVERE, if any or all of the following symptoms occur or symptoms recur

- Stridor
- Symptomatic Bronchospasm
- Blood Pressure changes requiring vasopressors.
- ***DO NOT RETREAT***

Then, DO THE FOLLOWING:

- STOP the infusion and NOTIFY physician
- ASSESS vital signs
- Give

1. Diphenhydramine (Benadryl) 50mg IV push
2. Hydrocortisone (solucortef) 100mg IV push
3. Oxygen therapy 2L as needed
4. Albuterol Inhaler: 1-2 puffs of Albuterol or Albuterol Inhalation Solution.
5. Epinephrine if indicated
6. Additional therapy as clinically indicated.

If symptoms meet criteria for "severe," the infusion will be permanently discontinued.

CONFIDENTIAL:
NOT TO BE DISCLOSED WITHOUT PERMISSION FROM PRECISION BIOLOGICS - FOR QUALIFIED INVESTIGATORS AND THEIR IRBS ONLY

16.3 Appendix C: Study Calendar

Procedure	***Screening/ Baseline***	***Cycle 1***					***Subsequent Cycles (even)***			***Subsequent Cycles (odd)***			***End of study visit[1]***	***Post Therapy Follow-up***
		D 1	***D 2***	***D 4, D8 (±48h)***	***D 15 (±48h)***	***D 28[8] (±4d)***	***D 1 (±48h)***	***D 15 (±48h)***	***D 28[8] (±4d)***	***D 1 (±48h)***	***D 15 (±48h)***	***D 28[8] (±4d)***		
History and PE	X	X				X[8]			X[7,8]				X	X
Vital signs	X	X		X	X	X		X	X[7,8]		X	X	X	
Weight and Height (Ht only at screening	X						X[8]			X[8]				
Performance Score	X	X			X	X[8]		X	X[7,8]		X	X	X	
NEO-201 administration		X			X		X	X		X	X			
Laboratory Eval														
• *CBC and diff*	X	X[6]	X[11]	X		X[8]			X[7,8]				X	
• *PT/INR and APTT*	X					X[8]			X[7,8]				X	
• *Chemistry[4]*	X	X[6]				X[8]			X[7,8]				X	
• *CEA level*		X							X[7,8]				X	
• *Thyroid function: T3, T4, TSH*	X												X	
• *Urinalysis*	X	X[6]												
• *Pregnancy test (urine or serum)*	X													
Biopsies	X[9]													
Correlative Research Studies		X[10]	X[10]	X[10]	X[10]				X[10]					
PK/PD	X	X[3]	X[3]	X[3]	X[3]	X[3]	X[3]	X[3]	X[3]					
NIH Advanced Directives Form[2]	X													
Other Specific Assessments														
•														
Radiologic evaluation[5]									X				X	X

Procedure	Screening/ Baseline	Cycle 1					Subsequent Cycles (even)			Subsequent Cycles (odd)			End of study visit[1]	Post Therapy Follow-up
		D 1	D 2	D 4, D8 (±48h)	D 15 (±48h)	D 28[8] (±4d)	D 1 (±48h)	D 15 (±48h)	D 28[8] (±4d)	D 1 (±48h)	D 15 (±48h)	D 28[8] (±4d)		
Response Evaluation[1]	X								X				X	X
Adverse Events		X	→	→	→	→	→	→	→	→	→	→	→	
Concomitant Medications		X	→	→	→	→	→	→	→	→	→	→	→	

1. End of study visit will occur approximately 30 days after the last dose of study drug, or at the time of progression, whichever is later. If the subject cannot return to the Clinical Center for this visit, a request will be made to collect required clinical labs (specify as needed) from a local physician or laboratory. If this is not possible, subjects may be assessed by telephone for symptoms.
2. All subjects ≥ age 18 will be offered the opportunity to complete advanced directives form. This should be done preferably at baseline but can be done at any time during the study as long as the capacity to do so is retained. The completion of the form is strongly recommended, but is not required.
3. Day 1 (Dose 1): pre-dose, immediately after end of infusion (within 3 minutes), 1 hour, 4 hours; Day 2 (24 hours), Day 4 (72 hours), Day 8 (7 days), Day 14 (pre-dose and immediately after end of infusion [within 3 minutes]), Day 29 (pre-dose and immediately after end of infusion [within 3 minutes]), C2D15 (pre-dose and immediately after end of infusion [within 3 minutes]), and C2D29 (Evaluation).
4. Chemistries: BUN, serum creatinine, uric acid, LDH, calcium, magnesium, sodium, chloride, potassium, carbon dioxide, alkaline phosphatase, total bilirubin, direct bilirubin, AST (SGOT), ALT (SGPT), protein, glucose and albumin, C-reactive protein.
5. Radiologic evaluation specific for disease type; to be used consistently throughout the protocol i.e., CT, PET/CT, MRI
6. Prior to initiation of Day1 Cycle 1 dosing, laboratory evaluation will be performed (if screening labs were performed > 72 hours) to include CBC ONLY, Urinalysis, Electrolytes, BUN, creatinine (Results are not required prior to administration of first dose (Cycle 1 Day 1 only) of study drug):
7. End of Cycle (even) evaluations; if subject has SD or better, has not experienced DLT and wishes to continue, after consultation with the sponsor until off treatment/off study criteria (Sections 4.5.1.1 and 4.5.1.2) are met.
8. End of cycle evaluations may also serve as Day 1 evaluations for subjects proceeding to additional cycles.
9. Required only in subjects who do NOT have archived tumor tissue for IHC (see Section 4.1)
10. Correlative samples will be collected at baseline, C1D2 (24 hour after conclusion of infusion), C1D3 (72 hour after conclusion of infusion), C1D14 (pre-dose #2) and C2D29 (evaluation).
11. Daily for the C1 week 1.

CONFIDENTIAL:
NOT TO BE DISCLOSED WITHOUT PERMISSION FROM PRECISION BIOLOGICS - FOR QUALIFIED INVESTIGATORS AND THEIR IRBS ONLY

16.4 APPENDIX D: RESPONSE CRITERIA

For the purposes of this study, subjects should be re-evaluated for response every 8 weeks. In addition to a baseline scan, confirmatory scans should also be obtained 4 weeks following initial documentation of objective response.

Response and progression will be evaluated in this study using the new international criteria proposed by the revised Response Evaluation Criteria in Solid Tumors (RECIST) guideline (version 1.1) [*Eur J Ca* 45:228-247, 2009]. Changes in the largest diameter (unidimensional measurement) of the tumor lesions and the shortest diameter in the case of malignant lymph nodes are used in the RECIST criteria.

16.4.1 Definitions

Evaluable for toxicity: All subjects will be evaluable for toxicity from the time of their first treatment with NEO-201.

Evaluable for objective response: Only those subjects who have measurable disease present at baseline, have received at least one cycle of therapy, and have had their disease re-evaluated will be considered evaluable for response. These subjects will have their response classified according to the definitions stated below. (Note: Subjects who exhibit objective disease progression prior to the end of cycle 1 will also be considered evaluable.)

Evaluable Non-Target Disease Response: Subjects who have lesions present at baseline that are evaluable but do not meet the definitions of measurable disease, have received at least one cycle of therapy, and have had their disease re-evaluated will be considered evaluable for non-target disease. The response assessment is based on the presence, absence, or unequivocal progression of the lesions.

16.4.2 Disease Parameters

Measurable disease: Measurable lesions are defined as those that can be accurately measured in at least one dimension (longest diameter to be recorded) as ≥20 mm by chest x-ray, as ≥10 mm with CT scan, or ≥10 mm with calipers by clinical exam. All tumor measurements must be recorded in millimeters (or decimal fractions of centimeters).

Malignant lymph nodes. To be considered pathologically enlarged and measurable, a lymph node must be ≥15 mm in short axis when assessed by CT scan (CT scan slice thickness recommended to be no greater than 5 mm). At baseline and in follow-up, only the short axis will be measured and followed.

Non-measurable disease. All other lesions (or sites of disease), including small lesions (longest diameter <10 mm or pathological lymph nodes with ≥10 to <15 mm short axis), are considered non-measurable disease. Bone lesions, leptomeningeal disease, ascites, pleural/pericardial effusions, lymphangitis cutis/pulmonitis, inflammatory breast disease, and abdominal masses (not followed by CT or MRI), are considered as non-measurable.

CONFIDENTIAL:
NOT TO BE DISCLOSED WITHOUT PERMISSION FROM PRECISION BIOLOGICS - FOR QUALIFIED INVESTIGATORS AND THEIR IRBS ONLY

Note: Cystic lesions that meet the criteria for radiographically defined simple cysts should not be considered as malignant lesions (neither measurable nor non-measurable) since they are, by definition, simple cysts.

'Cystic lesions' thought to represent cystic metastases can be considered as measurable lesions, if they meet the definition of measurability described above. However, if non-cystic lesions are present in the same subject, these are preferred for selection as target lesions.

Target lesions. All measurable lesions up to a maximum of 2 lesions per organ and 5 lesions in total, representative of all involved organs, should be identified as **target lesions** and recorded and measured at baseline. Target lesions should be selected on the basis of their size (lesions with the longest diameter), be representative of all involved organs, but in addition should be those that lend themselves to reproducible repeated measurements. It may be the case that, on occasion, the largest lesion does not lend itself to reproducible measurement in which circumstance the next largest lesion which can be measured reproducibly should be selected. A sum of the diameters (longest for non-nodal lesions, short axis for nodal lesions) for all target lesions will be calculated and reported as the baseline sum diameters. If lymph nodes are to be included in the sum, then only the short axis is added into the sum. The baseline sum diameters will be used as reference to further characterize any objective tumor regression in the measurable dimension of the disease.

Non-target lesions. All other lesions (or sites of disease) including any measurable lesions over and above the 5 target lesions should be identified as **non-target lesions** and should also be recorded at baseline. Measurements of these lesions are not required, but the presence, absence, or in rare cases unequivocal progression of each should be noted throughout follow-up.

16.4.3 Methods for Evaluation of Measurable Disease

All measurements should be taken and recorded in metric notation using a ruler or calipers. All baseline evaluations should be performed as closely as possible to the beginning of treatment and never more than 4 weeks before the beginning of the treatment.

The same method of assessment and the same technique should be used to characterize each identified and reported lesion at baseline and during follow-up. Imaging-based evaluation is preferred to evaluation by clinical examination unless the lesion(s) being followed cannot be imaged but are assessable by clinical exam.

Clinical lesions: Clinical lesions will only be considered measurable when they are superficial (e.g., skin nodules and palpable lymph nodes) and ≥10 mm diameter as assessed using calipers (e.g., skin nodules). In the case of skin lesions, documentation by color photography, including a ruler to estimate the size of the lesion, is recommended.

Chest x-ray: Lesions on chest x-ray are acceptable as measurable lesions when they are clearly defined and surrounded by aerated lung. However, CT is preferable.

Conventional CT and MRI: This guideline has defined measurability of lesions on CT scan based on the assumption that CT slice thickness is 5 mm or less. If CT scans have slice thickness greater than 5 mm, the minimum size for a measurable lesion should be twice the slice thickness. MRI is also acceptable in certain situations (e.g. for body scans).

CONFIDENTIAL:
NOT TO BE DISCLOSED WITHOUT PERMISSION FROM PRECISION BIOLOGICS - FOR QUALIFIED INVESTIGATORS AND THEIR IRBS ONLY

Use of MRI remains a complex issue. MRI has excellent contrast, spatial, and temporal resolution; however, there are many image acquisition variables involved in MRI, which greatly impact image quality, lesion conspicuity, and measurement. Furthermore, the availability of MRI is variable globally. As with CT, if an MRI is performed, the technical specifications of the scanning sequences used should be optimized for the evaluation of the type and site of disease. Furthermore, as with CT, the modality used at follow-up should be the same as was used at baseline and the lesions should be measured/assessed on the same pulse sequence. It is beyond the scope of the RECIST guidelines to prescribe specific MRI pulse sequence parameters for all scanners, body parts, and diseases. Ideally, the same type of scanner should be used and the image acquisition protocol should be followed as closely as possible to prior scans. Body scans should be performed with breath-hold scanning techniques, if possible.

PET-CT: At present, the low dose or attenuation correction CT portion of a combined PET-CT is not always of optimal diagnostic CT quality for use with RECIST measurements. However, if the site can document that the CT performed as part of a PET-CT is of identical diagnostic quality to a diagnostic CT (with IV and oral contrast), then the CT portion of the PET-CT can be used for RECIST measurements and can be used interchangeably with conventional CT in accurately measuring cancer lesions over time. Note, however, that the PET portion of the CT introduces additional data which may bias an investigator if it is not routinely or serially performed.

Ultrasound: Ultrasound is not useful in assessment of lesion size and should not be used as a method of measurement. Ultrasound examinations cannot be reproduced in their entirety for independent review at a later date and, because they are operator dependent, it cannot be guaranteed that the same technique and measurements will be taken from one assessment to the next. If new lesions are identified by ultrasound in the course of the study, confirmation by CT or MRI is advised. If there is concern about radiation exposure at CT, MRI may be used instead of CT in selected instances.

Endoscopy, Laparoscopy: The utilization of these techniques for objective tumor evaluation is not advised. However, such techniques may be useful to confirm complete pathological response when biopsies are obtained or to determine relapse in trials where recurrence following complete response (CR) or surgical resection is an endpoint.

Tumor markers: Tumor markers alone cannot be used to assess response. If markers are initially above the upper normal limit, they must normalize for a subject to be considered in complete clinical response. Specific guidelines for both CA-125 response (in recurrent ovarian cancer) and PSA response (in recurrent prostate cancer) have been published [*JNCI* 96:487-488, 2004; *J Clin Oncol* 17, 3461-3467, 1999; *J Clin Oncol* 26:1148-1159, 2008]. In addition, the Gynecologic Cancer Intergroup has developed CA-125 progression criteria which are to be integrated with objective tumor assessment for use in first-line trials in ovarian cancer [*JNCI* 92:1534-1535, 2000].

Cytology, Histology: These techniques can be used to differentiate between partial responses (PR) and complete responses (CR) in rare cases (e.g., residual lesions in tumor types, such as germ cell tumors, where known residual benign tumors can remain).

The cytological confirmation of the neoplastic origin of any effusion that appears or worsens during treatment when the measurable tumor has met criteria for response or stable disease is

CONFIDENTIAL:
NOT TO BE DISCLOSED WITHOUT PERMISSION FROM PRECISION BIOLOGICS - FOR QUALIFIED INVESTIGATORS AND THEIR IRBS ONLY

mandatory to differentiate between response or stable disease (an effusion may be a side effect of the treatment) and progressive disease.

FDG-PET: While FDG-PET response assessments need additional study, it is sometimes reasonable to incorporate the use of FDG-PET scanning to complement CT scanning in assessment of progression (particularly possible 'new' disease). New lesions on the basis of FDG-PET imaging can be identified according to the following algorithm:

a. Negative FDG-PET at baseline, with a positive FDG-PET at follow-up is a sign of PD based on a new lesion.
b. No FDG-PET at baseline and a positive FDG-PET at follow-up: If the positive FDG-PET at follow-up corresponds to a new site of disease confirmed by CT, this is PD. If the positive FDG-PET at follow-up is not confirmed as a new site of disease on CT, additional follow-up CT scans are needed to determine if there is truly progression occurring at that site (if so, the date of PD will be the date of the initial abnormal FDG-PET scan). If the positive FDG-PET at follow-up corresponds to a pre-existing site of disease on CT that is not progressing on the basis of the anatomic images, this is not PD.
c. FDG-PET may be used to upgrade a response to a CR in a manner similar to a biopsy in cases where a residual radiographic abnormality is thought to represent fibrosis or scarring. The use of FDG-PET in this circumstance should be prospectively described in the protocol and supported by disease-specific medical literature for the indication. However, it must be acknowledged that both approaches may lead to false positive CR due to limitations of FDG-PET and biopsy resolution/sensitivity.

Note: A ‘positive’ FDG-PET scan lesion means one which is FDG avid with an uptake greater than twice that of the surrounding tissue on the attenuation corrected image.

16.4.4 Response Criteria

16.4.4.1 Evaluation of Target Lesions

Complete Response (CR): Disappearance of all target lesions. Any pathological lymph nodes (whether target or non-target) must have reduction in short axis to <10 mm.

Partial Response (PR): At least a 30% decrease in the sum of the diameters of target lesions, taking as reference the baseline sum of diameters.

Progressive Disease (PD): At least a 20% increase in the sum of the diameters of target lesions, taking as reference the smallest sum on study (this includes the baseline sum if that is the smallest on study). In addition to the relative increase of 20%, the sum must also demonstrate an absolute increase of at least 5 mm. (Note: the appearance of one or more new lesions is also considered progressions).

Stable Disease (SD): Neither sufficient shrinkage to qualify for PR nor sufficient increase to qualify for PD, taking as reference the smallest sum of diameters while on study.

16.4.4.2 Evaluation of Non-Target Lesions

Complete Response (CR): Disappearance of all non-target lesions and normalization of tumor marker level. All lymph nodes must be non-pathological in size (<10 mm short axis).

Note: If tumor markers are initially above the upper normal limit, they must normalize for a subject to be considered in complete clinical response.

Non-CR/Non-PD: Persistence of one or more non-target lesion(s) and/or maintenance of tumor marker level above the normal limits.

Progressive Disease (PD): Appearance of one or more new lesions and/or *unequivocal progression* of existing non-target lesions. *Unequivocal progression* should not normally trump target lesion status. It must be representative of overall disease status change, not a single lesion increase.

Although a clear progression of "non-target" lesions only is exceptional, the opinion of the treating physician should prevail in such circumstances, and the progression status should be confirmed at a later time by the review panel (or Principal Investigator).

16.4.4.3 Evaluation of Best Overall Response

The best overall response is the best response recorded from the start of the treatment until disease progression/recurrence (taking as reference for progressive disease the smallest measurements recorded since the treatment started). The subject's best response assignment will depend on the achievement of both measurement and confirmation criteria.

For Subjects with Measurable Disease (i.e., Target Disease)

<table>
<tr><th>Target Lesions</th><th>Non-Target Lesions</th><th>New Lesions</th><th>Overall Response</th><th>Best Overall Response when Confirmation is Required*</th></tr>
<tr><td>CR</td><td>CR</td><td>No</td><td>CR</td><td>≥4 wks. Confirmation**</td></tr>
<tr><td>CR</td><td>Non-CR/Non-PD</td><td>No</td><td>PR</td><td rowspan="3">≥4 wks. Confirmation**</td></tr>
<tr><td>CR</td><td>Not evaluated</td><td>No</td><td>PR</td></tr>
<tr><td>PR</td><td>Non-CR/Non-PD/not evaluated</td><td>No</td><td>PR</td></tr>
<tr><td>SD</td><td>Non-CR/Non-PD/not evaluated</td><td>No</td><td>SD</td><td>Documented at least once ≥4 wks. from baseline**</td></tr>
<tr><td>PD</td><td>Any</td><td>Yes or No</td><td>PD</td><td rowspan="3">no prior SD, PR or CR</td></tr>
<tr><td>Any</td><td>PD***</td><td>Yes or No</td><td>PD</td></tr>
<tr><td>Any</td><td>Any</td><td>Yes</td><td>PD</td></tr>
</table>

CONFIDENTIAL:
NOT TO BE DISCLOSED WITHOUT PERMISSION FROM PRECISION BIOLOGICS - FOR QUALIFIED INVESTIGATORS AND THEIR IRBS ONLY

*	See RECIST 1.1 manuscript for further details on what is evidence of a new lesion.
**	Only for non-randomized trials with response as primary endpoint.
***	In exceptional circumstances, unequivocal progression in non-target lesions may be accepted as disease progression.
Note:	Subjects with a global deterioration of health status requiring discontinuation of treatment without objective evidence of disease progression at that time should be reported as "*symptomatic deterioration.*" Every effort should be made to document the objective progression even after discontinuation of treatment.

16.4.5 Duration of Response

Duration of overall response: The duration of overall response is measured from the time measurement criteria are met for CR or PR (whichever is first recorded) until the first date that recurrent or progressive disease is objectively documented (taking as reference for progressive disease the smallest measurements recorded since the treatment started).

The duration of overall CR is measured from the time measurement criteria are first met for CR until the first date that progressive disease is objectively documented.

Duration of stable disease: Stable disease is measured from the start of the treatment until the criteria for progression are met, taking as reference the smallest measurements recorded since the treatment started, including the baseline measurements.

16.4.6 Progression-Free Survival

PFS is defined as the duration of time from start of treatment to time of progression or death, whichever occurs first.

CONFIDENTIAL:
NOT TO BE DISCLOSED WITHOUT PERMISSION FROM PRECISION BIOLOGICS - FOR QUALIFIED INVESTIGATORS AND THEIR IRBS ONLY

17 INVESTIGATOR AGREEMENT

Protocol Title: PHASE 1 STUDY OF NEO-201 IN ADULTS WITH CHEMO-RESISTANT SOLID TUMORS

Protocol Number: PB-1801

Version Date: July 5, 2019

I have received and reviewed the Investigator Brochure for NEO-201.

I have read this protocol and agree that the study is ethical.

I agree to conduct the study as outlined and in accordance with all applicable regulations and guidelines.

I agree to inform all who assist me in the conduct of this study of their responsibilities and obligations.

I agree to maintain the confidentiality of all information received or developed in connection with this protocol and Investigator's Brochure.

______________________________________ ____________________

Signature of Principal Investigator Date

____

Name of Principal Investigator (printed or typed)

18 REFERENCES

1. Gold, P. and S.O. Freedman, *Specific carcinoembryonic antigens of the human digestive system.* J Exp Med, 1965. 122(3): p. 467-81.

2. Sorensen, C.G., et al., *The diagnostic accuracy of carcinoembryonic antigen to detect colorectal cancer recurrence - A systematic review.* Int J Surg, 2016. 25: p. 134-44.

3. Gebauer, F., et al., *Carcinoembryonic antigen-related cell adhesion molecules (CEACAM) 1, 5 and 6 as biomarkers in pancreatic cancer.* PLoS One, 2014. 9(11): p. e113023.

4. Grunnet, M. and J.B. Sorensen, *Carcinoembryonic antigen (CEA) as tumor marker in lung cancer.* Lung Cancer, 2012. 76(2): p. 138-43.

5. Bast, R.C., Jr., et al., *Monitoring human ovarian carcinoma with a combination of CA 125, CA 19-9, and carcinoembryonic antigen.* Am J Obstet Gynecol, 1984. 149(5): p. 553-9.

6. Molina, R., et al., *Prospective evaluation of carcinoembryonic antigen (CEA) and carbohydrate antigen 15.3 (CA 15.3) in patients with primary locoregional breast cancer.* Clin Chem, 2010. 56(7): p. 1148-57.

7. Ebert, W., T. Muley, and P. Drings, *Does the assessment of serum markers in patients with lung cancer aid in the clinical decision making process?* Anticancer Res, 1996. 16(4B): p. 2161-8.

8. Beauchemin, N. and A. Arabzadeh, *Carcinoembryonic antigen-related cell adhesion molecules (CEACAMs) in cancer progression and metastasis.* Cancer Metastasis Rev, 2013. 32(3-4): p. 643-71.

9. Obrink, B., *CEA adhesion molecules: multifunctional proteins with signal-regulatory properties.* Curr Opin Cell Biol, 1997. 9(5): p. 616-26.

10. Edmiston, K.H., et al., *In vivo induction of murine cytokine production by carcinoembryonic antigen.* Cancer Res, 1997. 57(19): p. 4432-6.

11. Li, Y., et al., *Carcinoembryonic antigen interacts with TGF-{beta} receptor and inhibits TGF-{beta} signaling in colorectal cancers.* Cancer Res, 2010. 70(20): p. 8159-68.

12. Samara, R.N., L.M. Laguinge, and J.M. Jessup, *Carcinoembryonic antigen inhibits anoikis in colorectal carcinoma cells by interfering with TRAIL-R2 (DR5) signaling.* Cancer Res, 2007. 67(10): p. 4774-82.

13. Camacho-Leal, P. and C.P. Stanners, *The human carcinoembryonic antigen (CEA) GPI anchor mediates anoikis inhibition by inactivation of the intrinsic death pathway.* Oncogene, 2008. 27(11): p. 1545-53.

14. Poola, I., et al., *Expression of carcinoembryonic antigen cell adhesion molecule 6 oncoprotein in atypical ductal hyperplastic tissues is associated with the development of invasive breast cancer.* Clin Cancer Res, 2006. 12(15): p. 4773-83.

15. **Lewis-Wambi, J.S., et al., *Overexpression of CEACAM6 promotes migration and invasion of oestrogen-deprived breast cancer cells.* Eur J Cancer, 2008. 44(12): p. 1770-9.**

16. **Kuijpers, T.W., et al., *CD66 nonspecific cross-reacting antigens are involved in neutrophil adherence to cytokine-activated endothelial cells.* J Cell Biol, 1992. 118(2): p. 457-66.**

17. **Duxbury, M.S., et al., *CEACAM6 gene silencing impairs anoikis resistance and in vivo metastatic ability of pancreatic adenocarcinoma cells.* Oncogene, 2004. 23(2): p. 465-73.**

18. **Abdul-Wahid, A., et al., *A focused immune response targeting the homotypic binding domain of the carcinoembryonic antigen blocks the establishment of tumor foci in vivo.* Int J Cancer, 2012. 131(12): p. 2839-51.**

19. **Berinstein, N.L., *Carcinoembryonic antigen as a target for therapeutic anticancer vaccines: a review.* J Clin Oncol, 2002. 20(8): p. 2197-207.**

20. **Gulley, J.L., et al., *Pilot study of vaccination with recombinant CEA-MUC-1-TRICOM poxviral-based vaccines in patients with metastatic carcinoma.* Clin Cancer Res, 2008. 14(10): p. 3060-9.**

21. **Blumenthal, R.D., H.J. Hansen, and D.M. Goldenberg, *Inhibition of adhesion, invasion, and metastasis by antibodies targeting CEACAM6 (NCA-90) and CEACAM5 (Carcinoembryonic Antigen).* Cancer Res, 2005. 65(19): p. 8809-17.**

22. **Shibata, S., et al., *A phase I study of a combination of yttrium-90-labeled anti-carcinoembryonic antigen (CEA) antibody and gemcitabine in patients with CEA-producing advanced malignancies.* Clin Cancer Res, 2009. 15(8): p. 2935-41.**

23. **Mayer, A., et al., *A phase I study of single administration of antibody-directed enzyme prodrug therapy with the recombinant anti-carcinoembryonic antigen antibody-enzyme fusion protein MFECP1 and a bis-iodo phenol mustard prodrug.* Clin Cancer Res, 2006. 12(21): p. 6509-16.**

24. **Hollinshead, A., et al., *Skin-reactive soluble antigen from intestinal cancer-cell-membranes and relationship to carcinoembryonic antigens.* Lancet, 1970. 1(7658): p. 1191-5.**

25. **Hollinshead, A.C., et al., *Separation of skin reactive intestinal cancer antigen from the carcinoembryonic antigen of Gold.* Science, 1972. 177(4052): p. 887-9.**

26. **Hollinshead, A., et al., *Specific active immunotherapy in patients with adenocarcinoma of the colon utilizing tumor-associated antigens (TAA). A phase I clinical trial.* Cancer, 1985. 56(3): p. 480-9.**

27. **Aubry, J., S. Cohen-Kaminsky, and S. Berrih-Aknin, *Differential expression of carcinoembryonic antigens and non cross-reacting antigens in the human thymus. Analysis on frozen sections and cultured epithelial cells using monoclonal antibodies.* Thymus, 1990. 15(2): p. 107-23.**

CONFIDENTIAL:
NOT TO BE DISCLOSED WITHOUT PERMISSION FROM PRECISION BIOLOGICS - FOR QUALIFIED INVESTIGATORS AND THEIR IRBS ONLY

28. **Ballesta, A.M., et al., *Carcinoembryonic antigen in staging and follow-up of patients with solid tumors.* Tumour Biol, 1995. 16(1): p. 32-41.**

29. **Chapin, C., et al., *Distribution and surfactant association of carcinoembryonic cell adhesion molecule 6 in human lung.* Am J Physiol Lung Cell Mol Physiol, 2012. 302(2): p. L216-25.**

30. **Garcia, M., et al., *Carcinoembryonic antigen has a different molecular weight in normal colon and in cancer cells due to N-glycosylation differences.* Cancer Res, 1991. 51(20): p. 5679-86.**

31. **Ghazizadeh, M., S. Kagawa, and K. Kurokawa, *Immunohistochemical studies of human renal cell carcinomas for ABO(H) blood group antigens, T antigen-like substance and carcinoembryonic antigen.* J Urol, 1985. 133(5): p. 762-6.**

32. **Hammarstrom, S., *The carcinoembryonic antigen (CEA) family: structures, suggested functions and expression in normal and malignant tissues.* Semin Cancer Biol, 1999. 9(2): p. 67-81.**

33. **Hampton, R., et al., *Differential expression of carcinoembryonic antigen (CEA) splice variants in whole blood of colon cancer patients and healthy volunteers: implication for the detection of circulating colon cancer cells.* Oncogene, 2002. 21(51): p. 7817-23.**

34. **Isaacson, P. and M.A. Judd, *Carcinoembryonic antigen (CEA) in the normal human small intestine: a light and electron microscopic study.* Gut, 1977. 18(10): p. 786-91.**

35. **Matsuoka, Y., et al., *Normal bronchial mucus contains high levels of cancer-associated antigens, CA125, CA19-9, and carcinoembryonic antigen.* Cancer, 1990. 65(3): p. 506-10.**

36. **Metze, D., et al., *Glycoproteins of the carcinoembryonic antigen (CEA) family are expressed in sweat and sebaceous glands of human fetal and adult skin.* J Invest Dermatol, 1996. 106(1): p. 64-9.**

37. **Nap, M., et al., *Specificity and affinity of monoclonal antibodies against carcinoembryonic antigen.* Cancer Res, 1992. 52(8): p. 2329-39.**

38. **Nap, M., et al., *Immunohistochemistry of carcino-embryonic antigen in the embryo, fetus and adult.* Tumour Biol, 1988. 9(2-3): p. 145-53.**

39. **Nouwen, E.J., et al., *Immunohistochemical localization of placental alkaline phosphatase, carcinoembryonic antigen, and cancer antigen 125 in normal and neoplastic human lung.* Cancer Res, 1986. 46(2): p. 866-76.**

40. **Scholzel, S., et al., *Carcinoembryonic antigen family members CEACAM6 and CEACAM7 are differentially expressed in normal tissues and oppositely deregulated in hyperplastic colorectal polyps and early adenomas.* Am J Pathol, 2000. 156(2): p. 595-605.**

41. **Shevchuk, M.M., C.M. Fenoglio, and R.M. Richart, *Carcinoembryonic antigen localization in benign and malignant transitional epithelium.* Cancer, 1981. 47(5): p. 899-905.**

**CONFIDENTIAL:
NOT TO BE DISCLOSED WITHOUT PERMISSION FROM PRECISION BIOLOGICS - FOR QUALIFIED INVESTIGATORS AND THEIR IRBS ONLY**

42. **Singer, B.B., et al., *Carcinoembryonic antigen-related cell adhesion molecule 1 expression and signaling in human, mouse, and rat leukocytes: evidence for replacement of the short cytoplasmic domain isoform by glycosylphosphatidylinositol-linked proteins in human leukocytes.* J Immunol, 2002. 168(10): p. 5139-46.**

43. **Bordes, M., S. Knobel, and F. Martin, *Carcinoembryonic antigen (CEA) and related antigens in blood cells and hematopoietic tissues.* Eur J Cancer, 1975. 11(11): p. 783-6.**

44. **Taube, J.M., et al., *Colocalization of inflammatory response with B7-h1 expression in human melanocytic lesions supports an adaptive resistance mechanism of immune escape.* Sci Transl Med, 2012. 4(127): p. 127ra37.**

45. **Dong, H., et al., *Tumor-associated B7-H1 promotes T-cell apoptosis: a potential mechanism of immune evasion.* Nat Med, 2002. 8(8): p. 793-800.**

46. **Patel, S.P. and R. Kurzrock, *PD-L1 Expression as a Predictive Biomarker in Cancer Immunotherapy.* Mol Cancer Ther, 2015. 14(4): p. 847-56.**

47. **Grosso, J., Horak, C.E., Inzunza, D., et al. *Association of tumor PD-L1 expression and immune biomarkers with clinical activity in patients (pts) with advanced solid tumors treated with nivolumab (anti-PD-1; BMS-936558; ONO-4538).* in *ASCO 2013.* 2013. Chicago IL: J Clin Oncol.**

48. ***HERCEPTIN [trastuzumab] package insert.* 2016, Genentech, Inc.: San Francisco, CA.**

49. ***Good Review Practice: Clinical Review of Investigational New Drug Applications*, O.o.N.D.i.t.C.f.D.E.a. Research, Editor. 2013.**
