# Supplementary material for: Correction: First-in-human phase 1 clinical trial of anti-core 1 O-glycans targeting monoclonal antibody NEO-201 in treatment-refractory solid tumors
Source: J Exp Clin Cancer Res. 2023 Apr 26;42:102. doi: 10.1186/s13046-023-02668-3 (PMC10131449; doi:10.1186/s13046-023-02668-3)
Supplement: Supplementary file 2 — Additional file 8: NEO-201 protocol. [file 13046_2023_2668_MOESM2_ESM.docx]

**Pharmacokinetic analyses**

The NEO-201 concentration in serum of cancer patients was determined by a fully-validated enzyme-linked immunosorbent assay (ELISA) method with a linear range of 0.25 – 2.0 mg/L (250 – 2000 ng/mL) performed by the central laboratory at the National Cancer Institute (NCI) Clinical Pharmacology Program.

In brief, the ELISA entails coating 96-well plates overnight at 4°C with 100µL/well of 4 μg/mL NEO-201 capturing antibody (7E4-1) diluted in 0.1M sodium phosphate, 0.15M NaCl, pH 7.2 (Thermo Fisher Scientific, Waltham, MA, USA). The following day, plates were washed 5 times with 1X Tris-buffered saline Tween (TBST) and then blocked with 300µL/well of 1X TBS with 1% Casein (Bio-Rad, Hercules, CA, USA) for 1.5 h at 37°C. Plates were then washed 5 times with 1X TBST and then 130µL/well of serum sample and calibrators were added to the plates. Plates were incubated for 2h at 37°C.

Plates were then washed 5 times with 1X TBST and then 100µL/well of 7E4-1 2μg/mL Biotin Conjugate Solution was added to the plates. Plates were incubated for 1hr at 37°C, then washed 5 times with 1X TBST and then 100µL/well of Streptavidin-HRP Conjugate (BD Bioscience, San Jose, CA, USA) at a 1:20,000 dilution was added to the plates. Plates were incubated for 1h at 37°C, then washed 5 times with 1X TBST. 100µL/well of tetramethylbenzidine (TMB) Ultra (Thermo Fisher Scientific) substrate solution was then added. Plates were immediately read at 650 nm using SpectraMax iD3 Plate Reader (Molecular Devices, Sunnyvale, CA, USA). Once the absorbance of the highest standard calibrator reached 1.5 OD units the reaction was stopped by adding 100µL/well of 1M H2SO4, and absorption at 450nm was read using SpectraMax iD3 Plate Reader. Calibration curves were analyzed simultaneously with QC samples containing NEO-201. The procedure was performed using two calibrator replicates per standard concentration (250, 500, 750, 1000, 1500, 2000 ng/mL).

QC samples were spiked into human serum at three concentrations (300 [LQC], 400 [MQC], and 900 [HQC] μg/mL) in 5 replicates, then diluted 500-fold (600 [LQC], 800 [MQC], 1800 [HQC] ng/mL) for analysis. The QC concentrations were selected to span the range of concentrations evaluated as specified in the FDA guidance document post dilution: low QC was less than or equal to 3x the LLOQ, mid QC was approximately the geometric mean of the entire standard curve; high QC was between 75-90% of the upper limit of quantitation (ULOQ).

Accuracy and precision were evaluated by determination of NEO-201 in five replicates at five different concentrations of QCs (HQC, MQC, LQC, LLOQC, ULOQC) samples analyzed over four different days. HQC, MQC, and LQCs were spiked with NEO-201 in 500-fold excess, then diluted 500-fold to lower the ODs in order to match the standard curve concentrations. Accuracy (DEV) for NEO-201 was defined as percent difference between the mean observed concentration and the nominal concentration: QC samples were considered reliable if the deviation from nominal value for at least 75% of the samples at each concentration were within ±20% and ±25% for LLOQC and ULOQC.

The LLOQ was defined as the lowest concentration of NEO-201 that could be reliably and reproducibly measured with concentration determinations performed in replicates of at least five. To determine the LLOQ, pooled serum samples spiked to contain 250 ng/ml were run on four different days. The LLOQ had to have a precision of ≤ 25% and be distinct from the blank OD. The LLOQ was determined to be 250 ng/mL. Calibration curves were constructed by using a four-parameter logistic regression model (4PL) using GraphPad Prism.

A noncompartmental approach to PK analysis was employed using Phoenix WinNonlin v8.3 (Certara Corp, Princeton, NJ) that was validated per FDA 21CFR Part 11 regulations. The maximum plasma concentration (Cmax) and the time of maximum plasma concentration (Tmax) were recorded as observed values. The area under the concentration-time curve (AUC) from time zero to the time of the final quantifiable sample (AUClast) was calculated using the linear-up/log-down trapezoidal method. AUCinf (the AUC from time zero to infinity) was calculated by extrapolation by dividing Clast (the last measurable drug concentration) by the rate constant of the terminal phase, λz. This constant was determined from the slope of the terminal phase of the concentration-time curve using uniformly weighted least-squares as the estimation procedure and acceptance criteria of i) r2 > 0.9, ii) includes > 3 time points in the terminal phase. All time points used in this PK analysis were actual time elapsed from infusion start. Estimated PK parameters following the first dose included the volume of distribution during the terminal phase (Vz) and the systemic clearance (CL), which was calculated as absolute dose divided by AUCINF. If the amount of AUCINF that is extrapolated exceeds >25%, then the clearance estimates for those patients will be flagged and excluded from statistical summaries. To assess C1D1 dose linearity (aka dose proportionality), the actual dose amount infused and the Cmax or AUClast were both natural log-transformed and plotted against each other (XY plot). Linear regression analysis was then performed to assess the dose linearity of C1D1 NEO-201, where PK sampling was dense enough for AUC assessment. For sparse sampling, accumulation (Rcmax) was assessed by dividing the end of infusion Cmax concentrations at steady-state (cycle 2 or later) compared to first dose EOI Cmax.

**Immunohistochemistry (IHC)**

A qualitative IHC staining system was used to identify the expression of the antigen recognized by NEO-201 in formalin-fixed, paraffin-embedded (FFPE) neoplastic tissues from cancer patients. To have the best accuracy and reproducibility in the IHC, automated immunohistochemical stainers were employed. In this regard, cancer patient tissues were run and analyzed using the Leica Bond Max Automated Immunohistochemical Staining Procedure. Briefly, slides containing FFPE tissues were first deparaffinized and rehydrated before proceeding with the IHC staining protocol in order to avoid a poor staining of the section. After deparaffinization and rehydration 150 µL Epitope Retrieval 2 (Leica Biosystems, Buffalo Grove, IL, USA) was added at room temperature (RT). Then, slides containing tissues were heated at 100°C for 25 minutes and then washed with Bond Wash Solution (Leica Biosystem) in order to break protein cross-linking surrounding the antigen of interest. Then, 150 µL Peroxide Block was added to the slides for 5 minutes at RT to block non-specific binding. Slides were then washed with Bond Wash Solution and then 150 µL of the naked murine version of NEO-201 (m16C3) was added as primary antibody at 1:1,000 dilution for 30 minutes at RT. Slides were then washed with Bond Wash Solution and Bond Polymer Refine Detection Kit (Leica Biosystem) was used as a detection system. Slides were then coverslipped using the Tissue-Tek Glas g2 Automated Glass Coverslipper (Sakura Finetek Inc., Torrance, CA, USA). All slides were read and analyzed at the NCI Clinical IHC Laboratory (NIH, USA) using Leica Bond Max (Leica Biosystem).

A minimum of 10% of tumor cells in cancer tissues stained at a minimum of 2+ or 3+ intensity of staining with m16C3 antibody were considered positive for the expression of the antigen recognized by NEO-201. Cancer tissue with 0 or 1+ staining with m16C3 and underlying stromal tissue with 0 intensity of staining with m16C3 were considered negative for the expression of the antigen recognized by NEO-201. In this study, colon carcinoma with 3+ intensity of staining and adjacent colonic mucosa stained at background level of 1+ intensity were used as positive controls for m16C3 antibody in each run. No Negative control was used since use of negative control is not standard practice for immunohistochemistry laboratories. Background staining of normal tissue is considered at 1+ intensity.

**NK cells and regulatory T cells (Tregs) phenotype analysis**

For NK phenotype analysis, blood samples were drawn prior to the first infusion dose on C1D1, 72 h post EOI, 14 days post EOI and prior to C3D1. For Tregs phenotype analysis blood samples were drawn prior to the first infusion dose on C1D1, 14 days post EOI and prior to C3D1. Isolated PBMCs were cryopreserved in cryovials containing 95% human AB serum + 10% DMSO in liquid nitrogen. The day of the flow cytometry PBMCs were thawed, washed with 1X phosphate buffered saline (PBS) and counted. Cells (1.0 × 106) were incubated with 1 μL per test of LIVE/DEAD Fixable Aqua (Thermo Fisher Scientific) in 1X PBS for 30 min at 4°C to accomplish live versus dead cell discrimination. Cells were then centrifuged, washed twice with cold 1X PBS and incubated with 2-5 μL of Human TruStain FcX™ (BioLegend, San Diego, CA, USA) in 100 uL of 1X PBS at room temperature for 5-10 minutes. Then PBMCs were stained with primary anti-human mAbs in 1X PBS + 1% BSA (Teknova, Hollister, CA, USA) for 30 minutes at 4°C. To detect the NK surface markers PBMCs were labeled with following antibodies: CD56 PE (clone 5.1H11), CD16 PerCP-Cy5.5 (clone 3G8), NKG2D BV421 (clone 1D11), NKp46 FITC (clone 9E2) (BioLegend), CD107a APC-H7 (clone H4A3) (BD Bioscience), CEACAM-1 APC (clone 283340) (VWR, Radnor, PA, USA). To detect surface Tregs markers and to evaluate the reactivity of NEO-201 to human Tregs, PBMCs were labeled with the following anti-human mAbs: CD4 FITC (clone OKT4), CD127 APC (clone A019D5), CD15s PE (clone FH6), NEO-201 Pacific Blue (BioLegend), CD25 APC-H7 (clone M-A251) (BD Bioscience).

For Tregs analysis, after staining surface markers, cells were washed twice with cold 1X PBS and incubated with 1mL of Fix/Perm Solution (eBioscience™ Foxp3 / Transcription Factor Staining Buffer Set, Thermo Fisher Scientific) for 1h at 4°C to allow the detection of intracellular transcription factors. After incubation, cells were washed twice with 2 mL of 1X Permeabilization Buffer (eBioscience™ Foxp3 / Transcription Factor Staining Buffer Set, Thermo Fisher Scientific) and then stained in 100 uL of 1X Permeabilization Buffer for 1h at RT in the dark with 2-4µL/sample of the anti-human Foxp3 PerCP-Cy5.5 mAb (clone 236A/E7, BD Biosciences).

After staining, cells were washed twice with cold 1X PBS and examined using a FACSVerse flow cytometer (BD Biosciences). Analysis of cellular fluorescence was performed using BD FACSuite software (BD Biosciences) and FlowJo 10.8.1. Positivity was determined by using fluorescence-minus-one controls.
